# Deep and quantitative proteomic profiling of low-volume mouse serum in a cross-sectional analysis of aging

**DOI:** 10.1038/s43587-026-01242-8

**Published:** 2026-11-02

**Authors:** Amit K. Dey, Bradley Olinger, Mozhgan Boroumand, Maria Emilia Fernandez, Study of Longitudinal Aging in Mice (SLAM) Investigators, Maria Emilia Fernandez, Rafael de Cabo, Simonetta Camandola, Nathan L. Price, Nathan Basisty, Simonetta Camandola, Nathan L. Price, Rafael de Cabo, Nathan Basisty

**Affiliations:** Translational Gerontology Branch, National Institute on Aging, NIH, Baltimore, MD USA

**Keywords:** Proteomics, Proteomic analysis, Mass spectrometry, Ageing, Data processing

## Abstract

Assessing and validating circulating biomarkers is essential for developing preclinical measures of biological aging in mice. However, comprehensive serum proteomics are limited by technical challenges and low sample volumes. Here we present a quantitative nanoparticle-based enrichment workflow coupled with data-independent acquisition mass spectrometry for deep proteomic profiling of low-volume mouse serum. This approach increased proteomic coverage by 3–6-fold with median coefficient of variation <10%. In 30 mice aged 12, 24 and 30 months, we uncovered 3,992 protein groups from 20 µl of serum and revealed age- and sex-specific changes, and capturing senescence-associated circulating proteins. With 1 µl additional serum,the proximity extension assay quantified 43 cytokines not detected by mass spectrometry. Comparison with plasma proteomics from the Baltimore Longitudinal Study of Aging identified conserved age-associated circulating proteins, several of which were linked to frailty in humans, notably COL6A3. These results establish a scalable approach for discovering aging biomarkers in sample-limited mouse studies with translational relevance to human health.

## Abstract (web summary)

To address the technically challenging task of profiling the age-associated blood proteome in sample-limited mouse studies, the authors present a quantitative nanoparticle-based enrichment workflow coupled with data-independent acquisition mass spectrometry to identify key age-associated circulating proteins with relevance to human health.

## Main

Blood-based specimens such as serum are rich in proteins originating from diverse organs that offer valuable clinical potential^1,2^ for biomarker discovery due to ease of repeated collections with minimal invasiveness. Work from our group and others have highlighted that blood proteins reflect aging-related physiological changes in human clinical studies^3–7^. The full potential of using the circulating proteome to measure aging and predict health in humans has not been fully realized due to the practical challenges of longitudinal sampling across the human lifespan^8^. However, mouse models, owing to their short lifespans, are useful surrogates for rigorously exploring longitudinal changes in the circulating proteome throughout lifespan and formulating strategies for quantifying age-related organismal declines with protein biomarkers in humans^9–11^. Despite this promise, comprehensive serum biomarker assessment is lacking in mice for aging studies. Blood proteins span an extreme dynamic range—exceeding ten orders of magnitude^12^—making it difficult to detect low-abundance proteins using mass spectrometry (MS), which are often the most biologically informative. These challenges are amplified in mice, where blood volume is limited compared to human blood specimens^13^, and particularly when longitudinal sampling is desired or is needed for multiple assays. A truly rigorous assessment of aging biomarkers requires a comprehensive and longitudinal assessment of aging-related phenotypes in parallel with sample collections to make associations of protein changes with phenotypes and phenotype trajectories.

Recent advances in, liquid chromatography–mass spectrometry (LC–MS), along with advanced sample preparation strategies and computational tools, have substantially improved proteome depth, sensitivity and throughput for biomarker discovery^14^. Among these developments, nanoparticle (NP)-based protein corona enrichment has emerged as a promising strategy to reduce sample dynamic range and improve the detection of low-abundance proteins^15^. In doing so, NP-based enrichment coupled with LC–MS-like data-independent acquisition (DIA) permits comprehensive, reproducible and deep plasma proteomics at a scale required for biomarker studies^16–18^, enabling the reproducible identification and quantification of upwards of 6,000 proteins in human plasma. Despite its impressive depth, MS struggles with detection of cytokines and other key players in ‘inflammaging’^19^, owing to several factors including low endogenous concentrations (in the picograms per milliliter range), transient secretion, short half-life and the ability to generate few unique and ionizable tryptic peptides^20^. Sensitivity is further compromised by matrix interferences and inefficient peptide ionization^21^. To overcome limitations in cytokine detection by MS, complementary affinity-based methods such as proximity extension assays (PEA) offer multiplexed quantification of circulating cytokines, detecting up to 48 cytokines with minimal volume requirements (1 µl of mouse serum). Multiple studies highlight the potential benefits of integrating untargeted LC–MS with targeted immunoassay PEA or aptamer-based assays, offering valuable complementarity for a biologically relevant gain in proteome coverage^22–25^.

The present study aims to demonstrate the feasibility, quantitative accuracy and biological sensitivity of an NP-based LC–MS workflow for deep proteomic profiling of low-volume mouse serum. Although the Study of Longitudinal Aging in Mice (SLAM) is an ongoing longitudinal aging cohort^11^, designed to capture repeated functional, phenotypic and biological variables across the lifespan, the proteomic data analyzed here derive from a cross-sectional subset of SLAM, with different mice sampled at 12 (young), 24 (old) and 30 (geriatric) months. Our intention is therefore not to infer within-individual aging trajectories but rather evaluate whether deep, quantitative serum proteomics can be achieved in a sample-limited context relevant to longitudinal aging studies. We benchmarked the quantitative performance of NP-based MS using defined plasma mixtures, optimize protein-level quantification across NP fractions and integrate untargeted LC–MS with a targeted 48-plex PEA cytokine panel using minimal additional serum. We then apply this combined workflow to identify age- and sex-associated proteomic changes in mouse serum. Finally, to assess translational relevance, we compare age-associated circulating proteins identified in mice with human plasma proteomic data from the Baltimore Longitudinal Study of Aging (BLSA). Together, this study provides a scalable and reproducible framework for deep serum proteomics in mice using low sample volumes, establishes the complementary value of integrating MS and targeted cytokine assays and lays the methodological foundation for future longitudinal investigations of aging biology and biomarker discovery.

## Results

### Qualitative and quantitative evaluation of a NP-coupled serum proteomics workflow

**Fig. 1:**
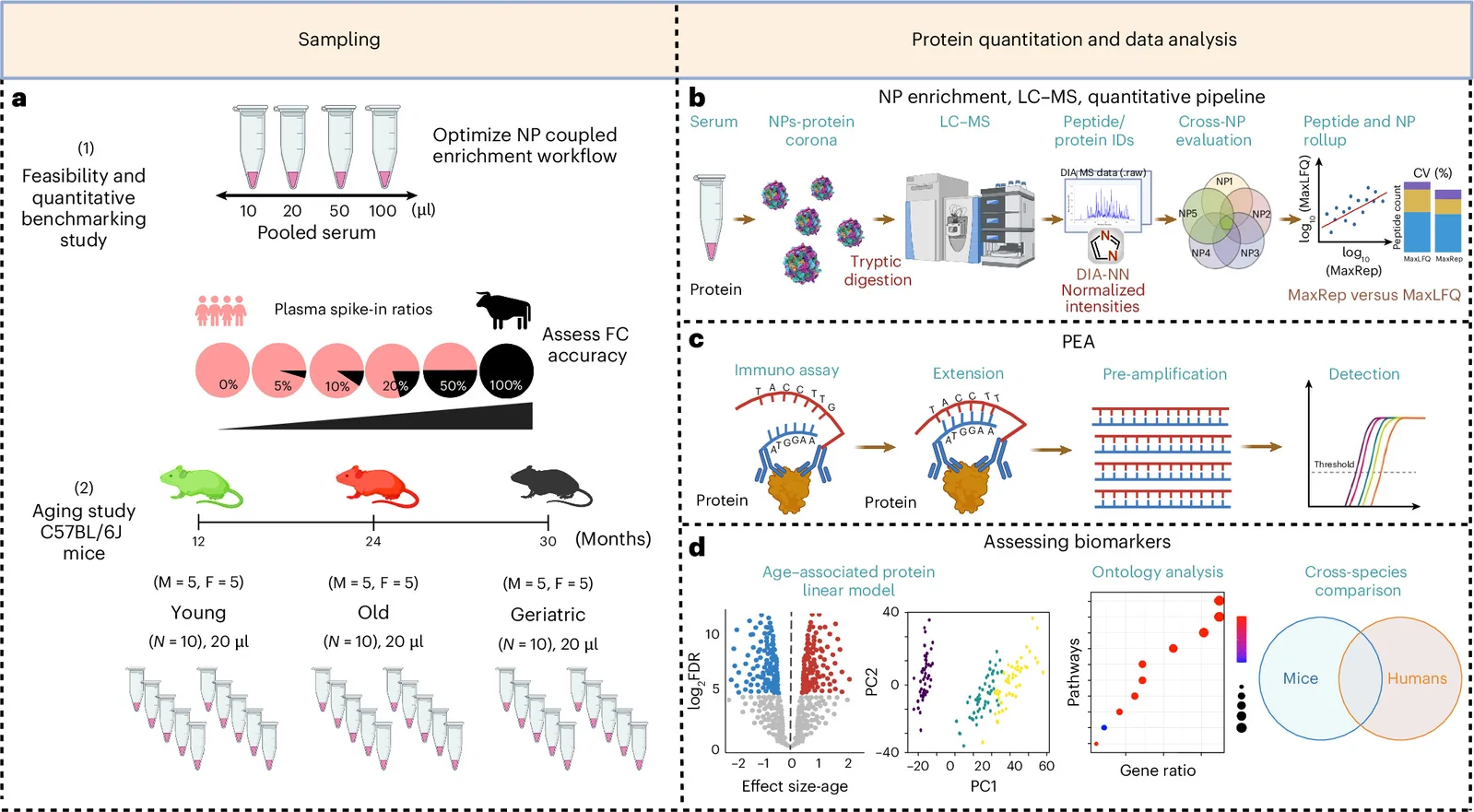
Overview of study design for a comprehensive and quantitative proteomic study in mouse serum. **a**, Schematic summary of the study design with two phases: (1) a feasibility and quantitative benchmarking study and (2) an aging study. In phase 1, to initially assess the feasibility of this workflow at low volumes, optimized NP enrichment and the LC–MS workflow with pooled mouse serum was conducted with four different volumes (10, 20, 50 and 100 µl) and compared to traditional neat serum digest (no enrichment) processed in parallel. To assess quantitative accuracy, a benchmarking study was conducted on defined ratios plasma from two organisms (bovine and human). In phase 2, to demonstrate the power of the workflow in a biological study, an aging study of 30 mice was conducted at three ages: 12 (young), 24 (old) and 30 (geriatric) months. M, male; F, female. **b**, The automated serum-NP processing and digestion were performed using a Proteograph (Seer) workflow. Peptide data was acquired by nano LC–MS/MS and analyzed with DIA-NN in a library-free mode. NP-peptide enrichment was assessed by comparing two generic label-free quantification algorithms, MaxRep and MaxLFQ. **c**, A panel of 48 mouse cytokines were measured using PEA (Olink, Thermo Scientific). **d**, Biomarkers and pathways associated with aging and cross-species comparison were assessed. Figure created in BioRender; Dey, A. K. https://biorender.com/jqszyrc (2026).

**Extended Data Fig. 1:**
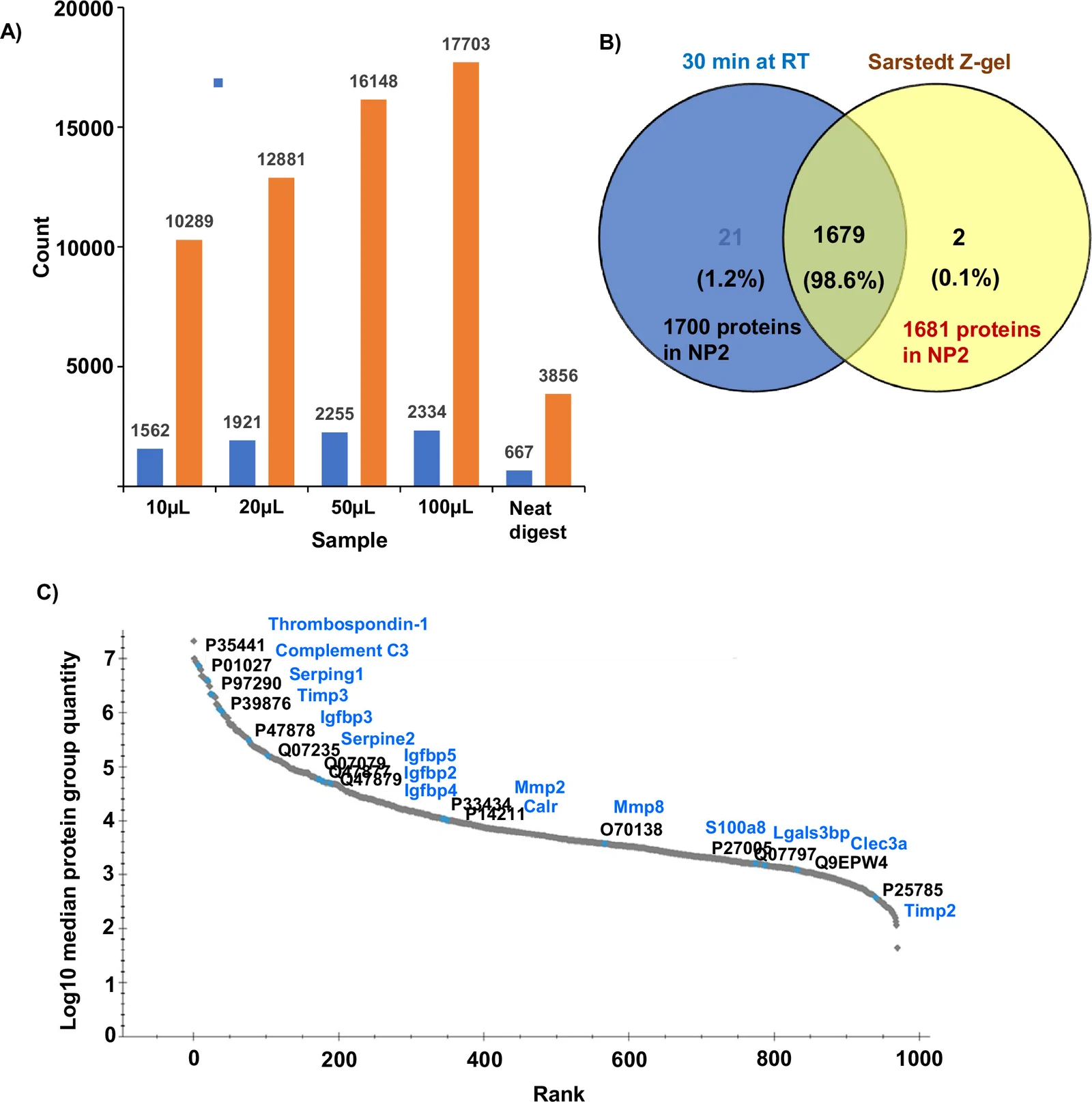
Feasibility study. **a)** Protein groups and peptide counts identified at each sample volume in feasibility study (pooled serum). For the 20 µl sample, the bars represent the mean of three replicate measurements for protein groups and peptides, respectively; all other sample were analyzed once. (**b)** Venn-diagram between two serum preparation methods. **c)** Protein ranked plot showing dynamic range of protein distribution. Source data

We developed a multicomponent study designed to establish a quantitative serum proteomics method compatible with low-volume mouse serum, and to validate its accuracy and ability to identify circulating aging biomarkers (Fig. 1a). A combination of automated NP enrichment coupled to DIA-MS (Fig. 1b) with an affinity cytokine assay (Fig. 1c) was leveraged to detect and quantify serum proteins for an assessment of aging biomarkers and cross-species comparison (Fig. 1d). In a feasibility study using pooled mouse serum (10–100 µl), NP processing increased protein depth 2–4-fold compared to non-volume-limited neat digests (Extended Data Fig. 1a), consistent with previous observations indicating that NPs mediated dynamic range compression^16^. Serum collection method had minimal impact; clot-derived (for 30 min at room temperature) serum and Sarstedt Z-gel tube serum yielded 1,700 and 1,681 protein groups, respectively, of which over 90% were shared (Extended Data Fig. 1b). For all downstream analysis, clot-derived serum was used.

**Fig. 2:**
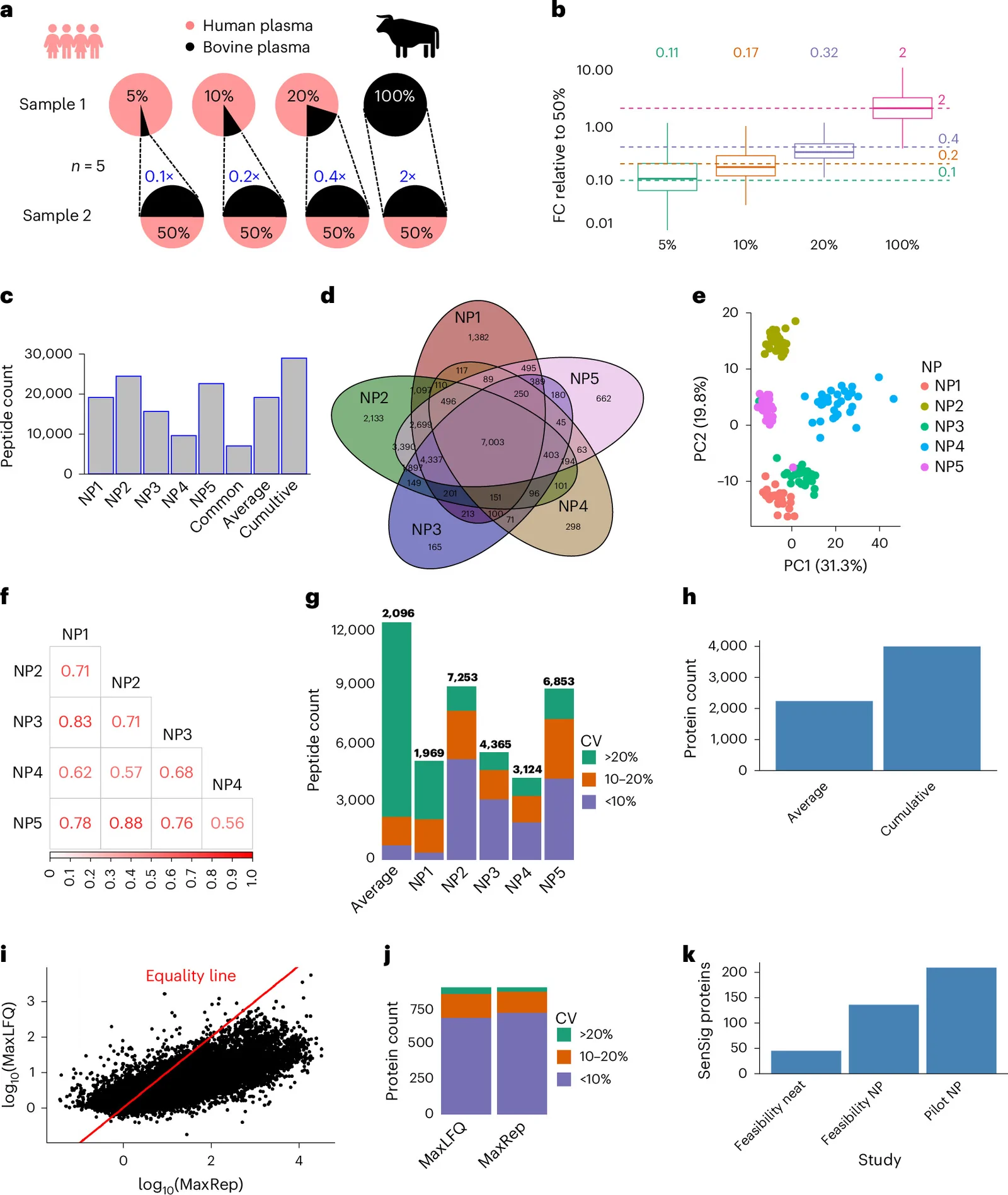
Qualitative and quantitative evaluation of proteomic data generated NP-coupled MS workflow. **a**, A quantitative benchmarking study was performed using five fixed ratios of human and bovine plasma (*n* = 5 each). **b**, Peptide abundance ratios in each dilution versus the 50% bovine plasma sample. Box plots are median centered (solid horizontal line inside the box) and segmented by quartiles (25th and 75th percentiles). Dotted lines depict the expected ratios. **c**, Peptides identified in at least one sample by NP. **d**, Proteins quantified in at least one sample are shown for each NP. **e**, PCA of 5 NP in 30 mice was used to show clustering patterns between NPs on the peptide level. **f**, Spearman correlation values for peptide intensities between NP. **g**, The CV (s.d /mean abundances) is shown by NP on the peptide level for three triplicate samples. For each NP, only those peptides detected in all three samples were used for calculation. For combined, intensities were added between all NP and proteins detected in all three samples (regardless of which NP) were kept for analysis. Numbers above each bar reflect the number of peptides that show <20% CV. **h**, Protein identifications in mouse aging study. **i**, Correlation of protein intensities calculated by the MaxRep versus MaxLFQ approaches (*n* = 30) (correlation of 0.77, *P* value <0.00001). **j**, Protein CVs in the MaxRep and MaxLFQ quantitation pipelines for three triplicates samples. **k**, Number of SenSig proteins detected in neat serum and serum processed by the NP workflow. Panel **a** created in BioRender; Dey, A. K. https://biorender.com/jqszyrc (2026). Source data

To evaluate biological relevance of the proteomic results in the context of aging, we assessed coverage of known aging-associated biomarkers and processes. Cellular senescence is a widely accepted hallmark of aging, and senescence associated circulating proteins are clinically relevant biomarkers^3,4,7^. In the feasibility study, we detected multiple canonical senescence-associated secretory proteins (SASPs) spanning the dynamic range, including Igfbp’s 2/3/4/5, Timp’s 2/3, Thbs1 and Mmp’s 2/8, among other known markers (Extended Data Fig. 1c). For a more comprehensive assessment in the aging study, we evaluated the detection of a recently described in vivo mouse senescence signature, SenSig, in serum^26^. Compared to neat serum, where 45 SenSig proteins were identified, 136 and 209 SenSig proteins were identified in NP processed samples or across all samples in the aging study (Fig. 2h), respectively, demonstrating a substantial increases the detection of biologically relevant low-abundance proteins.

A measurement is quantitative when the relative change in the measured signal reflects the change of the peptide quantity in the original samples. Given the known effect of the NP-coupled MS workflow on the dynamic range of proteins in a sample, it is critical to determine whether the samples produced by this workflow preserve the quantitative differences in the original samples. To evaluate the ability of the NP-processing workflow to accurately quantify peptide abundance, we conducted a benchmarking study similar to previously described matrix-matched calibration curves and LFQ-Bench studies^27,28^. In brief, we produced benchmarking samples containing known ratios of human and bovine plasma (Fig. 2a), including 19:1 (5% bovine), 9:1 (10% bovine), 4:1 (20% bovine), 1:1 (50% bovine) and 0:1 (100% bovine) ratios (*n* = 5 each). Each sample/dilution was processed through the standard NP workflow. In this way, the accuracy of the NP workflow can be assessed by comparing reported peptide abundances between samples to known ratios. The fold change (FC) in peptide intensities was calculated for all dilutions relative to the 50% bovine samples. The measured peptide ratios were similar to the expected ratios (Fig. 2b), with the median of the 5% and 100% bovine samples being within 0.01 of the expected ratios and the expected ratios of the 10% and 20% bovine sample groups within their respective interquartile range. These findings suggest that the NP workflow accurately depicts the relative changes of the original serum samples.

### Evaluation and optimization of NP-coupled MS in a low-volume mouse serum study of aging

To evaluate the performance of the NP-coupled MS workflows in identifying circulating biomarkers, we applied this method to serum in a cross-sectional subset of 30 C57BL/6J mice from SLAM at 12, 24 and 30 months (*n* = 10 per age; 50% female), using 20 µl per mouse (Fig. 1a, ‘Aging study’). Following NP capture and trypsin/Lys-C digestion, peptides were analyzed by DIA-MS and processed using a DIA-NN database search algorithm within the Proteograph Analysis Suite.

**Extended Data Fig. 2:**
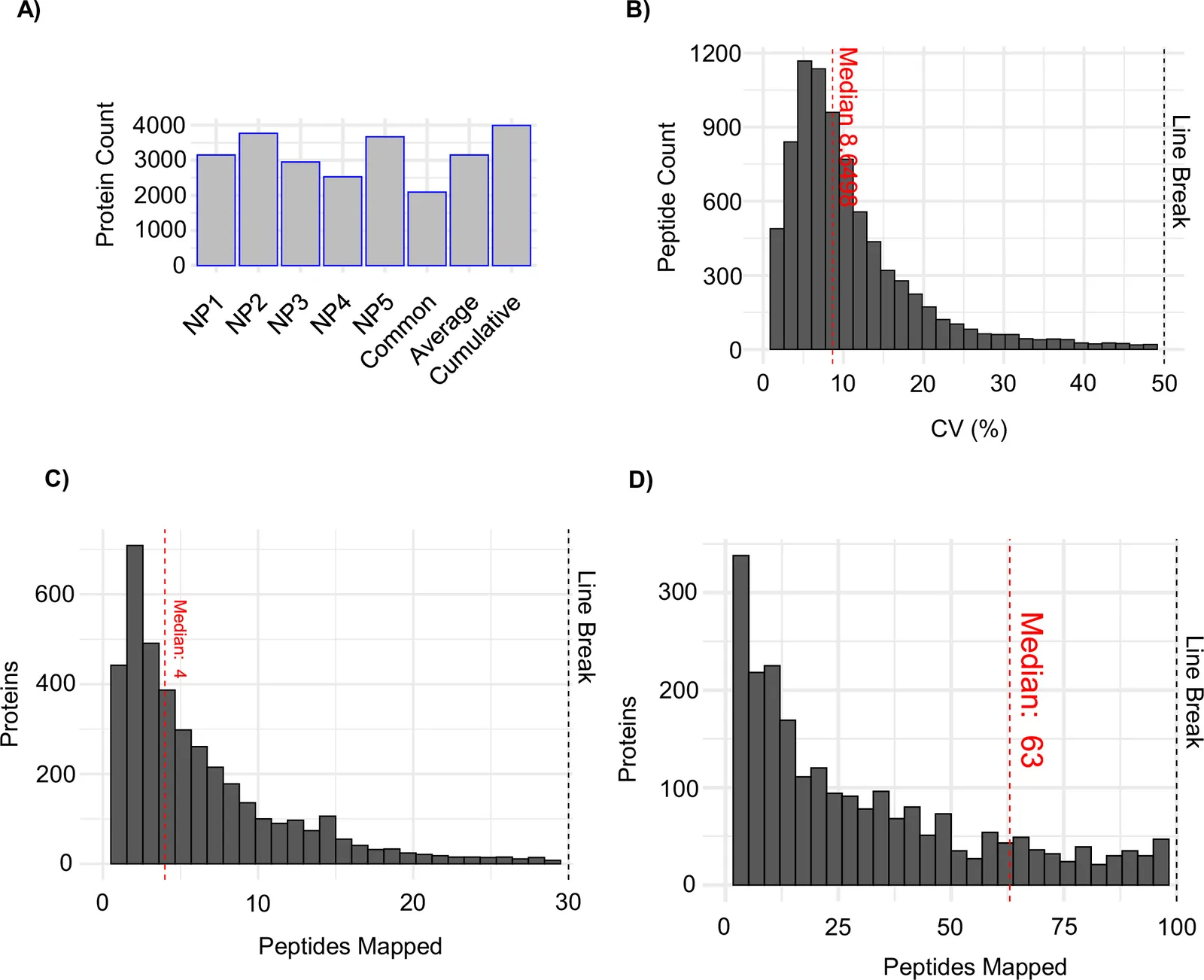
Comparison of Nanoparticle Rollup and Quantification. **a)** The number of proteins identified by NP in the aging study (N = 30). **b)** Coefficient of variation (CV) (Standard Deviation /Mean abundances * 100) is shown for NP2 on the peptide level for 3 triplicate samples. Only those peptides detected in all 3 samples were used for calculation. For clarity, a line break at 50% is used. **c)** The number of peptides mapped to each protein in the aging study (N = 30). For clarity, a line break at 30 is used. **d)** The number of unique peptide observations mapped to each protein in the aging study (N = 30), each NP counted separately. For clarity, a line break at 100 is used. Source data

Across all 30 mice, approximately 10,000–25,000 peptides were detected per NP fraction, and 28,976 unique peptides were detected cumulatively (Fig. 2c). In rank order of the number of peptides identified, NP2 and NP5 detected the largest peptide sets (24,457 and 22,592 peptides, respectively), followed by NP1 (19,129) and NP3 (15,650), with the fewest peptides in NP4 (9,587). A similar trend was observed for protein counts across the NPs (Extended Data Fig. 2a). A shared core of 7,003 peptides was commonly detected across all NPs, and 1,382, 2,133, 165, 298 and 662 peptides were uniquely identified, respectively, in NPs 1–5 (Fig. 2d). Principal component analysis (PCA) revealed clustering of NP by peptide expression levels (Fig. 2e). Among peptides quantified in all NPs, moderate to high correlations in peptide expression levels by NP were observed, such as between NP1 and NP3 (rho 0.83) and NP2 and NP5 (rho 0.88) (Fig. 2f). Total peptide counts and the coefficient of variation (CV) from technical triplicates with 20-µl serum volume showed that NP2 and NP5 exhibited the highest peptide detections, corroborating the NP ranks observed in Fig. 2c (Fig. 2g). However, NP2 demonstrated the greatest precision in peptide identification with a median CV of 9% (Extended Data Fig. 2b), with over half of the total identified peptides having a CV of less than 10%. As expected, the CVs of the cross-NP average are the highest, consistent with differences in the relative affinity of each NP for any given peptide. Overall, this contrast between NPs is consistent with previous studies^15,29^, suggesting that distinct NPs provide heterogeneous and reproducible surface chemistries that facilitate the formation of distinct corona compositions.

### Comparing rollup approaches for protein-level quantification from NP fractions

To develop an optimal pipeline for the quantitation of proteins with independent peptide measurements across distinct NP fractions, we compared MaxRep and MaxLFQ rollup approaches. In MaxRep, for any given peptide that is observed across multiple NPs, the measured value used is taken from the NP with greatest observed intensity across all samples. MaxLFQ, a previously described peptide rollup approach^30^, aggregates peptide intensities across both the NP and peptide level for all observations, as previously reported on this platform^29^. Using either method, a total of 3,992 protein groups were quantified cumulatively (2,235 on average; Fig. 2h), with a median of 4 peptides mapped to each protein (Extended Data Fig. 2c) and a median of 63 distinct peptide-NP measurements observed per protein (Extended Data Fig. 2d). MaxRep and MaxLFQ methods were significantly correlated (correlation 0.77, *P* value <0.00001), although the correlation is weakest among high intensity observations (Fig. 2i). To assess the precision of each protein quantitation workflow, we analyzed proteins quantified by five NPs across technical triplicates of 20-µl serum samples. The median protein-level CVs were 0.0567 and 0.0613 for MaxRep and MaxLFQ, respectively, and the proportion of proteins below 10% CV was modestly higher in MaxRep (Fig. 2j). Accordingly, MaxRep was used for primary analyses due to its marginally improved reproducibility, while downstream biological conclusions were robust to rollup choice. Overall, NP processing increased protein quantification compared to the neat samples, irrespective of data processing algorithms or NP fraction, consistent with dynamic range compression in NP coronas.

### Identification of cytokines commonly missed by MS

**Fig. 3:**
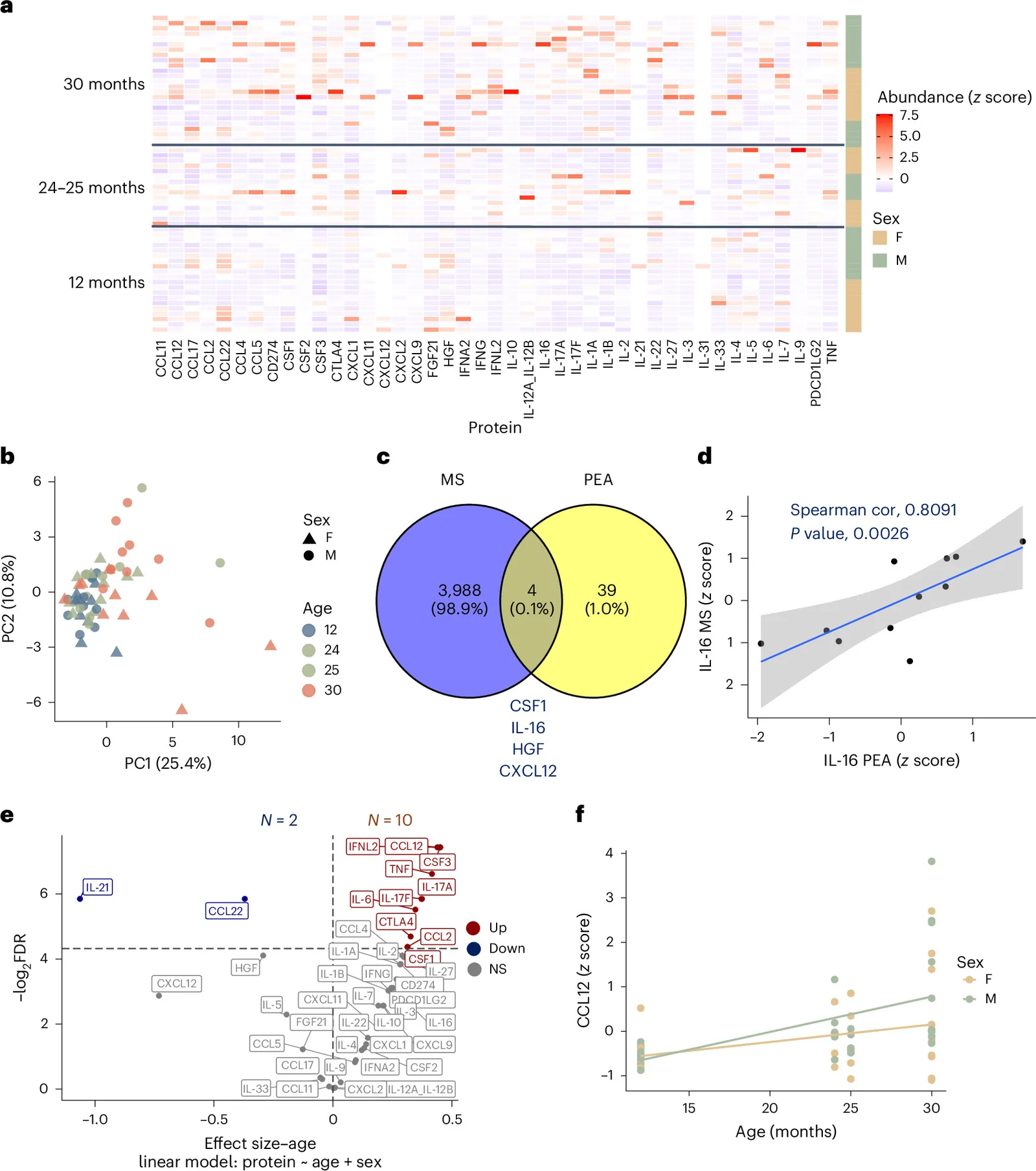
Age-related proteomic changes measured with PEAs. **a**, Heat map of scaled protein abundance for all proteins measured with PEA in the aging study (*n* = 60 mice). **b**, PCA of protein abundance by age and sex (*N* = 60). **c**, Proteins detected using both NP-MS and PEA methods (*N* = 30). **d**, Two-sided Spearman correlation (cor) of IL-16 levels measured by NP-MS and PEA (*N* = 30). The shaded area denotes the 95% confidence interval (CI). **e**, Pearson modeling revealing age-associated proteins measured by PEA using the formula protein ~ age + sex (*N* = 60). **f**, IFNl2 levels by age in males and females (*N* = 60). NS, not significant. Source data

While a large number of proteins, including inflammation and senescence-associated proteins (Fig. 2k and Extended Data Fig. 1c), were detected following the NP-MS workflow, prototypical inflammatory cytokines, such as interleukin-6 (IL-6), were notably not detected, consistent with previous MS studies^4,31^. Given the important role of cytokines in a range of diseases and aging, including as a key bioactive component of the SASP, we sought to complement MS with minimal additional serum. We profiled cytokines using a targeted 48-plex Olink PEA, quantifying 43 inflammatory cytokines from 1 µl of additional serum per mouse (Fig. 3a and Supplementary Table 2). While heterogeneity can be seen between biological replicates at any age, a general increase in abundance of proteins across the panel with age is apparent (Fig. 3a), and age and sex-dependent separation is apparent by PCA (Fig. 3b). Notably, only four proteins were detected by both MS and the cytokine panel (Fig. 3c), demonstrating the complementarity of this approach to detect biologically important cytokines that are missing by MS. To assess the agreement between the MS and antibody-based assays, we compared the data for IL-16, which contained sufficient data points in both datasets for correlation. IL-16 quantification was significantly correlated between the two approaches (*P* = 0.0085, correlation 0.7455; Fig. 3d), confirming the MS-based quantitation of this protein.

To identify linearly age-dependent cytokine changes in circulation, linear modeling was performed adjusting for sex as a covariate (protein ~ age + sex). Notably, ten cytokines significantly linearly increased with age, and two cytokines, IL-21 and C-C motif chemokine ligand 22 (CCL22), decreased with age (Fig. 3e). CCL12 was the most significant change (Fig. 3f). Overall, these results highlight the complementarity of this approach to identify biologically relevant cytokine changes in 1 µl of serum.

### Age-associated changes in the serum proteome

**Fig. 4:**
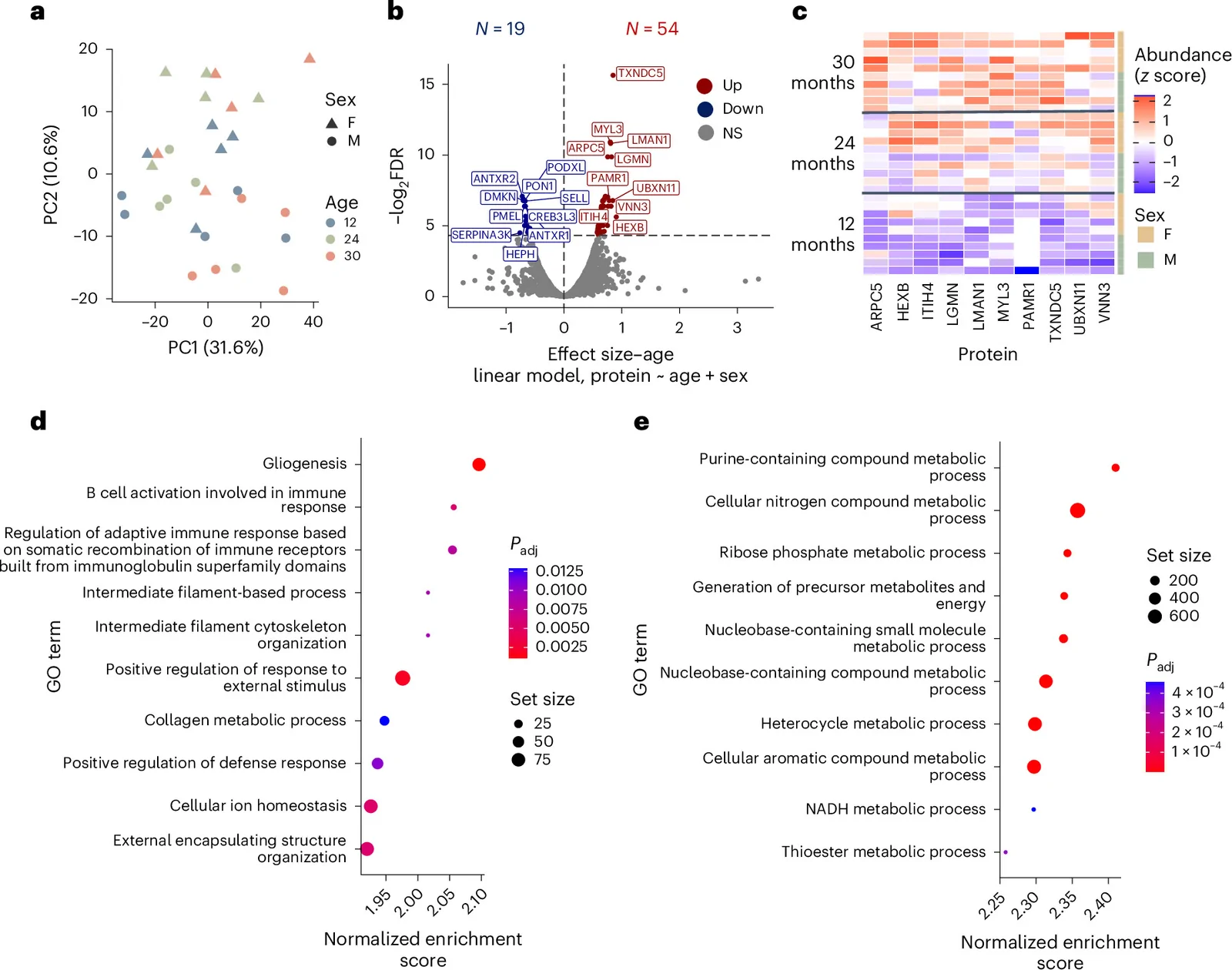
Age-associated changes in circulating proteins measured by NP-MS. **a**, PCA of quantified proteins by age and sex. **b**, Pearson linear modeling to identify age-associated proteins, using the model protein ~ age + sex. Only proteins present in at least one sample from each age and sex group and with at least 2 peptides were examined (*N* = 2,662). **c**, Scaled abundance of the top 10 linearly increasing age-associated proteins. **d**, Gene set enrichment analysis of protein abundance (log_2_FC) changes from ages 12 to 24 months (Biological Process). **e**, Gene set enrichment analysis of protein abundance (log_2_FC) changes from ages 24 to 30 months (Biological Process). For both **d** and **e**, multiple test correction was performed using the Benjamini–Hochberg method. Source data

For aging-related analyses, we focused on proteins that had ≥2 mapped peptides (*N* = 2,662) and were present in at least three replicates per age group and both sexes. All numerical protein data associated with the MS analysis are available in Supplementary Table 3. Protein-level PCA clustered samples by sex and modestly by age (Fig. 4a). Linear modeling (protein ~ age + ex; false discovery rate (FDR) ≤0.05) identified 73 age-associated proteins (54 increased and 19 decreased; Fig. 4b). Thioredoxin Domain-Containing Protein 5 (TXNDC5), Myosin light chain 3 (MYL13), Actin Related Protein 2/3 Complex, Subunit 5 (ARPC5), Legumain (LGMN) and Lectin Mannose Binding 1 (LMAN1) were among the strongest positive age-associated changes (Fig. 4c). Gene set enrichment analysis revealed gilogenesis and immunity-related pathways enriched from 12 to 24 months of age (Fig. 4d), while from 24 to 30 months metabolic processes were enriched (Fig. 4e). Collectively, these results reveal robust linear age-associated changes that occur in the circulating proteome and suggest additional stage-specific changes in subsets of proteins in old age versus geriatric age.

### Sexual dimorphism of age-related changes in circulating proteins

**Fig. 5:**
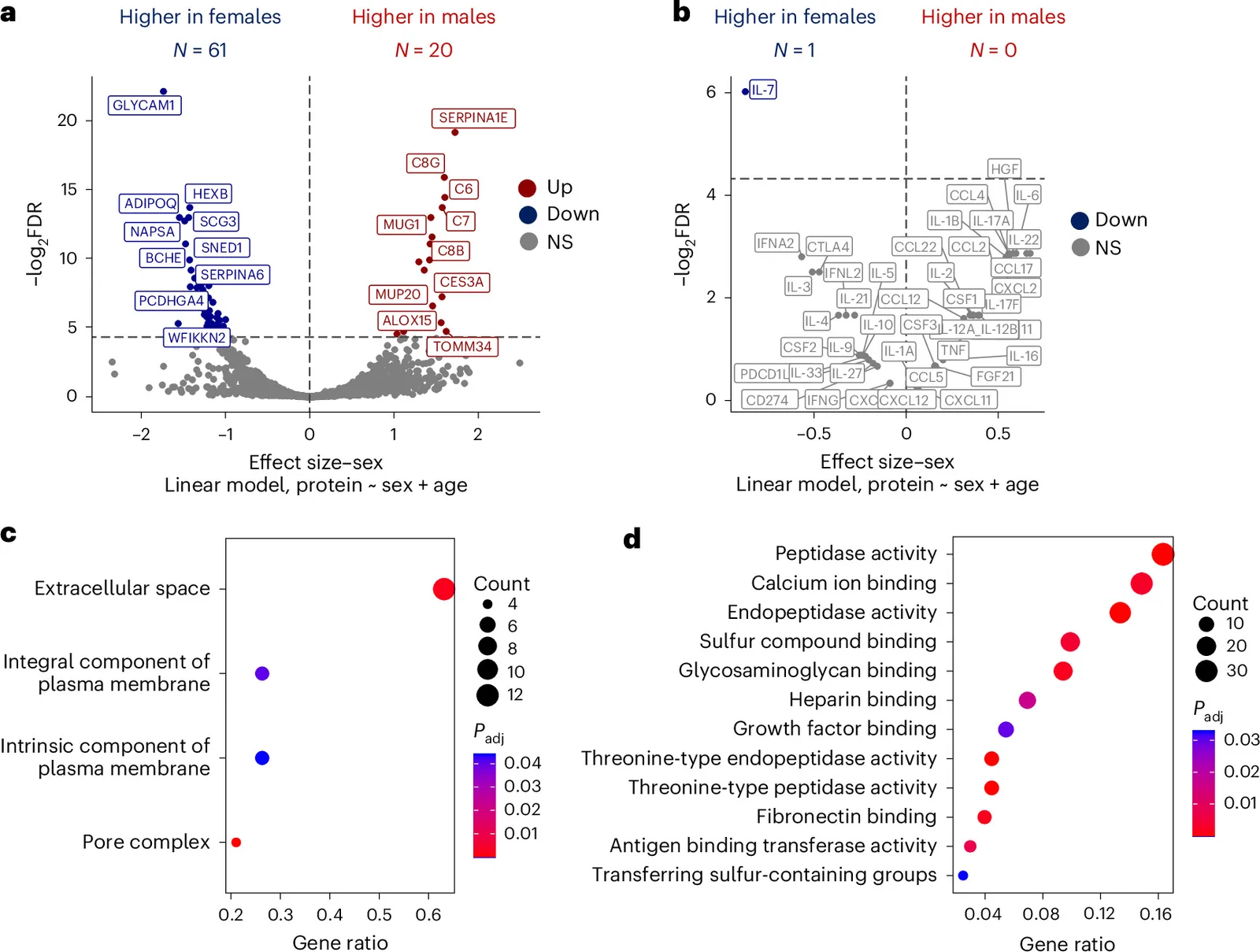
Circulating protein associated with sex. **a**, Pearson linear modeling to identify sex-associated proteins measured by the NP-MS workflow, using the model protein ~ sex + age. **b**, Pearson linear modeling to identify sex-associated proteins measured by the PEA workflow, using the model protein ~ sex + age. **c**, Overrepresentation analysis of proteins upregulated in males (Cellular Component). **d**, Overrepresentation analysis of proteins upregulated in females (Molecular Function). For both **c** and **d**, a multiple test correction was performed using the Benjamini–Hochberg method. Source data

Next, sex-associated differences were assessed by Pearson linear modeling (protein ~ sex + age) in the 30 mice from the aging cohort, identifying 20 proteins elevated in males and 61 proteins in females by NP-MS (Fig. 5a). In PEA cytokines (cytokine ~ sex + age), only one protein (IL-7) was higher in females (Fig. 5b). Overrepresentation analysis of NP-MS-identified proteins indicated male-elevated proteins were enriched for plasma membrane (Fig. 5c), whereas female-elevated proteins were enriched with peptidase activity, among other functions (Fig. 5d). These findings reveal sex dimorphism in the circulating proteome and demonstrate that this workflow is sensitive enough to sufficiently capture sex differences in the circulating proteome.

### Mouse age-associated circulating proteins with comparison to human aging

**Fig. 6:**
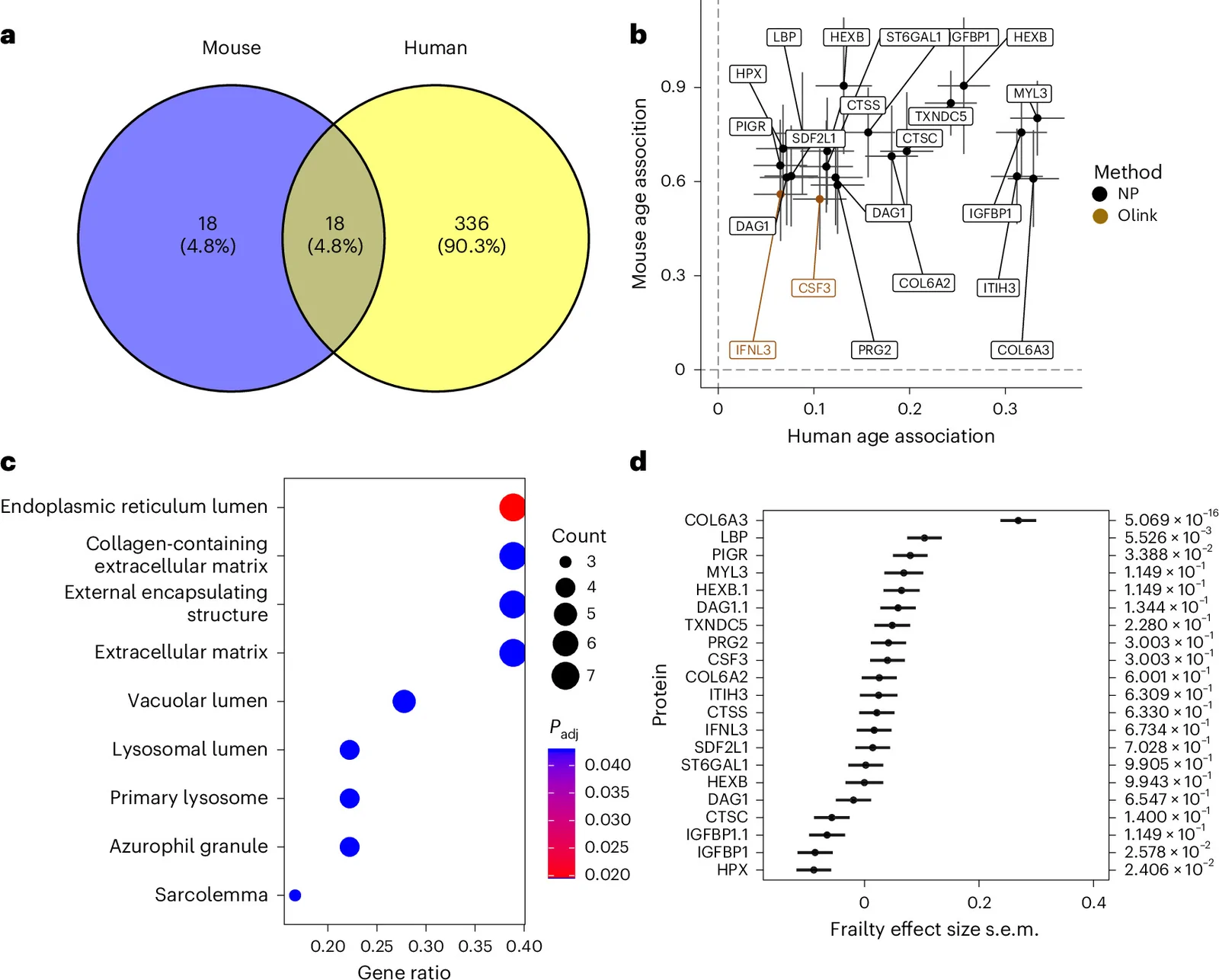
Circulating proteins associated with age and frailty in mice and humans. **a**, Venn diagram illustrating proteins identified as positively age-associated using Pearson linear modeling across the NP-MS workflow and the Olink platforms in the cohort analyzed in this study (left, blue circle, *N* = 30 mice), and proteomic measurements (SomaScan 7k assay) in the BLSA (right, yellow circle, *N* = 1,275 participants), modeled as age ~ protein + sex (total proteins quantified across both datasets, *N* = 1,742). **b**, Proteins significantly associated with age in mice and humans, determined using Pearson linear modeling (age ~ protein + sex). Color coding indicates the detection method in this study. Error bar indicates standard error. **c**, Overrepresentation analysis of proteins significantly upregulated with age in both species, annotated by cellular component. Multiple test correction performed using the Benjamini–Hochberg method. **d**, Associations between circulating proteins and frailty assessed using Pearson linear modeling across the cohort analyzed in this study and the BLSA, based on a 44-component frailty index and the model frailty index ~ protein + age + sex. Error bar indicates standard error. *P* values are from multiple tests corrected by the Benjamini–Hochberg method. Source data

To assess cross-species concordance, we compared age-associated circulating proteins in mice with human plasma proteomics from the BLSA^7,32^. Among the 1,742 proteins quantified in both datasets, Pearson linear modeling (age ~protein + sex) identified 18 proteins, representing 5% of the total proteins or 50% of mouse age-associated proteins, to be significantly (Fig. 6a) and positively (Fig. 6b) associated with age in both mice and humans. Overrepresentation analysis of shared upregulated proteins with age in both mice and humans revealed enrichment for cellular component categories (Fig. 6c) including endoplasmic reticulum, extracellular matrix and lysosomal proteins, among others, and suggesting shared categories of age-associated circulating factors at a pathway level. To assess the relevance of these findings with age-associated clinical phenotypes, we evaluated the associations of these proteins with frailty index, a 44-component composite score, in the BLSA. Notably, five proteins including COL6A3, LBP, PIGR, IGFBP1 and HPX were significantly associated with frailty index in the BLSA (frailty index ~ protein + age + sex; Fig. 6d). Collectively, these results link conserved proteomic aging signatures to functional health decline in humans and suggest that aging-associated biomarkers derived from mice may be relevant in humans.

## Discussion

Currently, there is a lack of tools available to comprehensively detect and quantify proteins in the low volumes of blood available from mouse studies, which generally cannot accommodate existing approaches such as extensive fractionation^33^. As a result, biomarker studies in mouse cohorts are sparse and unfeasible to most researchers. Furthermore, even with extensive processing, MS-based proteomic studies notably miss detection of a large portion of cytokines, a highly sought after subset of protein biomarkers with biological relevance across multiple domains of health and disease research. In this study, we present a scalable, NP- based DIA-MS workflow that enables deep and quantitative profiling of mouse serum from a volume as low as 20 µl, with an additional 1 µl used for targeted cytokine measurement. To our knowledge, this represents the most comprehensive survey of the mouse serum proteome achieved so far in low sample volumes, enabling biomarker research that includes key biologically relevant proteins such as cytokines in experimental designs with volume limitations, such as longitudinal studies or multicomponent serum studies.

A major technical challenge in serum proteomics is the wide dynamic range of protein concentrations, spanning 10–12 orders of magnitude^12^. An automated and unbiased NP-based MS workflow offers a solution^15,17,18,29^ by compressing dynamic range. By leveraging a panel of physicochemically distinct NPs, this workflow forms a ‘protein-corona’ at the nano–bio interface that enriches low-abundance proteins typically masked in neat digests. Consistent with prior human biomarker studies^15–18,29,34,35^, our results demonstrate that NP enrichment substantially increases proteome depth while maintaining quantitative fidelity. Benchmarking experiments using defined human–bovine plasma mixtures confirmed that NP processing preserves relative abundance relationships and accurately reflects expected FCs, supporting its suitability for quantitative analyses.

Concerns have been raised that NP coronas may be sensitive to nonprotein serum components, such as lipids or metabolites, potentially introducing variability unrelated to biological differences. While recent work has shown that small molecules can influence protein corona composition derived from plain polystyrene NPs^36^, multiple independent studies of the surface-functionalized superparamagnetic iron NPs used in this study suggest that these effects are not influential. Lipid spike-in experiments using the same NPs showed no appreciable changes in corona protein identities or intensities^15^. Similarly, a prior report suggests minimal impact of background matrix on quantitative accuracy^37^. Our own quantitative benchmarking data further support this conclusion, showing high reproducibility and accurate quantification of known FCs in samples generated from mixing human and bovine blood, each containing a distinct composition of lipids, metabolites and other components, in a range of fixed dilutions spanning 5% to 100% (Fig. 2b). Together, these findings indicate that matrix-derived components did not considerably alter corona composition in a manner that distorts relative protein quantification in this study and mitigates concerns that matrix effects introduced variability unrelated to aging. The recent development of a high-throughput NP-protein corona enrichment in serum and plasma in the context of a mouse cancer model^38^ underscores the growing utility of the strategy.

Our study integrates a five-NP workflow with DIA-MS, to demonstrate the feasibility of adapting this approach to low-volume mouse studies, identifying almost 3,992 proteins and 28,976 peptides from 20 µl of serum in a 30-mouse cohort, a sixfold improvement over the standard neat workflow. We also optimized a reproducible and quantitative pipeline for the analysis of protein-level changes from NP-fractionated peptide data. Although the greatest proteomic depth is achieved by combining five NP fractions, if fewer fractions are desired, the majority of the improvement in proteomic depth (>5-fold increase over neat samples) can be achieved by using only NP2 (Extended Data Fig. 2d). Recently, two-NP kits have also shown comparable proteomic depth to five NPs^7,39,40^. Application of this workflow to young, old and geriatric mice revealed extensive age-, sex- and stage-specific changes in the circulating proteome. However, because the proteomic data derive from a cross-sectional subset of the longitudinal SLAM cohort, age-associated signatures reflect population-level differences rather than within-individual aging trajectories. All biological interpretations are therefore framed conservatively, and the results should be viewed as foundational and hypothesis-generating rather than mechanistic or causal.

The NP-based workflow greatly enhanced the detection of biologically relevant proteins and candidate biomarkers. Cellular senescence and inflammation are key hallmarks of aging that have previously been associated with aging and various age-associated clinical phenotypes in humans^31,41^. Compared to a standard workflow, which detects <50 senescence-associated markers described by SenSig^26^, the NP workflow identified >200 SenSig proteins. The NP workflow also enabled the detection of low-abundance inflammatory proteins, such as CXCL12 and IL-16. Despite these improvements, the MS-based workflow notably missed many widely studied cytokines often measured in biomarker studies, such as IL-6, a widely used clinical indicator of inflammation and a prototypical senescence-associated secreted protein^4^. Cytokines often go undetected in MS studies because they are at the low end of the dynamic range of blood and are generally small proteins with a small number of unique tryptic peptides that ionize well^20,21^. To address this gap, we integrated a targeted 48-plex PEA^42^ quantifying 43 cytokines undetected by MS, including IL-6 in only 1 µl of additional serum. Standard antibody-based cytokine arrays would presumably be complementary as well, although the sensitivity and volume requirements of other traditional technologies would differ. Overlap between MS and PEA was limited (four proteins; Fig. 3c), reflecting the fundamentally different detection principles and sensitivity ranges of the two platforms. However, cytokines uniquely detected by PEA exhibited coherent age-associated patterns including increases in ten cytokines, including known SASP factors IL-6, TNF, CSF3, CSF1 and CCL2. Together with the observed increases in SenSig proteins and immune cell activation pathways by MS-based proteomics, these results support an age-related increase in senescence-driven immune cell activation in circulation. The PEA panel provides an independent assessment of the inflammatory state of the organism based on inflammatory cytokines that are missed by traditional unbiased MS assays.

Enabled by this workflow, we identified age- and sex-dependent features of the serum proteome in a cross-sectional subset of the SLAM, a deeply phenotyped cohort of normative aging that includes multiomics^11^. Given the limited blood volumes available in studies of this scale, in addition to the need to portion for multiple assays, it was critical that comprehensive proteomic profiling and statistically significant associations with biological phenotypes could be achieved within a 25-µl serum limit. Indeed, the workflow revealed clear biological determinants of serum proteome composition, including age and sex. We found that Myosin light chain 3 (MYL3), a core muscle component known to increase in the serum during aging and muscle wasting^43,44^, was among the strongest linearly age-associated proteins. Interestingly, one of the most negatively associated aging proteins, Paraoxonase 1 (PON1), is an atheroprotective protein with a known age-associated decrease, and variants in this gene have been associated with longevity^45^. These findings underscore the sensitivity and robustness of this workflow to detect significance in aging biology and suggest the value of inclusion of multiple time points and longitudinal study designs in future studies. This automated workflow is readily scalable to deeper longitudinal investigations including more mice and time points.

**Extended Data Fig. 3:**
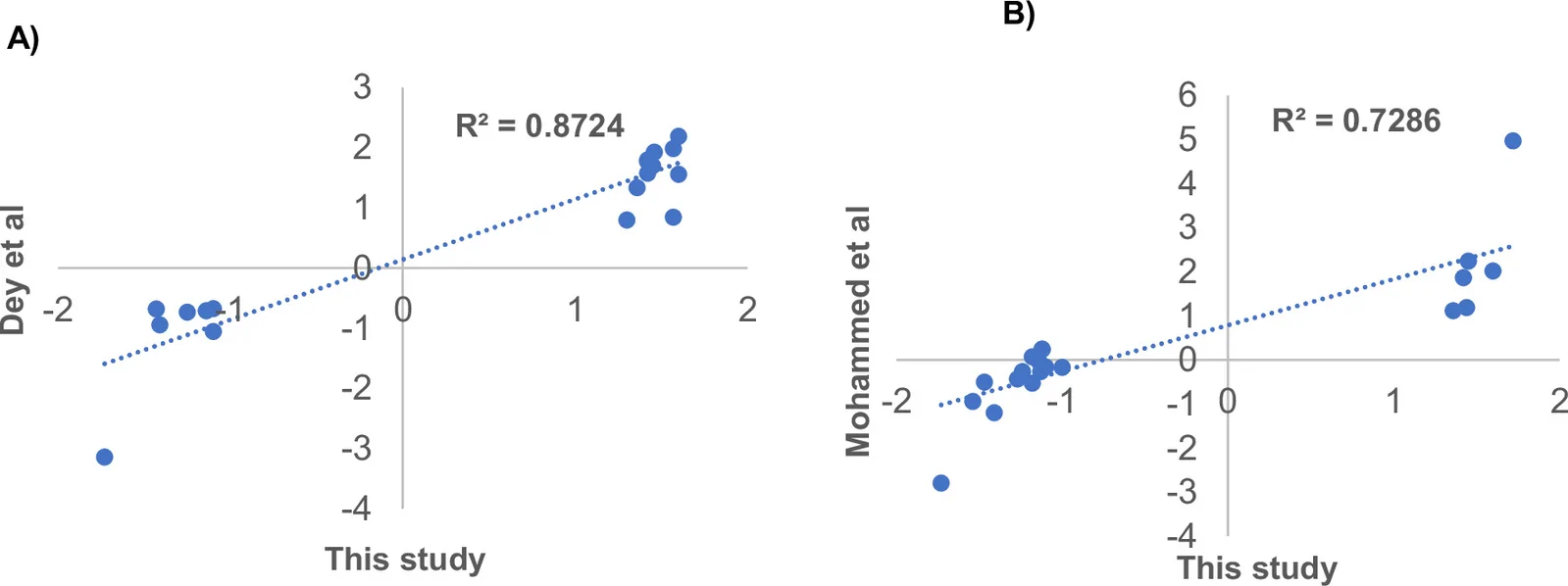
Correlation of Sex-Associated Proteins. Correlation plots comparing sex-associated changes observed in our study with those reported by **a)** Dey et. al. (PMID: 37910662); and **b)** Mohammed et. al. (PMID- 34050187). Proteins in both studies show strong positive correlations with our study (R^2^ = 0.8724 and R^2^ = 0.7286, respectively), and consistent directionality of sex associated effects. Source data

Few studies existed to draw comparisons to and for the validation of our findings. While similar age-focused studies are lacking, prior mouse proteomics analyses—including an extensively fractionated (3,972 serum proteins in 128 fractions) Alzheimer disease model^33^ and a targeted MS survey (375 plasma proteins) across 30 knockout strains^46^—reported numerous sex-associated proteins with which to compare our data. Remarkably, except 2 proteins (Transcobalamin-2 and Hepatocyte growth factor activator) out of 32, all sex-associated proteins overlapping with our study exhibited the same directionality of sex associations^33,46^. (Supplementary Table 4 and Extended Data Fig. 3a,b). Several of these proteins, including complement components C8g, C6, C7, C8b and C8a; lipocalins such as major urinary proteins MUP20 and MUP3; serine protease inhibitors such as Serpina1e and Serpina3k; Cysteine-rich secretory protein 1 (Crisp1); and the transmembrane glycoprotein epidermal growth factor receptor (EGFR), were elevated in male mice in other studies^47–53^. In females, higher levels of proteins such as glycosylation-dependent cell adhesion molecule 1 (GLYCAM1), adiponectin (ADIPOQ), cholinesterase (BCHE) and SERPINA6 were reported in other studies^54–57^. Pathway analysis similarly revealed sex-specific signatures in circulation, with membrane protein pathways in males (Fig. 5c), and peptidase and glycosaminoglycan-related pathways enriched in females (Fig. 5d). Altogether, sexual dimorphism emerged as a prominent feature of the circulating proteome, with both NP-MS and targeted cytokines analyses identifying several sex-associated proteins, and the majority of them showed similar sexual directionality consistent with prior mouse proteomics studies^33,46^. These findings underscore sexual dimorphism in blood proteins, reinforce the necessity of including both sexes in aging studies and support the accuracy and robustness of the NP-MS workflow through strong agreement with the existing literature.

To place the mouse findings in a translational context, we compared age-associated circulating proteins identified here with human plasma proteomics from the BLSA. Despite differences in species, cohort design and analytical platforms, a subset of proteins, representing half of the mouse age-associated proteins, were concordantly age-associated in humans (Fig. 6a,b), indicating conserved features of the circulating aging proteome. Interestingly, collagen-containing extracellular matrix proteins were overrepresented among these, consistent with recent reports of the association of extracellular matrix components, including collagen, with aging in mouse circulation^58^. Moreover, several of these shared proteins, including COL6A3, LBP, PIGR, IGFBP1 and HPX, were also significantly associated with frailty in humans, linking shared circulating proteomic signatures to functional health decline. Notable among these, collagen type VI alpha 3 chain (COL6A3, also known as endotrophin), a well-known indicator of fibrotic aging, was by far the most significant frailty-associated protein in the BLSA (adjusted *P* value 5.069 × 10^−16^). COL6A3 has been associated with mortality and a range of noncommunicable chronic diseases linked to aging, including idiopathic pulmonary fibrosis, chronic kidney disease, type 2 diabetes and cardiac dysfunction, among others^59^. Remarkably, COL6A3 is also among the top five proteins associated with prefrailty and frailty in the UK Biobank^60^, independently validating the robustness of this finding in humans. Although exploratory, these findings collectively support the translational relevance of mouse serum proteomics and suggest that at least a subset of aging-associated circulating proteins is conserved in humans. While we expect the correlation of mice and human age-associated proteins as a whole to be limited due to the substantial influence of genetics and other factors on the circulating proteome, we speculate that a key subset of circulating proteins with mechanistic links to biology will be conserved and translationally relevant. Notable among these in our study are robust aging- and disease-associated proteins such as COL6A3 and lipopolysaccharide-binding protein (LBP), and sex-associated proteins such as Complement 8 (conserved association with the male sex) and adiponectin (ADIPOQ, conserved association with females).

Several limitations of this study warrant considerations. The cohort size was modest (*n* = 30 mice, 10 per age group), limiting statistical power and the generalizability of biological findings. In addition, the cross-sectional design precludes inference of within-individual aging trajectories that prevent the assessment of causal relationships over time. Nonetheless, the NP-MS workflow demonstrated strong reproducibility and sensitivity, enabling robust detection of age- and sex-associated signatures. Importantly, this workflow was successful within strict sample volume requirements down to 10 µl. Therefore, this method is compatible with the volume limitations of longitudinal mouse studies and can be easily adapted and scaled into large cohort studies with a longitudinal study design. Future phases of the SLAM, incorporating larger sample sizes and true longitudinal sampling will be essential to validate these associations, improve statistical power and generalizability, and more accurately capture temporal relationships and potential causal effects across the lifespan.

In summary, this study demonstrates that NP-based enrichment coupled with DIA-MS, complemented by a low-volume PEA cytokine assay, enables deep, quantitative serum proteomics from low volumes, compatible with longitudinal mouse studies. An NP-based MS approach markedly improved the proteome coverage, while PEA offered crucial sensitivity for detecting inflammatory cytokines; the minimal overlap between the two methods underscores their synergistic value in collectively enhancing the depth of circulating proteome analysis. The workflow captures biologically meaningful age-, sex- and stage-specific changes in the circulating proteome and reveals conserved signatures associated with human frailty. Looking ahead, advances such as top-down^61–63^ and artificial intelligence-driven computational modeling^64^ hold promise for extending NP-based approaches by resolving intact proteoforms, characterizing post-translational modifications and improving the mechanistic interpretation and predictive modeling of circulating biomarkers. Overall, while these findings are cross-sectional, they establish a robust methodological framework to enable longitudinal and mechanistic investigations of aging and biomarker discovery in sample-limited mouse studies and demonstrate translation relevance to human health.

## Methods

### Study design

All studies were compliant with ethical regulations. Mice experimental protocols were conducted according to the guidelines and approved by the Animal Care and Use Committee of the National Institute on Aging, NIH, under protocol number 458-TGB-2027. This study comprised two main phases: (1) optimization and benchmarking of sample preparation and LC–MS workflow in a feasibility study and (2) biomarker discovery in an aging mouse cohort using an optimized proteomics workflow coupled with a bioinformatic analysis (Fig. 1). For phase 1, the feasibility of detecting proteins in low volumes with the Proteograph workflow was assessed from a single pooled mouse sample. The quantitative benchmarking study was carried out similarly to previously described LFQ-Bench studies^28^, using defined mixtures of plasma from bovine and human purchased commercially (Innovative Research) at five different ratios (*n* = 5 each). For phase 2, we assessed serum samples from 30 inbred strain C57BL/6J mice (*Mus musculus*) selected from SLAM at three different ages: young (12 months), old (24 months) and geriatric (30 months), with 10 mice chosen at each age group (50% female in each group). Here, no statistical method was used to predetermine sample size. To improve statistical power, an additional 30 mice were included in the PEA analysis. SLAM is an ongoing longitudinal cohort-based investigation of normative aging conducted at the National Institute on Aging (NIA)^11^. The serum proteomics reported here sample a cross-sectional subset of SLAM, in which different mice were sampled at each age rather than repeatedly sampling the same individuals over time. Accordingly, the age-associated patterns reflect between-group differences, not within-individual longitudinal trajectories. Inbred B6 (stock no. 000664) mice of both sexes were born at JAX, shipped to the NIA animal facility at 12 weeks of age. Animal husbandry, environmental factors and diets are described elsewhere^65^. In brief, animal rooms were maintained at 22.2 ± 1 °C and 30–70% humidity. The lights were turned off at 18:00 and back on at 06:00 each day. Mice were group housed (four mice per cage) and fed an NIH-31 diet ad libitum with free access to water. Mice were housed in rooms maintained at 22.2 ± 1 °C and 30–70% humidity. Mice were monitored twice daily for health status, and veterinary care was administered as necessary. Routine tests are performed to ensure that mice are pathogen-free and sentinel cages are maintained and tested according to the American Association for Accreditation of Laboratory Animal Care (AAALAC) criteria.

### Serum preparation

Serum preparation from blood was performed in serum separator tubes (Sarstedt AG, 41.1378.005). The serum was aliquoted and kept frozen at −80 °C until proteomic analysis. Pooled mice serum was used for a feasibility study of workflow optimization.

### Sample preparation for proteomic analysis

All serum samples were processed using the Seer Proteograph workflow on an automated SP100 instrument^15,29^, using a randomized and age- and sex-balanced 96-well plate design to minimize batch effects and procedural bias. The workflow uses a subset of engineered NPs with diverse surface chemistries, including variations in charge and functional groups, built on superparamagnetic iron oxide NP (SPION) cores to enable magnetic capture, as previously described^15^. Although the exact characteristics of the commercially available NP kits are proprietary, they are selected from among the library of NPs characterized previously characterized in more detail^15^. Each NP forms distinct and reproducible protein coronas that, when used alone or in combination, enhance proteome depth. In brief, serum samples were diluted with TE buffer (10 mM Tris, 1 mM disodium EDTA and 150 mM KCl) and 0.05% CHAPS and mixed with NP suspension, here each sample was incubated at 37 °C for 1 h with shaking at 300 rpm with five proprietary, physicochemically distinct NPs for protein corona formation. Incubation allows high-affinity proteins to displace high-abundance proteins, resulting in a reproducible protein corona on each NP surface that probes the depth of the serum proteome. After incubation and magnetic separation, bound proteins were reduced, alkylated, and digested with Trypsin/Lys-C using a digestion kit^15^. Peptides were purified by cleanup cartridge, and their concentration was measured by a quantitative colorimetric peptide assay kit from Thermo Fisher Scientific, and finally lyophilized by vacuum dried and stored at −80 °C until analysis by MS. All samples were processed under identical workflow conditions using an automated workflow, ensuring uniform handling and minimizing technical variability during protein corona formation, digestion and peptide clean up

### DIA LC–MS/MS analysis

Peptide samples were reconstituted with 10 μl loading buffer composed of 2% acetonitrile (ACN) and 0.1% formic acid (FA) in LC–MS grade water. Indexed retention time standards (iRTs, Biognosys) were spiked according to manufacturer’s instructions before MS analysis. All samples were analyzed using a Q Exactive HF-mass spectrometer (Thermo Fisher Scientific) interfaced with an Ultimate 3000 RSLC nanoLC (Thermo Fisher Scientific). Reverse-phase LC was conducted with two mobile phases (A and B). Mobile phase A consisted of 0.1% FA in water, and mobile phase B contained 0.1% FA in ACN. Peptides were loaded onto nanoViper Acclaim PepMap 100 trap column (3 μm C_18_ particle, 75 μm × 2 cm Thermo Fisher Scientific). Then separated on a PepMap RSLC column (2 μm C_18_ particle, 75 μm × 500 mm, Thermo Fisher Scientific). The trap and separation column were maintained at 45 °C within the column oven and EASY-spray ionization source, respectively. The nanosource capillary temperature was set to 320 °C and the spray voltage was set to 2.0– 2.4 kV. For the feasibility and aging studies, peptides were separated using a 120 min linear gradient from 5% to 30% of solvent B at a constant flow rate of 250 nl min^−1^, with a total run time of 180 min. For quantitative benchmarking samples, a shorter 65 min gradient (2–30% of solvent B) was applied using same column and flow rate, with a total run time of 100 min.

All MS data were acquired in DIA mode. MS2 scans were configured to acquire 50 × 24 *m*/*z* DIA spectra (24 *m*/*z* isolation window) spanning the mass range 400–1,000 *m*/*z*, staggering the DIA window placement by about half an isolation window (12.7 *m*/*z*) during alternating cycles as previously described^66^. The MS1 scan spanning 400–1,000 *m*/*z* was acquired at 60,000 resolution with an AGC target of 3–5e^6^ and a maximum injection time of 60–100 ms. MS2 DIA scans were acquired at 30,000 resolution with an AGC target of 1e^6^ and maximum injection time 50–70 ms spanning the same range using 24 *m*/*z* windows with normalized collision energy of 27–30% used for precursor fragmentation in HCD mode. The acquisition of samples was block-randomized to avoid bias^67^.

### MS data analysis

All raw MS files from the feasibility and aging studies were searched using the DIA-NN v1.8.1 algorithm within Proteograph Analysis Suite v2.0. Peptide identification and quantification were performed with library-free mode searching MS/MS spectra against an in silico-generated spectral library of UniProtKB mouse FASTA database (UP000000589_10090). For quantitative benchmarking study, MS data were analyzed using the same pipeline with a combined database containing both human and bovine proteomes. The search parameters were set as follows: trypsin protease, 1 missed cleavage, fixed modification of Cys Carbamidomethylation, no Met oxidation, N-terminal Met excision, peptide length of 7–30 amino acids, precursor range of 300–1,800 *m*/*z* and fragment ion range of 200–1,800 *m*/*z*. The FDR for peptides and proteins was set at 1% (*Q* value). MS1 and MS2 mass accuracy was set to 10 ppm with Heuristic protein inference enabled. Peptide quantification was performed using a max representation (MaxRep) approach, where the single quantification value for a particular peptide represents the quantitation value of the NP that is most frequently measured across all samples with the highest intensity, and standard peptide-to-protein rollup was used on selected peptide values. During workflow optimization, for comparison, MaxLFQ^30^ was also utilized for NPs and peptide to protein rollup using a MaxLFQ R-script.

### PEA

Cytokines in the mouse serum samples were quantified using the Olink Target 48 Mouse Cytokine panel based on PEA technology (Olink Proteomics, Thermo Scientific) on the Olink Target 48 Mouse Cytokine kit (934002). The assay kit-provided oligonucleotide-labeled antibodies were designed to selectively bind with their respective protein targets. Assays were performed by Assay Gate according to manufacturer’s instructions. PEA and MS datasets were processed and normalized independently using platform-specific pipelines due to differences in assay chemistry and dynamic range. Quality control assessments of the PEA assay include inclusion of process controls (for key protocol steps: incubation, extension and detection), a triplicate sample control to monitor intra-run precision, duplicate negative controls and triplicate calibrator samples for normalization between batches^68^. The limits of detection and quantification, and precision of each protein measurement have been previously reported^68^. All samples used for this study passed internal and external quality control metrics.

### Statistics and reproducibility

In this study, serum samples were collected from a cohort that represents both sexes and multiple stages across the murine lifespan. MS acquisition of samples was performed in random order by an instrument operator that was blind to the sample metadata, with the inclusion of intermittent quality control samples for monitoring system suitability. A sufficient number of samples were included to detect statistically significant differences in key aging traits, as previously described in detail^69^. This allowed for identification of circulating protein and pathway associations with aging. Raw precursor intensities were normalized to global median intensities. For Spearman and Pearson modeling, intensities were centered and scaled. No data were excluded from the analyses. Data distribution was assumed to be normal but this was not formally tested. The proteomic experiments have not been replicated by our group, although some of the results pertaining to sex-associated proteins have been independently verified in published literature (Supplementary Table 4).

Bioinformatic analysis was conducted in RStudio version 4.2.2. Expression levels of genes identified on the peptide and protein levels were compared between NP and rollup method using spearman correlation (stats package version 4.2.2). Pearson modeling was used to determine proteins and peptides associated with age within the cohort (stats package version 4.2.2). *P* value adjustment was performed for differential protein expression analysis using the Benjamini–Hochberg method.

### Clinical data

All human studies were compliant with ethical regulations. We used clinical data of 1,275 participants of median age 70 years, of which 53% are female. from the BLSA cohort to determine the association of proteins with frailty index and aging using a previously published dataset^7^. The BLSA study is a population-based study that started in 1958 to evaluate the contributors of healthy aging in subjects recruited from the DC/Baltimore metropolitan area that are 20 years old and older^32,70,71^. The BLSA protocol (03AG0325) was approved by the institutional review board of the National Institute of Environmental Health Science, part of the National Institutes of Health. All participants provided written informed consent.

### SOMAscan assay

The BLSA plasma proteomic study involved profiling for 7,596 SOMAmers using the 7 K SOMAscan assay at the Trans-NIH Center for Human Immunology and Autoimmunity, and Inflammation (CHI), National Institute of Allergy and Infectious Disease, National Institutes of Health (Bethesda, MD). Proteomic assessment and data normalization for each of the assays were conducted as reported previously^72,73^. Plasma protein concentrations were directly proportional to the abundance of the SOMAmer reagents that were reported in relative fluorescence units. The reliability and variability of the SomaScan assay measurements has been previously rigorously evaluated and applied in the BLSA^73,74^.

### Frailty index

A composite frailty score was created as described previously in the BLSA^75^. Frailty index procedures followed those established by Searle et al.^76^ and included 44 common measures of frailty, with the addition of diabetes. Aspects of the composite frailty score include reported difficulty carrying out 15 basic life tasks, including walking up 10 steps, and lifting 10 pounds; self-rated health from a health status survey; five items indicating emotional state from the Center for Epidemiologic Studies Depression Scale, including feeling lonely or depressed; four items indicating cognitive status from the mini-mental state examination, including orientation to time and memory; 14 binary health conditions including cancer and cognitive impairment, among others; recent weight loss, low physical activity, walking slowness and grip weakness.

### Pathway enrichment and visualization

Overrepresentation and gene set enrichment analysis were conducted using the Cluster Profiler Package version 4.6.0 (ref. ^77^).

### Reporting summary

Further information on research design is available in the Nature Portfolio Reporting Summary linked to this article.

## Supplementary information

Supplementary Information Supplementary Table 1.

Reporting Summary

Peer Review File

Supplementary Table 2 Olink quantitative proteomic data.

Supplementary Table 3 Mass spectrometry quantitative proteomic data and statistics.

Supplementary Table 4 Validation of top sex associated proteins with other study.

## Source data

Source Data Fig. 2 Raw numerical data from each graph representing mass spectrometry-based proteomic measurements.

Source Data Fig. 3 Raw numerical data from each graph representing Olink-based proteomic measurements.

Source Data Fig. 4 Data underlying Fig. 4 visualizations depicting age-related proteomic changes.

Source Data Fig. 5 Data underlying Fig. 5 visualizations depicting sex-associated proteomic changes.

Source Data Fig. 6 Data underlying Fig. 6 visualizations comparing age-related proteomic changes in mouse versus human.

Source Data Extended Data Fig. 1 Data underlying Extended Data Fig. 1 visualization of mass spectrometry-based proteomics measurements from a feasibility study.

Source Data Extended Data Fig. 2 Data underlying Extended Data Fig. 2 visualizations summarizing nanoparticle rollup and quantification approaches.

Source Data Extended Data Fig. 3 Data underlying Extended Data Fig. 3 correlations of sex-associated proteins in the present study versus two published studies.

## Data Availability

All raw MS data files and associated quantitative data, metadata, and sequence databases used in this study are available on the MassIVE repository (dataset identifier: MSV000098399). FTP download link: ftp://massive-ftp.ucsd.edu/v10/MSV000098399/.
