# Supplementary material for: Deep and quantitative proteomic profiling of low-volume mouse serum in a cross-sectional analysis of aging
Source: Nat Aging. 2026 Nov 2;6(11):2504–16. doi: 10.1038/s43587-026-01242-8 (PMC13754568; doi:10.1038/s43587-026-01242-8)
Supplement: Supplementary file 1 [file 43587_2026_1242_MOESM1_ESM.pdf]

nature aging

Supplementary information

https://doi.org/10.1038/s43587-026-01242-8

Deep and quantitative proteomic profiling of low-volume mouse serum in a cross-sectional analysis of aging

In the format provided by the authors and unedited

Supplementary information

Supplemental Table 1. SLAM Investigator titles and names (in alphabetical order)

Titles	Names
**Investigators**	Nathan Basisty, Ph.D., Rafael de Cabo, Ph.D., Luigi Ferrucci, M.D., Ph.D., Lenore J Launer, Ph.D., Stephanie A. Studenski, M.D., M.P.H.
**Staff Scientists**	Michel Bernier, Ph.D., Simonetta Camandola, Ph.D., Julian Candia, Ph.D., Nathan L. Price, Ph.D., Eric J. Shiroma, Ph.D.,
**Biologists**	Daniel Abaneme, Nirad Banskota, Katina Krasnec, Ph.D., Andrew O'Rourke, Ericka Oglesby, Adeniyi Owoeye, Louis Rezanka, Ph.D., Chava Roberts
**Postdoctoral Fellows**	Irene Alfaras, Ph.D., Burcu Can, DVM, Clara di Germanio, Ph.D., Burcin Duan Sahbaz, Ph.D., Eleonora Duregon, M.D., Ph.D., Maria Emilia Fernandez, Ph.D., Ignacio Navas Enamorado, Ph.D., Jorge Martinez-Romero, Ph.D., Sarah J. Mitchell, Ph.D., Hazal Ozturk, DVM Gurgen, Ph.D., Dushani L. Palliyaguru, Ph.D., Camila Vieira Ligo Teixeira, Ph.D.
**Graduate Students**	Joshua Preston
**Post Baccalaureate Fellows**	Sara Abdo, Gelareh Abulwerdi, Ahmed Ali, Daniela Barata Herrera, Naa-Oye Bosompra, Kelsey Bullock, Meaghan Cabassa, Melissa Carpenter, Dolly Chowdhury, Ethan Durham, Margaux Ehrlich, Ayanna Kakar, Kiaya Keckler, Farzin Eshaghi, Frances Fran, Tim Frawley, Vishant Gandhi, Joseph Garcia, Vincent Gutierrez, Samuel Hamilton, Stephan Emilio Haraldsson, Jonathan Hirschenson, Jennifer Hon, Phu T. Huynh, Tamzin Kaiser, Krystle Kalafut, Jonathan Kato, Mounisha Kovour, Priya Krishna, Dimitri Lin, Brandon Lamotte, Jacqueline Moats, William Mueller, Hector Mora, John K. Nam, Kedhar Nayaran, Sandy Ng, Michael A. Petr, Ava Pishgar, Shiva Puttagunta, Annamaria Rudderow, Tyler Rynesmith, Stephen Scheeler, Hikari Tanaka, Diego Tanton, Rhea Tiwari, Romania Voloshchuk, Erin Wade, Devin Wahl, Sarah Wong, Tyemur Zavar
**Summer Students**	Nelson Castillo-Rivera, Jack Donahue, Hector Haddock, Annelise Lakatta, Dongwon Lee, Sam Lee, Jean Morales Santiago, Diego Tanton.
**Animal Technicians**	Ranora Cook, Sharon Ensor, Ashley Jordon, Lauryn Meyer
