# Supplementary material for: Deep and quantitative proteomic profiling of low-volume mouse serum in a cross-sectional analysis of aging
Source: Nat Aging. 2026 Nov 2;6(11):2504–16. doi: 10.1038/s43587-026-01242-8 (PMC13754568; doi:10.1038/s43587-026-01242-8)
Supplement: Supplementary file 3 [file 43587_2026_1242_MOESM3_ESM.pdf]

nature portfolio

Peer Review File

Deep and Quantitative Proteomic Profiling of Low Volume Mouse Serum in A Cross-Sectional Analysis of Aging

Corresponding Author: Dr Nathan Basisty

A version of this paper was originally rejected for publication by Nature Aging, however that decision was reconsidered after appeal by the authors.

Version 0:

Reviewer comments:

Reviewer #1

(Remarks to the Author)
The authors developed a highly efficient proteomics workflow using the nanoparticle protein corona approach to achieve deep proteome coverage of low-volume mouse serum samples and to study the serum proteome changes as a function of age and sex. They also investigated the connection between proteome changes and glucose level/body composition. They identified nearly 4000 proteins from as low as 20 microliters of mouse serum sample using only a Q-Exactive HF mass spectrometer, representing a substantial improvement in sensitivity and proteome coverage for mouse serum samples. The quantitative analysis of the mouse serum samples across three ages (12, 24, and 30 months) offered new insights into the molecular mechanisms of aging. I have several comments to help the authors further strengthen this work.

1. My main suggestion is related to comparing the data generated in this study with some large-scale human plasma proteomics data published recently using the UK Biobank. The comparison will be necessary to build the confidence that the mouse study will shed valuable light on the human aging, which is critical for this study.

2. The NP protein corona composition can be strongly influenced by other molecules in the plasma sample, like metabolites. [PMID: 39511193] Therefore the heterogeneity across plasma/serum samples may influence the protein corona substantially, nothing related to the aging. Therefore, the differentially expressed proteins across the three ages studied here need some additional verification using some high-throughput antibody-based approach. Choosing several proteins from the differentially expressed pool for additional confirmation will be sufficient.

3. Some new frontiers of NP protein corona study should be cited. for example, top-down proteomics (PMID: 40764671 ; PMID: 39276099; PMID: 39312774) and AI (PMID: 40190726).

4. Based on my understanding, this study is not a longitudinal study because they studied different mice at three ages. The authors should clarify this point in the manuscript.

5. A proteomics study of low-volume mouse serum samples using nanoparticle protein corona approach was published recently. The authors should cite and mention this paper. [PMID: 40726225]

Reviewer #2

(Remarks to the Author)
I co-reviewed this manuscript with one of the reviewers who provided the listed reports. This is part of the Nature Aging initiative to facilitate training in peer review and to provide appropriate recognition for Early Career Researchers who co-review manuscripts.

Reviewer #3

(Remarks to the Author)
This study introduces and validates a nanoparticle-based, data-independent acquisition (DIA) mass-spectrometry workflow

that enables deep and quantitative proteomic profiling of low-volume mouse serum across the lifespan, complemented by a proximity extension assay for cytokines (Olink). The authors identified around 4,000 proteins including >200 senescence-associated markers, and, using 1 µL of additional serum, quantified 39 further cytokines typically missed by MS. The quantitative accuracy was high (median CV < 10%) and strong agreement between NP fractions was reported. The authors applied the pipeline to 30 mice aged 12, 24 and 30 months and revealed age- and sex-dependent proteomic signatures and correlations with glucose and body fat. The paper demonstrates a good level of benchmarking, and the complementary use of Olink to capture low-abundance cytokines was appropriate. The manuscript is well-written, and the methodology is sound. Although the paper reports interesting outcomes, it has quite important weaknesses; one of the major issues is the small, cross-sectional cohort (limiting statistical power and causal inference), the absence of independent validation or direct human translation. One interesting aspect of the paper though, is the spike in experiment, wherethe author shows that nanoparticle corona preserves the quantitative differences and fold changes and is truly representing the serum quantities of proteins.
Finally, the critical roles of metabolite and lipid variations and their effects on protein corona have not been considered (several reports revealed that variations in metabolites and lipid profiles of plasma proteomics can substantially change the protein corona profile at the surface of nanoparticles); therefore, it is not clear that the observed variations originate from plasma proteome variations or reflection of metabolomics and lipidomic profile variation in plasma that caused changes in the composition of protein corona. This could be very important, as the authors were interested in proteomics signatures associated with glucose and body fat.

I think in page 6, Supplementary Figure 2A is wrongly cited, as it does not show the CV.
Overall, the figure quality is not satisfactory, for example, too many labels, font size consistency and axis lines should be corrected/added.

Version 2:

Reviewer comments:

Reviewer #1

(Remarks to the Author)
The authors did a nice job in the revision and addressed my concerns properly.

Reviewer #3

(Remarks to the Author)
I believe the authors have implemented satisfactory changes in the manuscript, in response to my feedback and comments.

Open Access This Peer Review File is licensed under a Creative Commons Attribution 4.0 International License, which permits use, sharing, adaptation, distribution and reproduction in any medium or format, as long as you give appropriate credit to the original author(s) and the source, provide a link to the Creative Commons license, and indicate if changes were made.
In cases where reviewers are anonymous, credit should be given to 'Anonymous Referee' and the source.

The images or other third party material in this Peer Review File are included in the article's Creative Commons license, unless indicated otherwise in a credit line to the material. If material is not included in the article's Creative Commons license and your intended use is not permitted by statutory regulation or exceeds the permitted use, you will need to obtain permission directly from the copyright holder.
To view a copy of this license, visit https://creativecommons.org/licenses/by/4.0/

REPONSE TO REVIEWER COMMENTS

NATAGING-A10220: Deep and Quantitative Proteomic Profiling of Low Volume Mouse Serum: A Cross Sectional of Aging

We are grateful to the editor and all the reviewers for the thorough review of the manuscript and opportunity to appeal the publication decision. We have thoroughly responded to and incorporated the suggested changes to the best of our ability in the revised manuscript. The revision includes the addition of the requested new analysis exploring the translational potential of our results in humans by comparing with a large-scale proteomic dataset, and associated clinical parameters, from the Baltimore Longitudinal Study of Aging (BLSA). We believe the manuscript has substantially improved in quality, focus, and rigor as a result of the feedback from reviewers.

Below are point-by-point responses to reviewer comments:

Reviewers' Comments:

REVIEWER #1:

1. My main suggestion is related to comparing the data generated in this study with some large-scale human plasma proteomics data published recently using the UK Biobank. The comparison will be necessary to build the confidence that the mouse study will shed valuable light on human aging, which is critical for this study.

Response: We appreciate the suggestion and agree that comparison with large-scale human plasma proteomics datasets is important to establish confidence that mouse serum proteomics can inform human aging biology. To address this point, the revised manuscript incorporates a cross- species comparison between the mouse proteomic signatures identified in this study and human plasma proteomics from Baltimore Longitudinal Study of Aging (BLSA) and published UK Biobank proteomics that support the translational relevance of the mouse data. While our access to the UK Biobank data is forthcoming, given our initial findings, we are quite enthusiastic about further expanding the analysis into the expansive proteomic dataset.

The newly generated analysis has been included in the results as a main figure (**Fig. 6**). Our analysis reveals that 50% of the age-associated proteins identified in this mouse study are also significantly age-associated in the BLSA. Interestingly, these results suggest a common

circulating aging signature of extracellular matrix, endoplasmic reticulum, and lysosomal protein components in humans and mice. We assessed the association of these proteins with frailty, a key clinical aging endpoint, in BLSA and found that five significantly associated with frailty: COL6A3, LBP, PIGR, IGFBP1 and HPX. By far, the ECM protein COL6A3 is the most significant among these (adj. p-value = 5.07e-16). These findings are consistent with recent evidence of age-associated increase in ECM components in Nature Aging (PMID: 41023316), and a broad range of literature associating COL6A3 (aka Endotrophin, the uncleaved precursor) with mortality and chronic diseases (PMID 38871093). Notably, COL6A3 is among the top 5 proteins associated with pre-frailty, and frailty, in the UK Biobank (PMID 39535692), independently validating the association with human frailty observed in BLSA. Overall, these results support clinical relevance of the findings of our study and demonstrate the mouse study can shed light on human aging. We have included the new analysis into the results, figures, and discussion, accordingly, as shown below.

New Figure, Fig. 6:

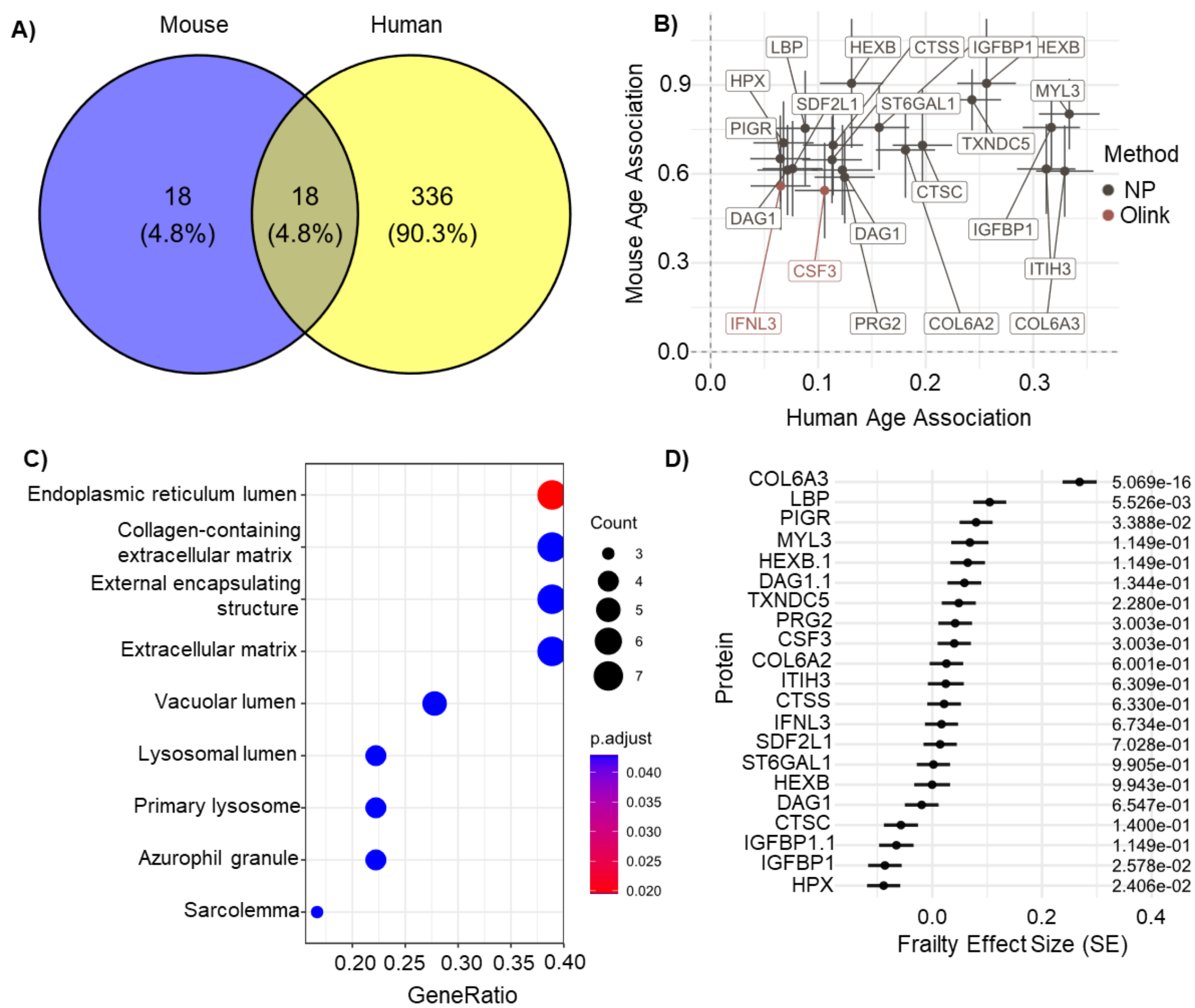

“**Figure 6: Circulating proteins associated with age and frailty in mice and humans** A) Venn diagram illustrating proteins identified as positively age-associated using Pearson linear modeling across NP-MS workflow and the O-link platforms in the cohort analyzed in this study (left blue), and proteomic measurements (SomaScan 7k assay) in the BLSA (right yellow), modeled as: age ~ protein + sex (Total proteins quantified across both datasets, N = 1742). B) Proteins significantly associated with age in mice and humans, determined using Pearson linear modeling (age ~ protein + sex). Color coding indicates detection method in this study. C) Overrepresentation analysis of proteins significantly upregulated with age in both species, annotated by cellular component. D) Associations between circulating proteins and frailty assessed using Pearson linear modeling across the cohort analyzed in this study and the BLSA, based on a 44-component frailty index and the model: frailty index ~ protein + age + sex. “

New analysis described in the Results section, page-9, line-253:

“To assess cross-species concordance, we compare age-associated circulating proteins in mice with human plasma proteomics from the BLSA (35, 36). Among 1742 proteins quantified in both datasets, Pearson Linear modeling (age ~ protein + sex) identified 18 proteins, representing 5% of the total proteins or 50% of mouse age-associated proteins, to be significantly (**Fig. 6A**) and positively (**Fig. 6B**) associate with age in both mice and humans. Overrepresentation analysis of shared upregulated proteins with age in both mice and humans revealed enrichment for cellular component categories (**Fig. 6C**) including endoplasmic reticulum, extracellular matrix, and lysosomal proteins, among others, and suggesting shared categories of age- associated circulating factors at a pathway-level. To assess the relevance of these findings with age-associated clinical phenotypes, we evaluated the associations of these proteins with frailty index, a 44-component composite score, in the BLSA. Notably, five proteins including COL6A3, LBP, PIGR, and IGFBP1, and HPX were significantly associated with frailty index in the BLSA (Frailty Index ~ Protein + age + sex; **Fig. 6D**). Collectively, these results link conserved proteomic aging signatures to functional health decline in humans and suggest that aging-associated biomarkers derived from mice may be relevant in humans.”

We have emphasized these cross-species findings in the Abstract, page-2:

“Cross-species comparison with human plasma proteomics from the Baltimore Longitudinal Study of Aging (BLSA) identified conserved age-associated circulating proteins, several of which were linked to frailty in humans.”

Finally, we elaborated on the significance of these results in the Discussion, page-13, line- 394:

"To place the mouse findings in a translational context, we compared age-associated circulating proteins identified here with human plasma proteomics from BLSA. Despite differences in species, cohort design, and analytical platforms, a subset of proteins, representing half of the mouse age-associated proteins, were concordantly age- associated in humans (**Fig. 6A-B**), indicating conserved features of the circulating aging proteome. Interestingly, collagen-containing extracellular matrix proteins were overrepresented among these, consistent with recent reports of the association of extracellular matrix components, including collagen, with aging in mouse circulation (66). Moreover, several of these shared proteins, including COL6A3, LBP, PIGR, IGFBP1, and HPX, were also significantly associated with frailty in humans, linking shared circulating proteomic signatures to functional health decline. Notable among these, Collagen Type VI Alpha 3 Chain (COL6A3, a.k.a. Endotrophin), a well-known indicator of fibrotic aging, was by far the most significant frailty-associated protein in the BLSA (adjusted p-value = 5.069e-16). COL6A3 has been associated with mortality and a range of non-communicable chronic diseases linked to aging, including idiopathic pulmonary fibrosis, chronic kidney disease, type 2 diabetes, cardiac dysfunction, among others (67). Remarkably, COL6A3 is also among the top five proteins associated with pre-frailty and frailty in the UK Biobank (68), independently validating the robustness of this finding in humans. Although exploratory, these findings collectively support the translational relevance of mouse serum proteomics and suggest that at least a subset of aging-associated circulating proteins is conserved in humans. While we expect the correlation of mouse and human age-associated proteins as a whole to be limited due to the substantial influence of genetics and other factors on the circulating proteome, we speculate that a key subset of circulating proteins with mechanistic links to biology will be conserved and translationally relevant. Notable among these in our study are robust aging- and disease-associated proteins such as COL6A3 and lipopolysaccharide-binding protein (LBP), and sex-associated proteins such as Complement 8 (conserved association with the male sex) and adiponectin (ADIPOQ, conserved association with females)."

2. The NP protein corona composition can be strongly influenced by other molecules in the plasma sample, like metabolites. [PMID: 39511193] Therefore the heterogeneity across plasma/serum samples may influence the protein corona substantially, nothing related to the aging. Therefore, the differentially expressed proteins across the three ages studied here need some additional verification using some high-throughput antibody-based approach. Choosing

several proteins from the differentially expressed pool for additional confirmation will be sufficient.

Response: This is an important point and we appreciate the importance of considering potential confounding effects on the composition of the protein corona, and the critical need to demonstrate the quantitative aging changes derived from the protein corona are representative of differences in the original serum samples. We have thoroughly addressed and acknowledged these concerns with the inclusion of additional analysis and discussion. We hope the combination of responses presented below sufficiently address the underlying concerns of the reviewer.

First, we contend that the 'gold standard' method to demonstrate whether changes in the protein corona are representative of 'real' changes in the original serum samples at the proteome level (across a wide range of proteins) is to perform quantitative benchmarking (PMID: 27701404) on the same nanoparticles utilized in our study. This concern motivated us to conduct our own independent benchmarking study, shown in **Fig. 2A-B** (see below). Given that human and bovine blood are distinct in their compositions (including metabolites and lipids), if lipids/metabolites had a significant confounding effect on the composition of the protein corona, our expectation would be that our observed protein ratios would be skewed and inaccurate. However, our data suggests that on average the quantitative ratios of proteins in the protein corona are representative of true ratios of the original blood samples across a range of dilutions (5% - 100%).

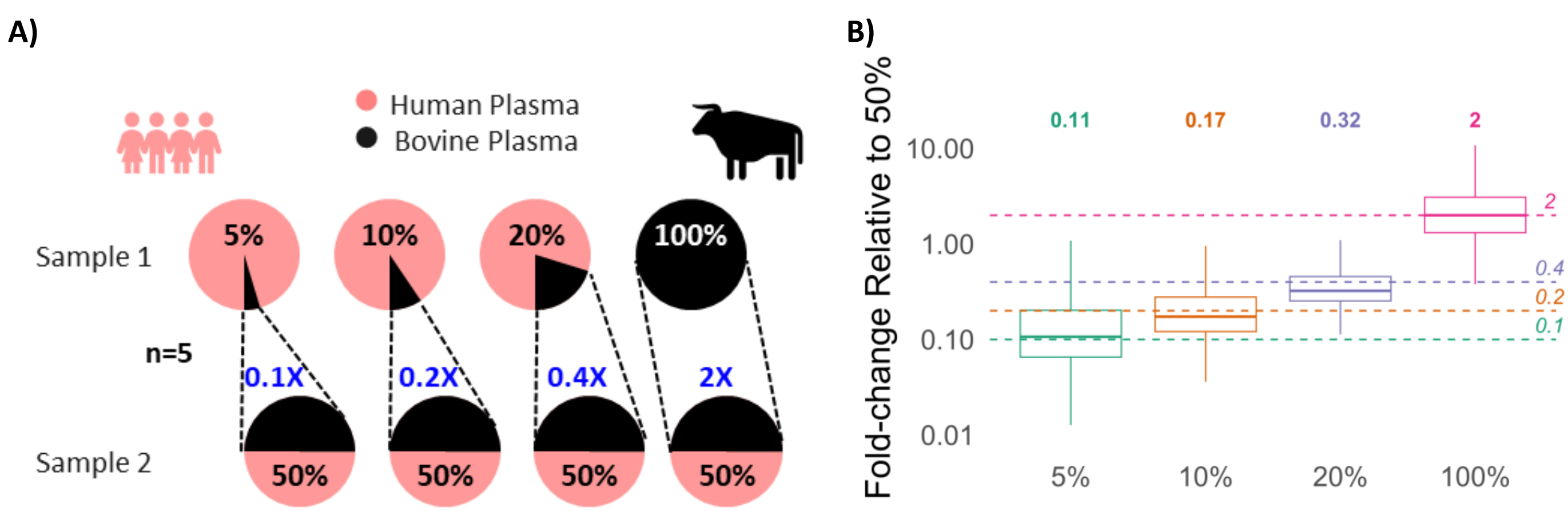

"Figure 2: Qualitative and quantitative evaluation of proteomic data generated NP-coupled MS workflow. A) A quantitative benchmarking study was performed using five fixed ratios of

human and bovine plasma (n=5 each). B) Boxplot showing the measured peptide ratios in each dilution versus the 50% bovine plasma sample. Dotted lines depict the expected ratios.**"**

Second, we emphasize that published literature, which has more rigorously and comprehensively examined characteristics of the protein corona and binding properties of the nanoparticles utilized in this study, suggests that the effect of lipids and matrix do not significantly impact quantitation. We note that the nanoparticles used in this study are distinct from the plain polystyrene nanoparticles in the study cited by the reviewer (PMID: 39511193) and may not exhibit the same binding behavior. The surface-functionalized superparamagnetic iron nanoparticles in this study are characterized in more detail elsewhere (PMID: 35275789). Lipid spike-in experiments have been done on the same nanoparticles we used, showing that spiking varying amounts of lipid do not significantly affect the number/intensity of proteins (PMID: 32699280, Nature Comms - last paragraph of p5 & first on p6, Fig. S4 and S5). Another paper in JPR (PMID: 41105214) reported on the minimal impact of background matrix (presumably with different lipid/metabolite compositions) on quantitative accuracy. We have noted these additional studies in the discussion (quoted below).

We explained now in the revised “Discussion” section for more clarity, page-10, line- 295:

“Concerns have been raised that NP- coronas may be sensitive to non-protein serum components, such as lips or metabolites, potentially introducing variability unrelated to biological differences. While recent work has shown that small molecules can influence protein corona composition derived from plain polystyrene nanoparticles (40), multiple independent studies of the surface-functionalized superparamagnetic iron nanoparticles employed in this study suggest that these effects are not significant. Lipid-spike-in experiments using the same nanoparticles showed no appreciable changes in corona protein identities or intensities (15). Similarly, a prior report suggests minimal impact of background matrix on quantitative accuracy (41). Our own quantitative benchmarking data further supports this conclusion, showing high reproducibility and accurate quantification of known fold-changes in samples generated from mixing human and bovine blood, each containing a distinct composition of lipids, metabolites, and other components, in a range of fixed dilutions spanning 5% to 100% (**Fig. 2B**). Together, these findings indicate that matrix-derived components did not significantly alter corona composition in a manner that distorts relative protein quantification in this study and mitigate concerns matrix effects introduced variability unrelated to aging.”

Third, we attempted to address the reviewer's request for independent validation of the aging changes measured in protein coronas with additional analysis and comparison with literature. Targeted cytokine measurements with the Proximity Extension Assay (PEA), an antibody-affinity-based platform, were a major component of this study. To perform a cross-platform comparison of a protein measured by both methods, nanoparticle-based MS versus antibody-based, we searched for overlapping protein observations between datasets and found a single protein – IL16 – with enough observations across mice to perform a comparison (**Fig. 3C-D**, 11 mice with matched measurements in both platforms). Using linear modeling, we found significant correlation between platforms in the measurement of IL16 ($p=0.0026$, $R = 0.81$). This example further supports our claim of accurate representation of protein levels in the protein corona when compared with the original serum samples. To further validate whether our observed changes align with expected biology and proteomic measurements from prior studies, we performed an analysis comparing our measured changes to two published MS-based proteomic datasets in mouse blood that did not use nanoparticles. Although there are no prior mouse aging studies in blood to draw from, the two published studies examined sex-associated changes in mouse serum (PMIDs 37910662 and 34050187). Notably, apart from two proteins (Transcobalamin-2 and Hepatocyte growth factor activator), all sex-associated proteins from our study that were also reported in either published study were directionally aligned and correlated. This has been added as a supporting figure, **Fig. S3**, and is numerically detailed in **Table S4**. Overall, these findings further support that protein corona measurements in our study align with direct measurements in blood samples and accurately reproduce expected biological changes, such as sex-dimorphism.

New figure, **Fig. S3**:

A) B)

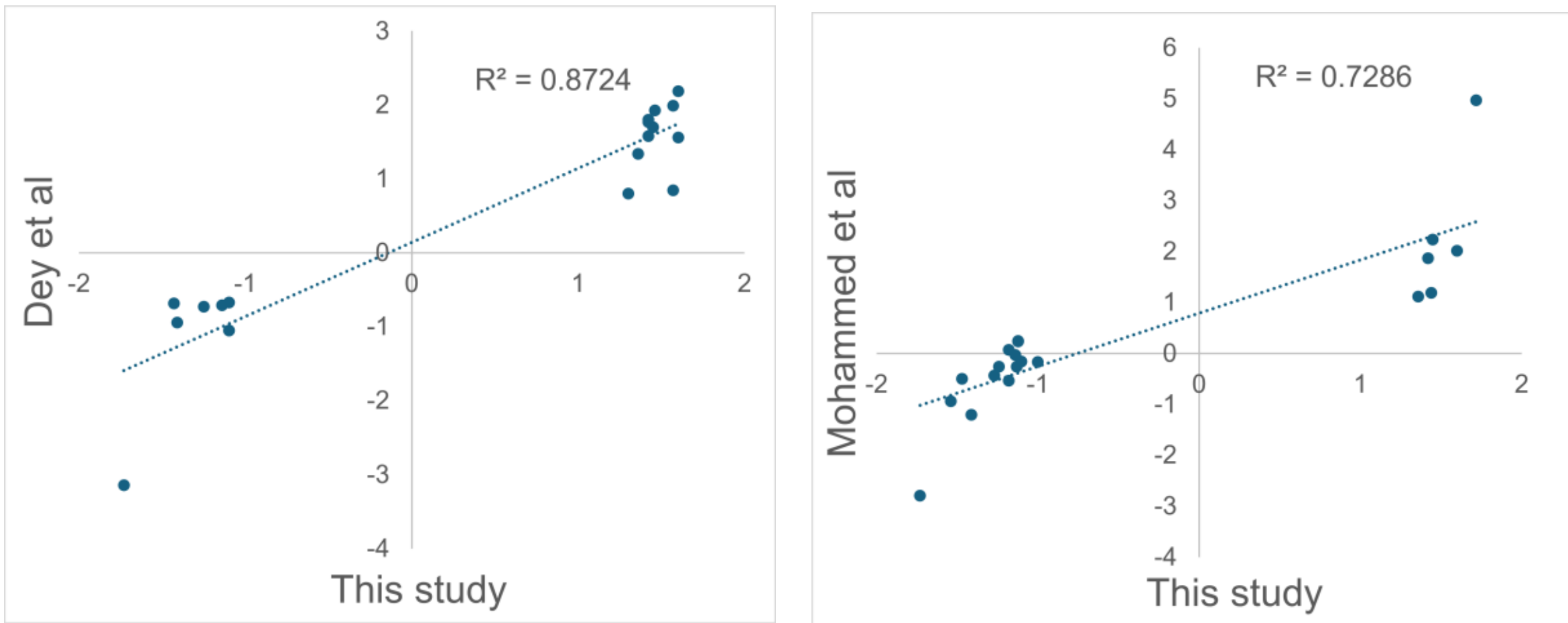

"Supplementary Fig. 3- Correlation of sex-associated circulating proteins between this study and published datasets. Correlation plots depicting sex-associated changes in our study versus those reported in A) Dey et. al. (PMID: 37910662); and B) Mohammed et. al. (PMID-34050187). Proteins in both studies show strong positive correlations with this study (R^2=0.8724 and R^2=0.7286, respectively), and consistent directionality of sex associated effects."

Supplemental Table 4: Validation of top sex associated proteins with other study

				**SLAM**	**Dey et. al. JPR,2023**	**Mohammed et. al. NPJ Syst. Biol. Appl. 2021**
**Sl. No.**	**Gene**	**Uniprot ID**	**Description**	**B-sex**	**log2(M/F)**	**log2(M/F)**
1	Serpina1e*	Q00898	Alpha-1-antitrypsin	**1.72**		**4.97**
2	C8g*	Q8VCG4	Complement component C8 gamma chain	**1.6**	**1.56**	**2.02**
3	C6	E9Q6D8	Complement component 6	**1.6**	**2.19**	
4	C7	D3YXF5	Complement component 7	**1.57**	**1.99**	

5	C8b*	Q8BH35	Complement component C8 beta chain	**1.45**	**1.7**	**2.24**
6	C8a*	Q8K182	Complement component C8 alpha chain	**1.42**	**1.77**	**1.87**
7	Ces3a	Q63880	Carboxylesterase 3A	**1.57**	**0.85**	
8	Mug1*	P28665	Murinoglobulin-1	**1.44**		**1.19**
9	Mup20	Q5FW60	Major urinary protein 20	**1.46**	**1.93**	
10	Crisp1	Q03401	Cysteine-rich secretory protein 1	**1.42**	**1.8**	
11	Mup3	P04939	Major urinary protein 3	**1.42**	**1.58**	
12	Egfr*	Q01279	Epidermal growth factor receptor	**1.36**	**1.34**	**1.12**
13	Serpina3k	P07759	Serine protease inhibitor A3K	**1.3**	**0.8**	
14	Tcn2	O88968	Transcobalamin-2	**-1.12**		**0.25**
15	Glycam1*	Q02596	Glycosylation-dependent cell adhesion molecule 1	**-1.73**	**-3.14**	**-2.79**
16	Adipoq*	Q60994	Adiponectin	**-1.54**		**-0.94**
17	Bche	Q03311	Cholinesterase	**-1.47**		**-0.5**
18	Serpina6*	Q06770	Corticosteroid-binding globulin	**-1.41**	**-0.94**	**-1.2**
19	Scg3	P47867	Secretogranin-3	**-1.43**	**-0.68**	
20	Ctsc	P97821	Dipeptidyl peptidase 1	**-1.1**	**-1.05**	
21	Afm	O89020	Afamin	**-1.18**		**-0.53**
22	Gsn	P13020	Gelsolin	**-1.1**		**-0.16**
23	Hgfac	Q9R098	Hepatocyte growth factor activator	**-1.18**		**0.07**
24	Igfals	P70389	Insulin-like growth factor-binding protein	**-1.27**		**-0.43**

			complex acid labile subunit			
25	Itih1	Q61702	Inter-alpha-trypsin inhibitor heavy chain H1	**-1.14**		**-0.03**
26	Itih2	Q61703	Inter-alpha-trypsin inhibitor heavy chain H2	**-1.13**		**-0.26**
27	Itih4	A6X935	Inter alpha-trypsin inhibitor, heavy chain 4	**-1**		**-0.17**
28	Lum	P51885	Lumican	**-1.24**		**-0.26**
29	Psmb4	P99026	Proteasome subunit beta type-4	**-1.12**		**0.24**
30	Pi16	Q9ET66	Peptidase inhibitor 16	**-1.1**	**-0.67**	
31	Retn	Q99P87	Resistin	**-1.25**	**-0.73**	
32	Igfbp5	Q07079	Insulin-like growth factor-binding protein 5	**-1.14**	**-0.71**	

Red- Higher in males; **Blue**- Higher in Females; *-97% (C-statistics) discrimination between males and females (Mohammed et. al. 2021)

3. Some new frontiers of NP protein corona study should be cited. for example, top-down proteomics (PMID: 40764671 ; PMID: 39276099; PMID: 39312774) and AI (PMID: 40190726).

Response: Thank you for bringing these studies to our attention. We have now referenced these important recent advances in NP-protein corona research in the "Discussion" with appropriate context, page-14, line- 442:

"Looking ahead, advances such as top-down (69-71) and AI-driven computational modeling (72) hold promise for extending NP-based approaches by resolving intact proteoforms, characterizing post-translational modifications (PTMs), and improving mechanistic interpretation and predictive modeling of circulating biomarkers."

4. Based on my understanding, this study is not a longitudinal study because they studied different mice at three ages. The authors should clarify this point in the manuscript.

Response: This is correct and we agree that this should be made more explicitly clear that this study is not longitudinal despite utilizing samples from SLAM. Although the SLAM study itself is a longitudinal study, and the work presented here uses a cross-sectional subset of that cohort, different mice were sampled at 12, 24, and 30 months of age, rather than repeatedly sampling the same mice over time. We hope the multiple changes below will eliminate any ambiguity about the study design. That being made clear, we also intend to emphasize that a major benefit of the workflow developed here is that it makes a longitudinal proteomic biomarker study in mouse blood a possibility. By enabling comprehensive, quantitative proteomics in the very limited volumes available in a longitudinal mouse study, such as the 21µL available to us from SLAM, it is compatible with limitations of these study designs. We hope the changes quoted below clearly convey this information.

First, to make the study design clear, we refer to the cross-sectional design in the title of the manuscript, which now reads, “Deep and Quantitative Proteomic Profiling of Low Volume Mouse Serum: A Cross-Sectional Analysis of Aging”

We have made minor adjustments to the introduction to de-emphasize longitudinal studies and emphasize the low-volume compatibility as the underlying benefit for mouse studies of either cross sectional or longitudinal design.

We adjusted the results to introduce the samples as cross sectional: “We applied this method in a cross-sectional subset of 30 C57BL/6J mice from SLAM.”

We now explicitly describe in the “Study Design” section that the current analysis as cross-sectional and distinguish it from the broader longitudinal SLAM framework, page-15, line- 463:

“The serum proteomics reported here sample a cross-sectional subset of SLAM, in which different mice were sampled at each age rather than repeatedly sampling the same individuals over time. Accordingly, the age-associated patterns reflect between-group differences, not within-individual longitudinal trajectories.”

In addition, in limitations paragraph of “Discussion” we acknowledge that our design is cross-sectional, and that age-related signatures are inferred between distinct groups of mice rather than within individuals, which prevents assessment of true longitudinal trajectories over time. Although, in this study we developed nanoparticle-based enrichment and subsequent LC-MS methodology, which might be suitable for our longitudinal SLAM study in near future, page-14, line-424:

“Nonetheless, the NP-MS workflow demonstrated strong reproducibility and sensitivity, enabling robust detection of age- and sex--associated signatures. Importantly, this workflow was successful within strict sample volume requirements down to 10uL. Therefore, this method is compatible with the volume limitations of longitudinal mouse studies and can be easily adapted and scaled into large cohort studies with a longitudinal study design. Future phases of the SLAM, incorporating larger sample sizes and true longitudinal sampling will be essential to validate these associations, improve statistical power and generalizability, and more accurately capture temporal relationships and potential causal effects across the lifespan.”

5. A proteomics study of low-volume mouse serum samples using nanoparticle protein corona approach was published recently. The authors should cite and mention this paper. [PMID: 40726225]

Response: We thank the reviewer for bringing this recent study [PMID: 40726225] to our attention. We have referenced and mentioned it accordingly.

We incorporated this citation with appropriate context, page- 10, line- 309:
“The recent development of a high-throughput NP-protein corona enrichment in serum and plasma in the context of a mouse cancer model (42) underscores the growing utility of the strategy.”

Reviewer #2 (Remarks to the Author):

1. The reporting and application of false discovery rate (FDR) thresholds are inconsistent across the manuscript. For peptide and protein identification, a 1% FDR threshold is applied, and for age-associated proteins (linear modeling), the authors explicitly report FDR ≤ 0.05. However, for other analyses — including cytokine modeling, pairwise t-tests between age groups, and associations

with glucose and fat percentage — only nominal p-values are reported, with no mention of multiple testing correction. Given the high dimensional nature of proteomic and cytokine datasets, failure to consistently apply FDR inflates the risk of false positives and undermines the robustness of the reported associations. The authors must apply and report uniform FDR correction across all analyses. Without this, the reliability of many of the claimed findings is questionable.

Response: We thank the reviewer for careful evaluation and agree that consistent and transparent application and reporting of statistical cutoffs, including multiple- testing adjustment, is critical for rigor and reliability of the findings. As noted by the reviewer, we applied the standard combination of 1% FDR cutoff for proteomic IDs and FDR < 0.05 for relative quantification of aging changes by MS but were less stringent in the subsequent analysis. We resolved inconsistencies by standardizing the statistics in the main text and figures with a significance threshold of adjusted p-value < 0.05. Both p-values and adjusted p-values are now reported in the supplementary tables. We hope this provides adequate consistency and transparency in the application and presentation of statistics.

Regarding sample size, statistical power, and the robustness and reliability of claimed findings – we agree these are of critical importance and refer the reviewer to reviewer to the next response, which addresses this in more detail.

2. The biological conclusions are drawn from a relatively small cohort (n=30 mice, 10 per age group), which limits statistical power and generalizability. The cross-sectional design (different mice at each age) means that age-related trends are inferred between groups rather than within individuals. As the authors acknowledge, this precludes assessing individual longitudinal trajectories or causal relationships over time. While the data are intriguing, some findings (especially the finer details of non-linear changes or extensive lists of associated proteins) should be interpreted with caution due to the modest sample size

Response: We appreciate the reviewer's thoughtful comment and agree that it is important that the biological interpretations are not over-stated when the study is not powered to support them. To address these concerns, we have 1) performed additional PEA (O-link) proteomic assays for increased statistical power, 2) re-framed our claims to focus on the key (methodological) findings of the study, 3) adjusted language to mitigate longitudinal inference, and 4) substantially tempered or removed claims and figures related to modest trends and nominal statistics.

1) To increase statistical power of the PEA (O-link) proteomic assays, we performed additional experiments in age-matched mice from SLAM. The updated analysis of cytokine changes, **Fig. 3** and **Fig. 5b**, now reflect an increased sample size from n=30 (n=10 in each age group) to n=60 (n=20 in each age group). In the resulting analysis we identified 12 significant age-associated cytokines and 1 sex-associated cytokines (adjusted p-value < 0.05).

2) As the reviewer recognized, this study was not powered for identifying robust phenotypic changes and biomarker associations in SLAM. Instead, the study was designed to demonstrate a proteomic workflow that enables deep proteomic blood biomarker studies in mice in strictly volume limited conditions and showcase its application in the context of aging. The key strength/contribution of the study is that it introduces a method for *comprehensive* serum proteomic analysis in mouse cohorts, which are notably missing from existing literature due to the technical challenges associated with such studies. This workflow opens the door to comprehensive serum proteomic studies in mice even in volumes in low as 10 µL (11 µL for the addition of cytokines), a volume requirement that is compatible with longitudinal collection of blood in mice. This volume can also realistically be used for proteomics without consuming an entire serum volume and is thus compatible with studies that utilize blood for multiple different measurements (e.g. 'clinical' labs, proteomics, metabolomics, etc.). Although the current study is not powered to detect phenotypic changes, it is sufficiently powered to identify a larger number of age- and sex-associated proteins in serum than previously described (adjusted p-value < 0.05), reveal interesting aging biology, and potential clinical relevance, as evidenced by our addition of an additional analysis of a subset of protein changes that are conserved with humans (**Fig. 6**). In order to emphasize the strengths of the study in this framing, we have made the following adjustments:

page-14, line-424:

"Nonetheless, the NP-MS workflow demonstrated strong reproducibility and sensitivity, enabling robust detection of age- and sex--associated signatures. Importantly, this workflow was successful within strict sample volume requirements down to 10µL. Therefore, this method is compatible with the volume limitations of longitudinal mouse

studies and can be easily adapted and scaled into large cohort studies with a longitudinal study design. In future phases of the SLAM, incorporating larger sample sizes and true longitudinal sampling will be essential to validate these associations, improve statistical power and generalizability, and more accurately capture temporal relationships and potential causal effects across the lifespan."

We also completely agree that a larger study powered by the identification of aging-phenotype-associated biomarker signatures will be of ultimate interest. In fact, a separate, larger study powered for identification of phenotype-associated biomarker signatures is ongoing, and will ultimately demonstrate more detailed biomarker signatures, proteomic clocks, and multi-omic associations in a larger subset (n=900) of SLAM, with up to 6 longitudinal timepoints per animal. Because this is an ongoing, multi-team study that integrates multiple teams and datasets, it is outside the scope of the current manuscript. However, we have successfully completed the proteomic data acquisition for the larger SLAM study using the method described in this manuscript within 3 months, and with similar proteomic depth (>4000 cumulative proteins and ~2400 proteins per each 20ul serum sample), so we are confident in the scalability and applicability of the method described in this paper in a larger longitudinal mouse study.

To boost the significance of the current study, we would be happy to provide an additional figure summarizing the surface-level results of the larger SLAM study on request, including protein counts, list of protein/peptide identifications, and instrument time. These results would further demonstrate the scalability of this method and applicability in a longitudinal study for the current manuscript. However, given that the larger proteomic dataset is committed to an ongoing study, the underlying quantitative data from each sample would not be shareable at this time.

3) To reduce potential misunderstanding of current study might be longitudinal, we have changed wording in several places and have added paragraph into the discussion. Most notably, we have altered the title to highlight this as a cross-sectional analysis:

New Title: Deep and Quantitative Proteomic Profiling of Low Volume Mouse Serum: A Cross-Sectional Analysis of Aging

Discussion paragraph, page-14, line-424:

"Nonetheless, the NP-MS workflow demonstrated strong reproducibility and sensitivity, enabling robust detection of age- and sex--associated signatures. Importantly, this workflow was successful within strict sample volume requirements down to 10uL. Therefore, this method is compatible with the volume limitations of longitudinal mouse studies and can be easily adapted and scaled into large cohort studies with a longitudinal study design. Future phases of the SLAM, incorporating larger sample sizes and true longitudinal sampling will be essential to validate these associations, improve statistical power and generalizability, and more accurately capture temporal relationships and potential causal effects across the lifespan."

Introduction-page-4, line-89:

"the proteomic data analyzed here derive from a cross-sectional subset of SLAM, with different mice sampled at 12 (young), 24 (old), and 30 (geriatric) months. Our intention is therefore not to infer within-individual aging trajectories but rather evaluate whether deep, quantitative serum proteomics can be achieved in a sample-limited context relevant to longitudinal aging studies"

Discussion-page-11, line-323:

"However, because the proteomic data derives from a cross-sectional subset of the longitudinal SLAM cohort, age-associated signatures reflect population-level differences rather than within- individual aging trajectories. All biological interpretations are therefore framed conservatively; and the results should be viewed as foundational and hypothesis-generating rather than mechanistic or causal."

"Study Design" of the method section, page-15, line-463:

"The serum proteomics reported here sample a cross-sectional subset of SLAM, in which different mice were sampled at each age rather than repeatedly sampling the same individuals over time. Accordingly, the age-associated patterns reflect between-group differences, not within-individual longitudinal trajectories."

4) As requested, we have removed and tempered claims based on modest trends and nominal statistics. We agree that these data risk overstating our findings and detract from

the primary contributions of the study as detailed in #2 above. The figure panels (formerly Fig. 5) and text pertaining to body fat & glucose have been removed accordingly. Additionally, the analysis of pairwise changes between young, old, and geriatric age groups has been removed with the exception of the Gene Set Enrichment Analysis (**Fig. 4**) showing significant pathways based on adjusted p-values. We thank the reviewer for this suggestion and strongly believe it has improved the focus of the manuscript.

3. The linear modeling linking proteomic data to glucose and fat percentage has very weak explanatory power. For glucose, the model yielded $R^2 = 0.018$ with $P = 0.57$ — nonsignificant and essentially no predictive capacity. For fat percentage, the model yielded $R^2 = 0.248$ with $P = 0.03$, which is statistically significant but still explains only a modest fraction of the variability. These low R^2 values indicate that the proteomic profiles account for little of the observed differences in these phenotypes. The manuscript currently overstates the strength of these associations; claims that proteomic changes robustly predict metabolic traits should be substantially tempered or removed.

Response: We thank the reviewer for this suggestion. As indicated in the previous response in more detail, we agree and have removed this data accordingly.

4. The manuscript repeatedly frames the study as "longitudinal" and references the NIA SLAM cohort. For example, the Introduction emphasizes the need for longitudinal biomarker assessment, and the Discussion states that the workflow is compatible with "longitudinal investigations." However, the actual dataset presented here is strictly cross sectional — 30 different mice sampled at three ages (12, 24, 30 months), with no repeated measurements within the same animals. This is a critical distinction. Cross-sectional comparisons cannot capture within-individual trajectories, causal relationships, or true lifespan dynamics. Therefore, the current analysis does not justify claims of "longitudinal" insights into aging biology. The title and framing of the manuscript are misleading and should be corrected. Unless longitudinal data are added, the manuscript's contribution to lifespan biology is fundamentally limited.

Response: We appreciate the reviewer for this important clarification regarding the distinction between longitudinal and cross-sectional designs. We fully agree that the dataset analyzed and reported in this manuscript is strictly cross-sectional, comprising 30 different mice sampled at three ages (12, 24, and 30 months), with no repeated measurements from the same individuals.

As such, the study cannot provide within-animal aging trajectories, causal relationships, or true longitudinal insights.

To address this, we have implemented several changes detailed in our second response to you above:

To reduce potential misunderstanding of current study might be longitudinal, we have changed wording in several places and have added paragraph into the discussion. Most notably, we have altered the title to highlight this as a cross-sectional analysis:

New Title: Deep and Quantitative Proteomic Profiling of Low Volume Mouse Serum: A Cross-Sectional Analysis of Aging

Discussion paragraph, page-14, line-424:

"Nonetheless, the NP-MS workflow demonstrated strong reproducibility and sensitivity, enabling robust detection of age- and sex--associated signatures. Importantly, this workflow was successful within strict sample volume requirements down to 10uL. Therefore, this method is compatible with the volume limitations of longitudinal mouse studies and can be easily adapted and scaled into large cohort studies with a longitudinal study design. Future phases of the SLAM, incorporating larger sample sizes and true longitudinal sampling will be essential to validate these associations, improve statistical power and generalizability, and more accurately capture temporal relationships and potential causal effects across the lifespan."

Introduction-page-4, line-89:
"the proteomic data analyzed here derive from a cross-sectional subset of SLAM, with different mice sampled at 12 (young), 24 (old), and 30 (geriatric) months. Our intention is therefore not to infer within-individual aging trajectories but rather evaluate whether deep, quantitative serum proteomics can be achieved in a sample-limited context relevant to longitudinal aging studies"

Discussion-page-11, line-323:
"However, because the proteomic data derives from a cross-sectional subset of the longitudinal SLAM cohort, age-associated signatures reflect population-level differences rather than within-individual aging trajectories. All biological interpretations are therefore framed conservatively; and

the results should be viewed as foundational and hypothesis-generating rather than mechanistic or causal."

"Study Design" of the method section, page-15, line-463:
"The serum proteomics reported here sample a cross-sectional subset of SLAM, in which different mice were sampled at each age rather than repeatedly sampling the same individuals over time. Accordingly, the age-associated patterns reflect between-group differences, not within-individual longitudinal trajectories."

We now explicitly state that age-associated findings represent between group comparisons and should be interpreted accordingly. The manuscript also clarifies that the primary purpose of this study is to establish and benchmark a scalable NP-based proteomics workflow that will enable future longitudinal analyses when serial sample become available in subsequent phases of SLAM. Overall, we hope these revisions ensure accurate framing of the study and eliminate any ambiguity regarding the cross-sectional nature of the dataset.

5. One critical challenge associated with the use of NP-based strategies for biomarker discovery is the often observed lack of direct correlation between the proteins identified in the NP protein corona and those present in the plasma proteome. This discrepancy means that, even if proteomic variations are detected within the protein corona, additional validation using complementary analytical techniques [such as enzyme-linked immunosorbent assay (ELISA)] is essential to confirm the biological relevance and accuracy of these findings. Relying solely on proteomics data from the corona without validation can lead to misleading conclusions about potential biomarkers. Furthermore, it is imperative to consider the impact of aging on plasma composition, particularly regarding lipids, metabolites, and other small molecules. These factors can significantly influence the formation and composition of the protein corona on NPs, often more so than the plasma proteome itself. Unlike traditional proteomics methods, even minor alterations in lipid profiles or metabolic constituents can substantially modify the corona's molecular decoration, subsequently affecting proteomic outcomes without any apparent change in the plasma's overall protein composition. This dynamic interplay has been extensively documented in current literature, emphasizing that lipids and metabolites can modulate the affinity, binding patterns, and the overall structure of the corona, thereby influencing the detectability and interpretability of biomarkers.

Response: This is an important point, and we appreciate the importance of considering potential confounding effects on the composition of the protein corona, and the critical need to demonstrate the quantitative aging changes derived from the protein corona are representative of differences in the original serum samples. We have addressed and acknowledged these concerns with the inclusion of additional analysis and discussion. We hope the combination of responses presented below sufficiently address the underlying concerns of the reviewer.

First, we contend that the 'gold standard' method to demonstrate whether changes in the protein corona are representative of 'real' changes in the original serum samples at the proteome level (across a wide range of proteins) is to perform quantitative benchmarking (PMID: 27701404) on the same nanoparticles utilized in our study. This concern motivated us to conduct our own independent benchmarking study, shown in **Fig. 2A-B** (see below). Given that human and bovine blood are distinct in their compositions (including metabolites and lipids), if lipids/metabolites had a significant confounding effect on the composition of the protein corona, our expectation would be that our observed protein ratios would be skewed and inaccurate. However, our data suggests that on average the quantitative ratios of proteins in the protein corona are representative of true ratios of the original blood samples across a range of dilutions (5% - 100%).

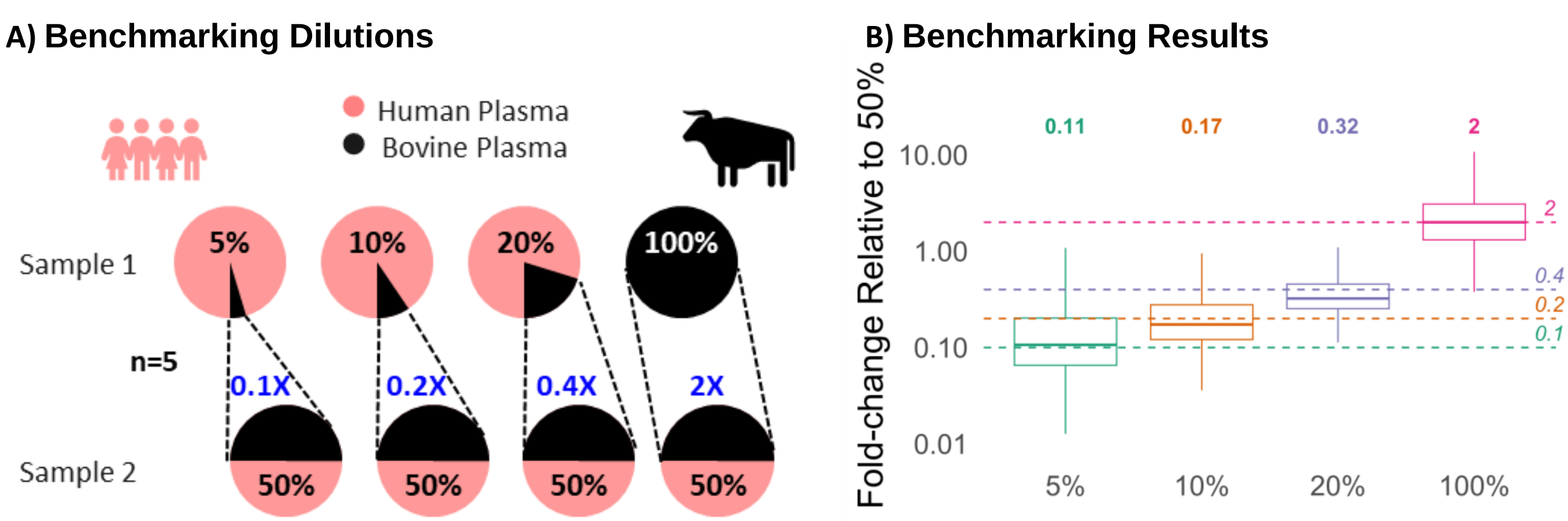

"Figure 2: Qualitative and quantitative evaluation of proteomic data generated NP-coupled MS workflow. A) A quantitative benchmarking study was performed using five fixed ratios of human and bovine plasma (n=5 each). B) Boxplot showing the measured peptide ratios in each dilution versus the 50% bovine plasma sample. Dotted lines depict the expected ratios."

Second, we emphasize that published literature, which has more rigorously and comprehensively examined characteristics of the protein corona and binding properties of the nanoparticles utilized in this study, suggests that the effect of lipids and matrix do not significantly impact quantitation. We note that the nanoparticles used in this study are distinct from the plain polystyrene nanoparticles in the study cited by Reviewer #1 (PMID: 39511193) and may not exhibit the same binding behavior. The surface-functionalized superparamagnetic iron nanoparticles in this study are characterized in more detail elsewhere (PMID: 35275789). Lipid spike-in experiments have been done on the same nanoparticles we used, showing that spiking varying amounts of lipid do not significantly affect the number/intensity of proteins (PMID: 32699280, Nature Comms - last paragraph of p5 & first on p6, Fig. S4 and S5). Another paper in JPR (PMID: 41105214) reported on the minimal impact of background matrix (presumably with different lipid/metabolite compositions) on quantitative accuracy. We have noted these additional studies in the discussion (quoted below).

We explained now in the revised “Discussion” section for more clarity, page-10, line-295:

“Concerns have been raised that NP- coronas may be sensitive to non-protein serum components, such as lips or metabolites, potentially introducing variability unrelated to biological differences. While recent work has shown that small molecules can influence protein corona composition derived from plain polystyrene nanoparticles (40), multiple independent studies of the surface-functionalized superparamagnetic iron nanoparticles employed in this study suggest that these effects are not significant. Lipid-spike-in experiments using the same nanoparticles showed no appreciable changes in corona protein identities or intensities (15). Similarly, a prior report suggests minimal impact of background matrix on quantitative accuracy (41). Our own quantitative benchmarking data further supports this conclusion, showing high reproducibility and accurate quantification of known fold-changes in samples generated from mixing human and bovine blood, each containing a distinct composition of lipids, metabolites, and other components, in a range of fixed dilutions spanning 5% to 100% (**Fig. 2B**). Together, these findings indicate that matrix-derived components did not significantly alter corona composition in a manner that distorts relative protein quantification in this study and mitigate concerns matrix effects introduced variability unrelated to aging.”

Third, we attempted to address the reviewer’s request for independent validation of the aging changes measured in protein coronas with additional analysis and comparison with literature.

Targeted cytokine measurements with the Proximity Extension Assay, and antibody-affinity-based platform, were a major component of this study. To perform a cross-platform comparison of a protein measured by both methods, nanoparticle-based MS versus antibody-based, we searched for overlapping protein observations between datasets and found a single protein – IL16 – with enough observations across mice to perform a comparison (**Fig. 3C-D**, 11 mice with matched measurements in both platforms). Using linear modeling, we found significant correlation between platforms in the measurement of IL16 ($p=0.0026$, $R = 0.81$). This example further supports our claim of accurate representation of protein levels in the protein corona when compared with the original serum samples. To further validate whether our observed changes align with expected biology and proteomic measurements from prior studies, we performed an analysis comparing our measured changes to two published MS-based proteomic datasets in mouse blood that did not use nanoparticles. Although there are no prior mouse aging studies in blood to draw from, the two published studies examined sex-associated changes in mouse serum (PMIDs 37910662 and 34050187). Notably, with the exception of two proteins out of thirty-two (Transcobalamin-2 and Hepatocyte growth factor activator), all sex-associated proteins from our study that were also reported in either published study were directionally aligned and correlated. This has been added as a supporting figure, **Fig. S3**, and is numerically detailed in **Table S4**. Overall, these findings further support that protein corona measurements in our study align with direct measurements in blood samples and accurately reproduce expected biological changes, such as sex-dimorphism.

New figure, **Fig. S3**:

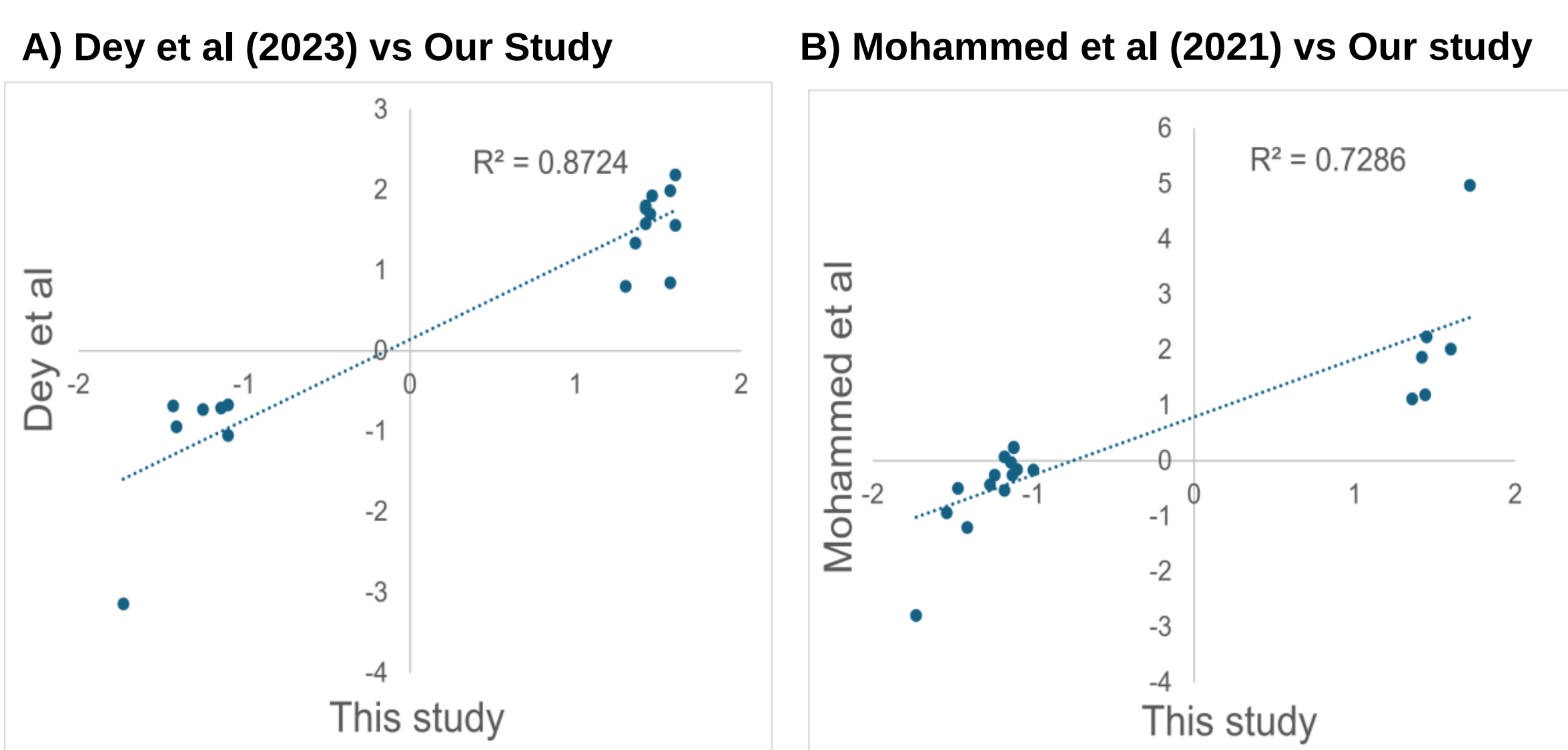

“**Supplementary Fig 3. Correlation of sex-associated circulating proteins between this study and published datasets.** Correlation plots depicting sex-associated changes in our study versus those reported in A) Dey et. al. (PMID: 37910662); and B) Mohammed et. al. (PMID- 34050187). Proteins in both studies show strong positive correlations with this study (R^2=0.8724 and R^2=0.7286, respectively), and consistent directionality of sex associated effects.”

Supplemental Table 4: Validation of top sex associated proteins with other study

				**SLAM**	**Dey et. al. JPR,2023**	**Mohammed et. al. NPJ Syst. Biol. Appl. 2021**
**Sl. No.**	**Gene**	**Uniprot ID**	**Description**	**B-sex**	**log2(M/F)**	**log2(M/F)**
1	Serpina1e*	Q00898	Alpha-1-antitrypsin	**1.72**		**4.97**
2	C8g*	Q8VCG4	Complement component C8 gamma chain	**1.6**	**1.56**	**2.02**
3	C6	E9Q6D8	Complement component 6	**1.6**	**2.19**	
4	C7	D3YXF5	Complement component 7	**1.57**	**1.99**	
5	C8b*	Q8BH35	Complement component C8 beta chain	**1.45**	**1.7**	**2.24**
6	C8a*	Q8K182	Complement component C8 alpha chain	**1.42**	**1.77**	**1.87**
7	Ces3a	Q63880	Carboxylesterase 3A	**1.57**	**0.85**	
8	Mug1*	P28665	Murinoglobulin-1	**1.44**		**1.19**
9	Mup20	Q5FW60	Major urinary protein 20	**1.46**	**1.93**	
10	Crisp1	Q03401	Cysteine-rich secretory protein 1	**1.42**	**1.8**	

11	Mup3	P04939	Major urinary protein 3	1.42	1.58	
12	Egfr*	Q01279	Epidermal growth factor receptor	1.36	1.34	1.12
13	Serpina3k	P07759	Serine protease inhibitor A3K	1.3	0.8	
14	Tcn2	O88968	Transcobalamin-2	-1.12		0.25
15	Glycam1*	Q02596	Glycosylation-dependent cell adhesion molecule 1	-1.73	-3.14	-2.79
16	Adipoq*	Q60994	Adiponectin	-1.54		-0.94
17	Bche	Q03311	Cholinesterase	-1.47		-0.5
18	Serpina6*	Q06770	Corticosteroid-binding globulin	-1.41	-0.94	-1.2
19	Scg3	P47867	Secretogranin-3	-1.43	-0.68	
20	Ctsc	P97821	Dipeptidyl peptidase 1	-1.1	-1.05	
21	Afm	O89020	Afamin	-1.18		-0.53
22	Gsn	P13020	Gelsolin	-1.1		-0.16
23	Hgfac	Q9R098	Hepatocyte growth factor activator	-1.18		0.07
24	Igfals	P70389	Insulin-like growth factor-binding protein complex acid labile subunit	-1.27		-0.43
25	Itih1	Q61702	Inter-alpha-trypsin inhibitor heavy chain H1	-1.14		-0.03
26	Itih2	Q61703	Inter-alpha-trypsin inhibitor heavy chain H2	-1.13		-0.26
27	Itih4	A6X935	Inter alpha-trypsin inhibitor, heavy chain 4	-1		-0.17

28	Lum	P51885	Lumican	**-1.24**		**-0.26**
29	Psmb4	P99026	Proteasome subunit beta type-4	**-1.12**		**0.24**
30	Pi16	Q9ET66	Peptidase inhibitor 16	**-1.1**	**-0.67**	
31	Retn	Q99P87	Resistin	**-1.25**	**-0.73**	
32	Igfbp5	Q07079	Insulin-like growth factor-binding protein 5	**-1.14**	**-0.71**	

Red- Higher in males; **Blue**- Higher in Females; *-97% (C-statistics) discrimination between males and females (Mohammed et. al. 2021)

6. The addition of the 48-plex cytokine panel is a great complement to the MS data, given MS's difficulty in detecting many cytokines. However, the cytokine panel is limited to the targets included in that assay, meaning some relevant inflammatory markers could still be missing if not on the panel. The overlap between MS and PEA was small (only 7 proteins detected by both, according to the text), indicating that each method captures distinct subsets of the proteome. This underscores the importance of using both; however, the data from the two platforms are largely presented separately. Recommendation: The authors could consider a more integrated analysis or discussion of how the two datasets together inform the biological story. For instance, do the cytokines uniquely detected by PEA show any coherent pattern with age or sex (some of this is touched on in Fig.3 and Fig.5B)? Highlighting any such insights would further demonstrate the value of the combined approach. Additionally, to enhance clarity, the manuscript should clearly state the identity of the cytokine panel (e.g. PEA technology by a specific provider) and confirm if results from the two methods were normalized or analyzed independently. Minor point: including a brief mention of any quality control for the PEA assay (e.g. duplicates, standard curves, or limit-of-detection considerations) would be reassuring.

Response: We thank the reviewer for thoughtful and constructive feedback regarding the complementary cytokine panel and the opportunity to more fully integrate insights from the MS and proximity extension assay (PEA) datasets. We agree that MS and PEA capture distinct but biologically meaningful subsets of the circulating proteome and that emphasizing their combined

interpretive value strengthens the manuscript. The small overlap between MS and PEA (4 proteins) resulting in complementary views of the circulating proteome that includes many important, but not all, cytokines that are traditionally missed by MS.

We appreciate the reviewer's recommendation to further integrate the two proteomic datasets and agree it would further highlight their complementarity. In a newly introduced figure that assesses the overlap of these findings with aging changes in human blood (**Fig. 6**), we have integrated our analysis to include both datasets. Of the 18 age-associated proteins conserved in humans, 2 were from PEA and 16 were from MS (**Fig. 6A,B,D**). Given the small number of significant changes by PEA for the 30 mouse samples that have matched analysis in both PEA and MS (four proteins adjusted p-value < 0.05), we are somewhat limited in the additional insight that can be gained from integration. At the level of biology, we view PEA cytokine panel as an independent targeted analysis of the inflammatory state of the organism that the MS dataset does not necessarily reflect due, but due to the targeted nature of the assay, there are some limitations to the integration (e.g. integrated pathway analysis, since the background protein list is entirely a single pathway - an inflammatory cytokine panel). However, we modified the discussion to include integrated speculation/insights and discuss integration of data types. page-11, line343:

"Although overlap between MS and PEA was limited (4 proteins, **Fig. 3C**), reflecting the fundamentally different detection principles and sensitivity ranges of the two platforms. However, cytokines uniquely detected by PEA exhibited coherent age-associated patterns including increases in 10 cytokines, including known SASP factors Il6, Tnf, Csf3, Csf1, and Ccl2. Together with the observed increases in SenSig proteins and immune cell activation pathways by MS-based proteomics, these results support an age-related increase in senescence-driven immune cell activation in circulation. The PEA panel provides an independent assessment of the inflammatory state of the organism based on inflammatory cytokines that are missed by traditional unbiased MS assays."

To enhance clarity, we now explicitly state in the "Methods" that cytokines were quantified using Olink Target 48 Mouse Cytokine Panel, which is based on Proximity Extension Assay (PEA) technology. We also clarify that PEA and MS datasets were processed and normalized independently using their respective platform-specific pipelines, reflecting differences in dynamic range, detection chemistry, and quantification scales. Finally, we have added a brief description of PEA quality control procedures, page-18, line-558:

“Cytokines in the mouse serum samples were quantified using the Olink Target 48 Mouse Cytokine panel based on Proximity Extension Assay (PEA) technology (Olink Proteomics. Assays were performed by Assay Gate Inc. (Ijamsville, MD) according to manufacturer’s instructions. PEA and MS datasets were processed and normalized independently using platform-specific pipelines due to differences in assay chemistry and dynamic range. Quality control assessments of the PEA assay include inclusion of process controls (for key protocol steps – incubation, extension, and detection), a triplicate sample control to monitor intra-run precision, duplicate negative controls, and triplicate calibrator samples for normalization between batches (77).The limits of detection and quantification, and precision of each protein measurement have been previously reported (77).” All samples used for this study passed internal and external quality control metrics.

Some major questions that need to be addressed:

1) Why emphasize MaxRep when MaxLFQ is more established and less biased? Can the authors provide statistical evidence for superior reproducibility?

Response: We appreciate the reviewer’s question regarding our emphasis on the MaxRep roll-up approach. We believe both approaches are valid, but in our hands the MaxRep approach worked marginally better. MaxRep is based on empirical performance in this specific NP-fractionated workflow, where each peptide is quantified using its highest intensity peptide–nanoparticle observations, and was widely adopted as the default quantification approach for these nanoparticle kits until recently. However, as the reviewer points out, MaxLFQ is indeed a widely used and well-established method for peptide-to-protein quantification in traditional MS studies, and for this reason we compare both MaxRep and MaxLFQ directly within our dataset in the context of an NP-based workflow.

In our comparison of each approach, we quantified similar number of proteins (3992 proteins) in each. However, in our analysis of reproducibility based on the CVs of protein-level quantification (**Fig. 2J**), MaxRep demonstrated slightly superior reproducibility, with a lower median protein-level coefficient of variation (CV = 0.0567) compared to MaxLFQ (CV = 0.0613). MaxRep also yielded a modestly higher proportion of proteins with CV < 10%, reflecting tighter quantitative precision under our experimental conditions. These results imply that MaxRep can marginally reduce cross-nanoparticle variability by using the most consistently observed and highest-

intensity peptide measurement for each protein. Importantly, the quantitative accuracy of the MaxRep approach was already corroborated in our quantitative benchmarking study (**Fig. 2B**) before the MaxRep/MaxLFQ comparisons were performed.

To reflect the reviewer's concern about potential bias, we note in the revised text, page-7, line-193:

"Accordingly, MaxRep was used for primary analyses due to its marginally improved reproducibility, while downstream biological conclusions were robust to roll-up choice."

2) The study identifies >70 age-associated proteins. Which of these are validated against prior murine or human aging proteomics datasets?

Response: We appreciate the suggestion and agree that comparison with large-scale human and mouse proteomics datasets is important to establish confidence in the data and that mouse serum proteomics can inform human aging biology. With regard to mouse data – there are no comprehensive proteomic studies of aging to draw from to our knowledge, but several published MS studies did evaluate sex-specific protein changes in mouse serum that we could use to assess whether our study reproduces 'known' biology. We discuss these comparisons in detail in the next response to your question about comparing sex-dimorphic signatures with existing literature.

To address the comparison with existing human aging data, the revised manuscript incorporates a cross- species comparison between the mouse proteomic signatures identified in this study and human plasma proteomics from Baltimore Longitudinal Study of Aging (BLSA) and published UK Biobank proteomics that support the translational relevance of the mouse data.

The newly generated analysis has been included in the results as a main figure (**Fig. 6**). Our analysis reveals that 50% of the age-associated proteins identified in this mouse study are also significantly age-associated in the BLSA. Interestingly, these results suggest a common circulating aging signature of extracellular matrix, endoplasmic reticulum, and lysosomal protein components in humans and mice. We assessed the association of these proteins with frailty, a key clinical aging endpoint, in BLSA and found that five significantly associated with frailty: COL6A3, LBP, PIGR, IGFBP1 and HPX. By far, the ECM protein COL6A3 is the most significant among these (adj. p-value = 5.07e-16). These findings are consistent with recent evidence of age-associated increase in ECM components in Nature Aging (PMID: 41023316), and a broad range

of literature associating COL6A3 (aka Endotrophin, the uncleaved precursor) with mortality and chronic diseases (PMID 38871093). Notably, COL6A3 is among the top 5 proteins associated with pre-frailty, and frailty, in the UK Biobank (PMID 39535692), independently validating the association with human frailty observed in BLSA. Overall, these results support clinical relevance of the findings of our study and demonstrate the mouse study can shed light on human aging. We have included the new analysis into the results, figures, and discussion, accordingly, as shown below.

New Figure, **Fig. 6**:

New analysis described in the Results section, page-9, Line-253:

"To assess cross-species concordance, we compare age-associated circulating proteins in mice with human plasma proteomics from the BLSA (35, 36). Among 1742 proteins quantified in both datasets, Pearson Linear modeling (age ~ protein + sex) identified 18 proteins, representing 5% of the total proteins or 50% of mouse age-associated proteins, to be significantly (**Fig. 6A**) and positively (Fig. 6B) associate with age in both mice and humans. Overrepresentation analysis of shared upregulated proteins with age in both mice and humans revealed enrichment for cellular component categories (Fig. 6C) including endoplasmic reticulum, extracellular matrix, and lysosomal proteins, among others, and suggesting shared categories of age- associated circulating factors at a pathway-level. To assess the relevance of these findings with age-associated clinical phenotypes, we evaluated the associations of these proteins with frailty index, a 44-component composite score, in the BLSA. Notably, five proteins including COL6A3, LBP, PIGR, and IGFBP1, and HPX were significantly associated with frailty index in the BLSA (Frailty Index ~ Protein + age + sex; **Fig. 6D**). Collectively, these results link conserved proteomic aging signatures to functional health decline in humans and suggest that aging-associated biomarkers derived from mice may be relevant in humans."

We have emphasized these cross-species findings in the Abstract, page-2:

"Cross-species comparison with human plasma proteomics from the Baltimore Longitudinal Study of Aging (BLSA) identified conserved age-associated circulating proteins, several of which were linked to frailty in humans."

Finally, we elaborated on the significance of these results in the Discussion, page-13, line 394:

"To place the mouse findings in a translational context, we compared age-associated circulating proteins identified here with human plasma proteomics from BLSA. Despite differences in species, cohort design, and analytical platforms, a subset of proteins, representing half of the mouse age-associated proteins, were concordantly age- associated in humans (Fig. 6A, 6B), indicating conserved features of the circulating aging proteome. Interestingly, collagen-containing extracellular matrix proteins were overrepresented among these, consistent with recent reports of the association of extracellular matrix components, including collagen, with aging in mouse circulation (66). Moreover, several of these shared proteins, including COL6A3, LBP, PIGR, IGFBP1, and HPX, were also significantly associated with frailty in humans, linking shared circulating proteomic signatures to functional health decline. Notable among these, Collagen Type VI Alpha 3 Chain (COL6A3, a.k.a. Endotrophin), a well-known indicator of fibrotic aging, was by far the most significant frailty-associated protein in the BLSA (adjusted p-value = 5.069e-16). COL6A3 has been associated with mortality and a range of non-communicable chronic diseases linked to aging, including idiopathic pulmonary fibrosis, chronic kidney disease, type 2 diabetes, cardiac dysfunction, among others (67). Remarkably, COL6A3 is also among the top five proteins associated with pre-frailty and frailty in the UK Biobank (68), independently validating the robustness of this finding in humans. Although exploratory, these findings collectively support the translational relevance of mouse serum proteomics and suggest that at least a subset of aging-associated circulating proteins is conserved in humans. While we expect the correlation of mouse and human age-associated proteins as a whole to be limited due to the substantial influence of genetics and other factors on the circulating proteome, we speculate that a key subset of circulating proteins with mechanistic links to biology will be conserved and translationally relevant. Notable among these in our study are robust aging- and disease-associated proteins such as COL6A3 and lipopolysaccharide-binding protein (LBP), and sex-associated proteins such as Complement 8 (conserved association with the male sex) and adiponectin (ADIPOQ, conserved association with females)."

3) How do the sex-dimorphic signatures align with existing literature? Are these findings reproducible in independent datasets?

Response: We appreciate the reviewer's question regarding the biological validity and reproducibility of the sex-dimorphic protein signatures. To further validate whether our observed changes align with expected biology and proteomic measurements from prior studies, we

performed an analysis comparing our measured changes to two published MS-based proteomic datasets in mouse blood that did not use nanoparticles. Although there are no prior mouse aging studies in blood to draw from, the two published studies examined sex-associated changes in mouse serum (PMIDs 37910662 and 34050187). Notably, apart from two proteins (Transcobalamin-2 and Hepatocyte growth factor activator) out of thirty-two, all sex-associated proteins from our study that were also reported in either published study were directionally aligned and correlated. This has been added as a supporting figure, **Fig. S3**, and is numerically detailed in Table S4. Overall, these findings further support that protein corona measurements in our study align with direct measurements in blood samples and accurately reproduce expected biological changes, such as sex-dimorphism.

New figure, **Fig. S3**:

A) Dey et al (2023) vs Our Study **B) Mohammed et al (2021) vs Our study**

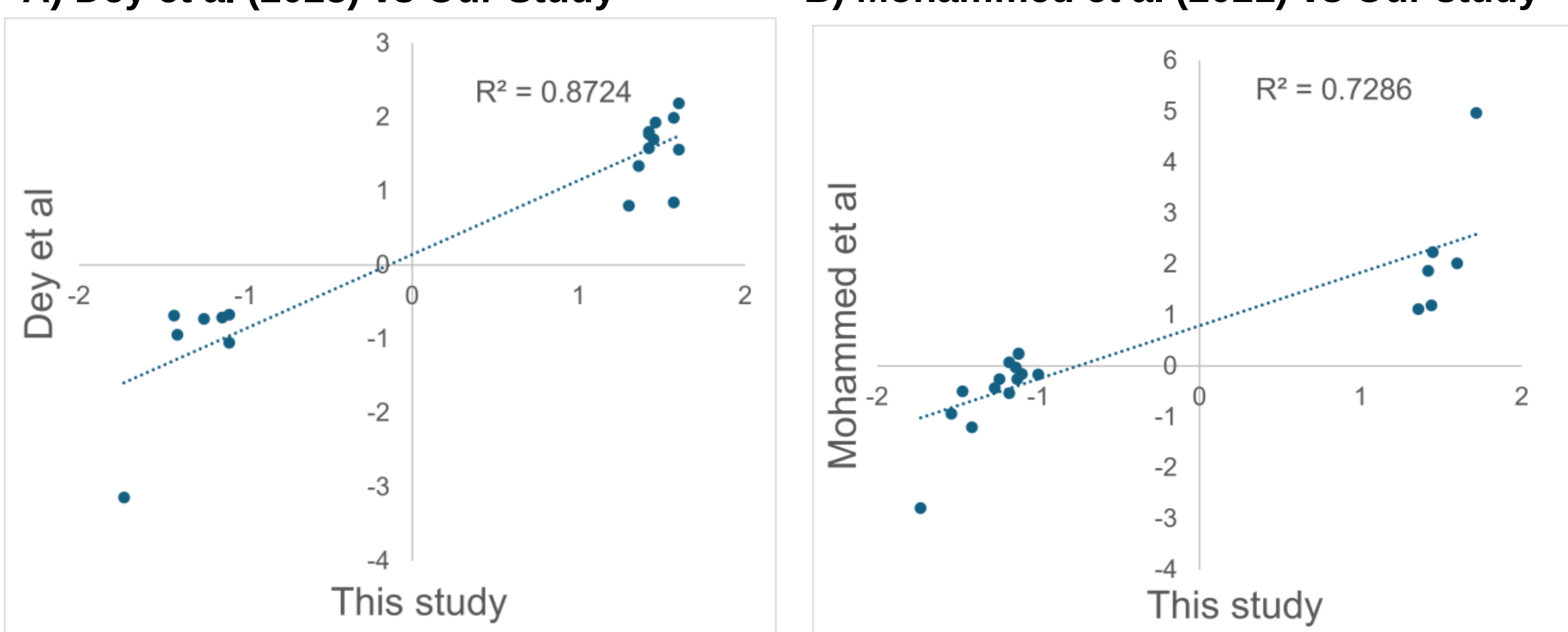

"Supplementary Fig. 3- Correlation of sex-associated circulating proteins between this study and published datasets. Correlation plots depicting sex-associated changes in our study versus those reported in A) Dey et. al. (PMID: 37910662); and B) Mohammed et. al. (PMID- 34050187). Proteins in both studies show strong positive correlations with this study (R^2=0.8724 and R^2=0.7286, respectively), and consistent directionality of sex associated effects."

Supplemental Table 4: Validation of top sex associated proteins with other study

	**SLAM**	**Dey et. al. JPR,2023**	**Mohammed et. al. NPJ Syst.**

Sl. No.	Gene	Uniprot ID	Description	B-sex	log2(M/F)	Biol. Appl. 2021 log2(M/F)
1	Serpina1e*	Q00898	Alpha-1-antitrypsin	**1.72**		**4.97**
2	C8g*	Q8VCG4	Complement component C8 gamma chain	**1.6**	**1.56**	**2.02**
3	C6	E9Q6D8	Complement component 6	**1.6**	**2.19**	
4	C7	D3YXF5	Complement component 7	**1.57**	**1.99**	
5	C8b*	Q8BH35	Complement component C8 beta chain	**1.45**	**1.7**	**2.24**
6	C8a*	Q8K182	Complement component C8 alpha chain	**1.42**	**1.77**	**1.87**
7	Ces3a	Q63880	Carboxylesterase 3A	**1.57**	**0.85**	
8	Mug1*	P28665	Murinoglobulin-1	**1.44**		**1.19**
9	Mup20	Q5FW60	Major urinary protein 20	**1.46**	**1.93**	
10	Crisp1	Q03401	Cysteine-rich secretory protein 1	**1.42**	**1.8**	
11	Mup3	P04939	Major urinary protein 3	**1.42**	**1.58**	
12	Egfr*	Q01279	Epidermal growth factor receptor	**1.36**	**1.34**	**1.12**
13	Serpina3k	P07759	Serine protease inhibitor A3K	**1.3**	**0.8**	
14	Tcn2	O88968	Transcobalamin-2	**-1.12**		**0.25**
15	Glycam1*	Q02596	Glycosylation-dependent cell adhesion molecule 1	**-1.73**	**-3.14**	**-2.79**
16	Adipoq*	Q60994	Adiponectin	**-1.54**		**-0.94**

17	Bche	Q03311	Cholinesterase	**-1.47**		**-0.5**
18	Serpina6*	Q06770	Corticosteroid-binding globulin	**-1.41**	**-0.94**	**-1.2**
19	Scg3	P47867	Secretogranin-3	**-1.43**	**-0.68**	
20	Ctsc	P97821	Dipeptidyl peptidase 1	**-1.1**	**-1.05**	
21	Afm	O89020	Afamin	**-1.18**		**-0.53**
22	Gsn	P13020	Gelsolin	**-1.1**		**-0.16**
23	Hgfac	Q9R098	Hepatocyte growth factor activator	**-1.18**		**0.07**
24	Igfals	P70389	Insulin-like growth factor-binding protein complex acid labile subunit	**-1.27**		**-0.43**
25	Itih1	Q61702	Inter-alpha-trypsin inhibitor heavy chain H1	**-1.14**		**-0.03**
26	Itih2	Q61703	Inter-alpha-trypsin inhibitor heavy chain H2	**-1.13**		**-0.26**
27	Itih4	A6X935	Inter alpha-trypsin inhibitor, heavy chain 4	**-1**		**-0.17**
28	Lum	P51885	Lumican	**-1.24**		**-0.26**
29	Psmb4	P99026	Proteasome subunit beta type-4	**-1.12**		**0.24**
30	Pi16	Q9ET66	Peptidase inhibitor 16	**-1.1**	**-0.67**	
31	Retn	Q99P87	Resistin	**-1.25**	**-0.73**	
32	Igfbp5	Q07079	Insulin-like growth factor-binding protein 5	**-1.14**	**-0.71**	

Red- Higher in males; **Blue**- Higher in Females; *-97% (C-statistics) discrimination between males and females (Mohammed et. al. 2021)

The convergence with multiple external datasets provides confidence that the identified sex differences reflect true, biologically conserved features of the circulating proteome rather than cohort- or method-specific artifacts.

4) Cytokine panel results show limited overlap with MS. Could this reflect assay limitations, matrix effects, or biological absence? Provide a critical evaluation.

Response: We appreciate the reviewer's insightful question regarding the limited overlap between cytokines detected by MS and those quantified by the PEA cytokine panel. This discrepancy is expected and reflects a combination of biological, technical, and matrix-dependent factors inherent to each platform. In MS proteomics, it is known that inflammatory cytokines are commonly missed due to a lack of unique tryptic peptides with adequate ionization efficiency for detection, so we expected these measurements to be lacking in our MS analysis. This was our primary motivation for selecting a targeted mouse cytokine panel, so our results were in line with this expectation. In matrices such as blood, as the reviewer points out, this challenge is further compounded because the abundance of cytokines tends to be at the bottom end of the vast dynamic range of protein concentrations spanning 10+ orders of magnitude, thus we reasoned that a highly sensitive affinity-based based approach with a demonstrated capability to detect these proteins in blood (PEA/Olink) would be an ideal complimentary assay.

In the "Introduction" section of the manuscript (page-3, line- 74) we discuss that MS-based detection of cytokines is constrained by small protein size, limited tryptic peptides, and poor ionization efficiency, whereas PEA directly targets proteins with high analytical sensitivity and minimal matrix interference. Conversely, PEA is inherently limited to the 48 predefined targets included on the panel; cytokines or inflammatory mediators not represented cannot be detected.

In the revised manuscript, we highlight that the two platforms together provide a more complete view of the circulating inflammatory landscape than either could achieve alone- page-11, line-343:

"Although overlap between MS and PEA was limited (4 proteins, **Fig. 3C**), reflecting the fundamentally different detection principles and sensitivity ranges of the two platforms. However, cytokines uniquely detected by PEA exhibited coherent age-associated patterns including increases in 10 cytokines, including known SASP factors Il6, Tnf, Csf3, Csf1, and Ccl2. Together with the observed increases in SenSig proteins and immune cell activation pathways by MS-

based proteomics, these results support an age-related increase in senescence-driven immune cell activation in circulation. The PEA panel provides an independent assessment of the inflammatory state of the organism based on inflammatory cytokines that are missed by traditional unbiased MS assays."

5) Several biological conclusions (e.g., links to metabolic health) are speculative. Can authors provide mechanistic validation or stronger evidence?

Response: We agree that data related to metabolic outcomes (e.g. glucose/fat associated proteins) were limited. In line with your earlier suggestions to temper or remove the statistically marginal results, we removed much of the analysis and re-focused the manuscript on the strengths of the study. This is described in more detail in our response to your second 'main' comment. Our discussion now focuses more on the advantages of this proteomic workflow and more statistically definitive results, such as the age-and sex-associated proteins.

6) What measures were taken to ensure randomization and avoid bias in sample preparation and acquisition?

Response: Several measures were implemented to ensure that sample processing and MS acquisition were not confounded by batch effects or systematic bias.

a) **Sample processing:** To minimize batch effects and procedural bias, serum samples were block-randomized or randomized under the constraint that samples of each age- and sex- are equally distributed across 96-well plates on the automated SP100 platform. Each plate/batch contains process controls to account for technical variability and system suitability, as well as sample controls to assess inter-plate performance. The automated sample processing is performed on one plate at a time, where samples are treated in parallel with the same five distinct NPs under identical incubation and workflow conditions, ensuring uniform handling and minimizing systematic technical variation during protein corona formation, digestion, and peptide cleanup. This randomized and balanced processing strategy has now been described in the Methods section of revised manuscript- page-16, line-489:

"All serum samples were processed using the Seer Proteograph workflow on an automated SP100 instrument [15, 29], employing a randomized and age- and sex-balanced 96- well plate design to minimize batch effects and procedural bias."

Page-16, line-507:

"All samples were processed under identical workflow conditions using an automated workflow, ensuring uniform handling and minimizing technical variability during protein corona formation, digestion, and peptide clean-up.

b) **Block-randomized LC–MS acquisition:** As noted in the Methods, DIA-MS runs were acquired in a block-randomized order (already included in the method section- page-17, line-536), which distributes samples from each age and sex group across the acquisition sequence and minimizes potential bias related to instrument drift, carryover, or temporal variation in MS performance.

c) **Use of technical replicates in feasibility and quantitative benchmarking:** For the NP reproducibility and fold accuracy benchmarking experiment, technical triplicates were processed and acquired independently, confirming low CVs and limiting operator- or batch-related bias.

Together, these steps ensured that sample preparation and acquisition were unbiased with respect to age and sex groups, and that downstream quantitative comparisons reflected biological rather than procedural differences.

7) Given the small sample size, what is the risk of false discovery? How do the authors plan to validate these biomarkers in larger cohorts?

Response: We agree that modest sample size increases the risk of false discovery, particularly for subtle or non-linear age-associated changes. To mitigate this, we applied stringent statistical controls, including peptide/protein FDR at a 1%, and we interpreted all findings as hypothesis-generating rather than definitive. As outlined in the revised manuscript, candidate biomarkers identified will be validated in larger cohorts through upcoming phases of the SLAM study, which will include substantially greater mouse numbers and true longitudinal sampling, as well as

through targeted assays such as ELISA, multiplex immunoassays, or targeted MS to confirm reproducibility and biological relevance.

As noted above, a separate, larger study powered for identification of phenotype-associated biomarker signatures is ongoing, and will ultimately demonstrate more detailed biomarker signatures, proteomic clocks, and multi-omic associations in a larger subset (n=900) of SLAM, with up to 6 longitudinal timepoints per animal. Because this is an ongoing, multi-team study that integrates multiple teams and datasets, it is outside the scope of the current manuscript. However, we are happy to share that the proteomic data acquisition for the larger SLAM study was completed using the method described in this manuscript within 3 months, and with similar proteomic depth (>4000 cumulative proteins and ~2400 proteins per each 20µl serum sample), so we are confident in the scalability and applicability of the method described in this paper in a larger longitudinal mouse study.

To boost the significance of the current study, we would be happy to provide an additional figure summarizing the surface-level results of the larger SLAM study on request, including protein counts, list of protein/peptide identifications, and instrument time. These results would further demonstrate the scalability of this method and applicability in a longitudinal study for the current manuscript. However, given that the larger proteomic dataset is committed to an ongoing study, the underlying quantitative data from each sample would not be shareable at this time.

Some minor comments:

1) Abstract should clarify that cytokines were primarily detected by PEA, not MS.

Response: Thank you and clarified – page-2, line-40:
"With 1 µL additional serum, a 48-plex Olink Proximity Extension Assay (PEA) quantified 39 cytokines not detected by MS."

2) Authors should provide at least general information about nanoparticle properties (like charge, size) instead of only calling them 'proprietary'.

Response: We appreciate the reviewer's suggestion for additional information regarding NPs properties. While the exact formulations, sizes, and surface chemistries of the NPs used in the

Proteograph workflow are proprietary. Although we agree that providing general physicochemical context might increase transparency and interpretation.

Proteograph workflow employs a panel of engineered nanoparticles with diverse physicochemical properties, including variation in surface charge, hydrophobicity, and functional chemistry, as described previously (PMID: 32699280). These NPs are based on superparamagnetic iron oxide nanoparticles (SPION) cores enabling automated magnetic capture and washing steps. Although specific dimensions are not disclosed, the particles fall within the typical size range (SEM data showed three SPIONs core are 200–300 nm with spherical and semi-spherical morphologies) used for synthetic protein capturing nanomaterials. Notable, the heterogeneity in surface characteristics is designed to promote formation of distinct, and reproducible protein coronas, allowing selective enrichment of proteins spanning a wide dynamic range. This corona formation effectively reduces sample complexity, enhances detection of low-abundance proteins, and supports deep proteome coverage in downstream MS analysis.

We have added a brief description of the NP to the Methods section, to provide greater transparency while acknowledging proprietary constraints, page-16, line-491:

"The workflow uses a subset of engineered NPs with diverse surface chemistries, including variations in charge and functional groups, built on superparamagnetic iron oxide nanoparticle (SPION) cores to enable magnetic capture, as previously described (15). Although the exact characteristics of the commercially available NP kits are proprietary, they are selected from among the library of NPs characterized previously characterized in more detail (15). Each NP forms distinct and reproducible protein coronas that, when used alone or in combination, enhance proteome depth."

Recommendation: Major revision While this manuscript presents an interesting application of nanoparticle-assisted proteomics to low-volume mouse serum, it suffers from critical design, statistical, and methodological weaknesses that, in my point of view, preclude publication in journals that requires robust data presentation and novelty such as Nature Aging.

Reviewer #3 :

This study introduces and validates a nanoparticle-based, data-independent acquisition (DIA) mass-spectrometry workflow that enables deep and quantitative proteomic profiling of low-volume mouse serum across the lifespan, complemented by a proximity extension assay for cytokines

(Olink). The authors identified around 4,000 proteins including >200 senescence-associated markers, and, using 1 µL of additional serum, quantified 39 further cytokines typically missed by MS. The quantitative accuracy was high (median CV < 10%) and strong agreement between NP fractions was reported. The authors applied the pipeline to 30 mice aged 12, 24 and 30 months and revealed age- and sex-dependent proteomic signatures and correlations with glucose and body fat. The paper demonstrates a good level of benchmarking, and the complementary use of Olink to capture low-abundance cytokines was appropriate. The manuscript is well-written, and the methodology is sound.

Although the paper reports interesting outcomes, it has quite important weaknesses.

1) One of the major issues is the small, cross-sectional cohort (limiting statistical power and causal inference), the absence of independent validation or direct human translation. One interesting aspect of the paper though, is the spike in experiment, where the author shows that nanoparticle corona preserves the quantitative differences and fold changes and is truly representing the serum quantities of proteins.

Response: We agree with the reviewer's comments about the study design and appreciate acknowledgement of the quantitative benchmarking component of the study – this was indeed a critical experiment for building our own confidence in this workflow. We agree that the small, cross-sectional cohort limits statistical power, precludes causal inference, and does not allow for direct validation or immediate human translation. Below we address each limitation in more detail, including 1) human translation, 2) independent validation, and 3) sample size and study design. We have included additional discussion, experiments and results to address some of these limitations, including new results/figures exploring human relevance and independent validation of biological changes with existing literature. With regard to #3, we also discuss ongoing work on a much larger study for more definitive biological and longitudinal insights.

1) **Human translation -** We appreciate the suggestion and agree that it would be of value to evaluate the relevance of these findings in humans. To address the comparison with existing human aging data, the revised manuscript incorporates a cross- species comparison between the mouse proteomic signatures identified in this study and human plasma proteomics from Baltimore Longitudinal Study of Aging (BLSA) and published UK Biobank proteomics that support the translational relevance of the mouse data.

The newly generated analysis has been included in the results as a main figure (**Fig. 6**). Our analysis reveals that 50% of the age-associated proteins identified in this mouse study are also significantly age-associated in the BLSA. Interestingly, these results suggest a common circulating aging signature of extracellular matrix, endoplasmic reticulum, and lysosomal protein components in humans and mice. We assessed the association of these proteins with frailty, a key clinical aging endpoint, in BLSA and found that five significantly associated with frailty: COL6A3, LBP, PIGR, IGFBP1 and HPX. By far, the ECM protein COL6A3 is the most significant among these (adj. p-value = 5.07e-16). These findings are consistent with recent evidence of age-associated increase in ECM components in Nature Aging (PMID: 41023316), and a broad range of literature associating COL6A3 (aka Endotrophin, the uncleaved precursor) with mortality and chronic diseases (PMID 38871093). Notably, COL6A3 is among the top 5 proteins associated with pre-frailty, and frailty, in the UK Biobank (PMID 39535692), independently validating the association with human frailty observed in BLSA. Overall, these results support clinical relevance of the findings of our study and demonstrate the mouse study can shed light on human aging. We have included the new analysis into the results, figures, and discussion, accordingly, as shown below.

New Figure, **Fig. 6**:

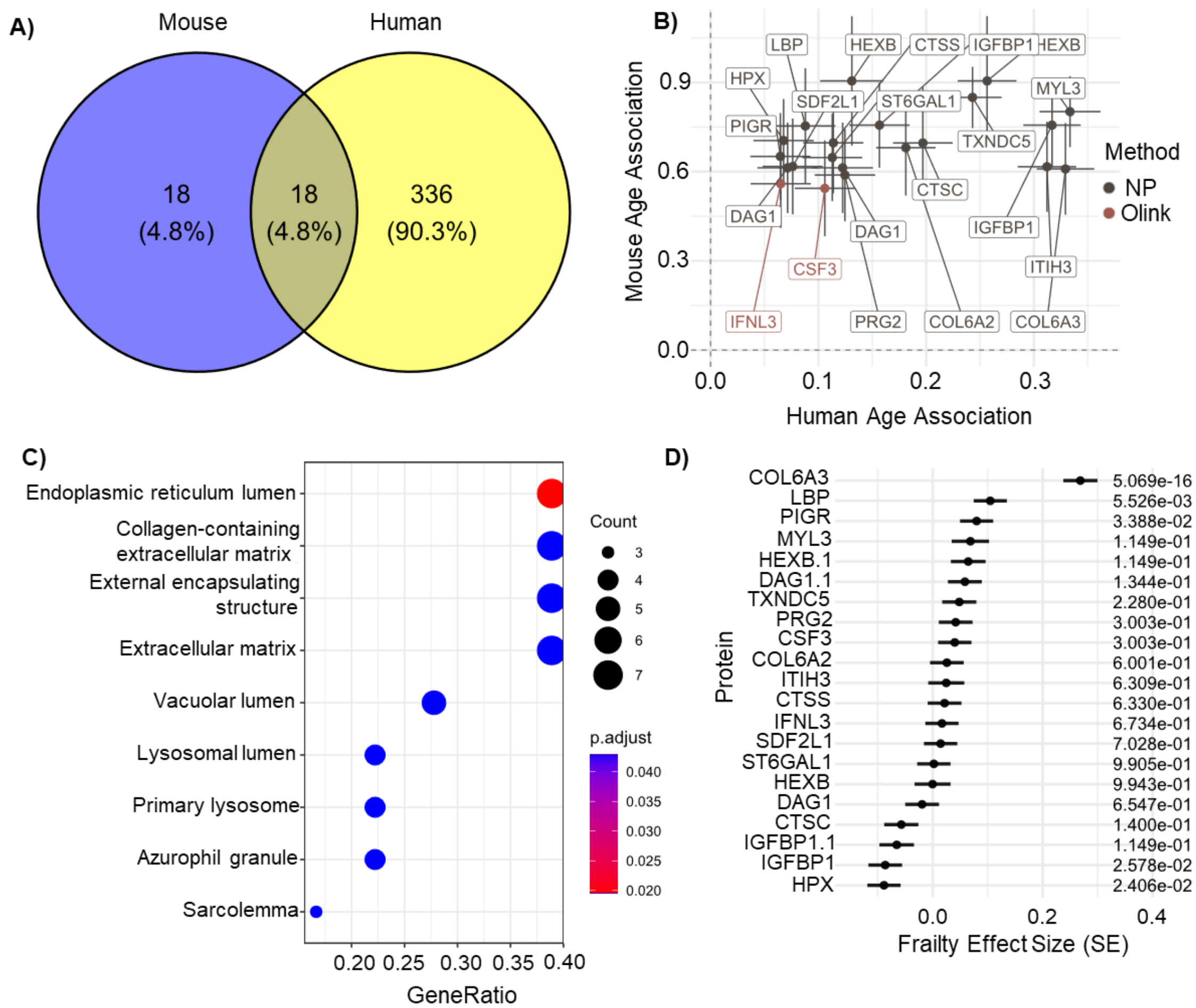

"Figure 6: Circulating proteins associated with age and frailty in mice and humans A) Venn diagram illustrating proteins identified as positively age-associated using Pearson linear modeling across NP-MS workflow and the O-link platforms in the cohort analyzed in this study (left blue), and proteomic measurements (SomaScan 7k assay) in the BLSA (right yellow), modeled as: age ~ protein + sex (Total proteins quantified across both datasets, N = 1742). B) Proteins significantly associated with age in mice and humans, determined using Pearson linear modeling (age ~ protein + sex). Color coding indicates detection method in this study. C) Overrepresentation analysis of proteins significantly upregulated with age in both species, annotated by cellular component. D) Associations between circulating proteins and frailty assessed using Pearson linear modeling across the cohort analyzed in this study and the BLSA, based on a 44-component frailty index and the model: frailty index ~ protein + age + sex. "

2) **Independent validation –** To address the need for independent validation of the aging changes measured in protein coronas we performed additional analysis and comparisons with existing literature. Targeted cytokine measurements with the Proximity Extension

Assay, and antibody-affinity-based platform, were a major component of this study. To perform a cross-platform comparison of a protein measured by both methods, nanoparticle-based MS versus antibody-based, we searched for overlapping protein observations between datasets and found a single protein – IL16 – with enough observations across mice to perform a comparison (Fig. 3C-D, 11 mice with matched measurements in both platforms). Using linear modeling, we found significant correlation between platforms in the measurement of IL16 ($p=0.0026$, $R = 0.81$). This example is a minor supportive factor in our claim of accurate representation of protein levels in the protein corona when compared with the original serum samples. To further validate whether our observed changes align with expected biology and proteomic measurements from prior studies, we performed an analysis comparing our measured changes to two published MS-based proteomic datasets in mouse blood that did not use nanoparticles. Although there are no prior mouse aging studies in blood to draw from, the two published studies examined sex-associated changes in mouse serum (PMIDs 37910662 and 34050187). Notably, with the exception of two proteins (Transcobalamin-2 and Hepatocyte growth factor activator) out of thirty-two, all sex-associated proteins from our study that were also reported in either published study were directionally aligned and correlated. This has been added as a supporting figure, **Fig. S3**, and is numerically detailed in Table S4. Overall, these findings further support that protein corona measurements in our study align with direct measurements in blood samples and accurately reproduce expected biological changes, such as sex-dimorphism.

New figure, **Fig. S3**:

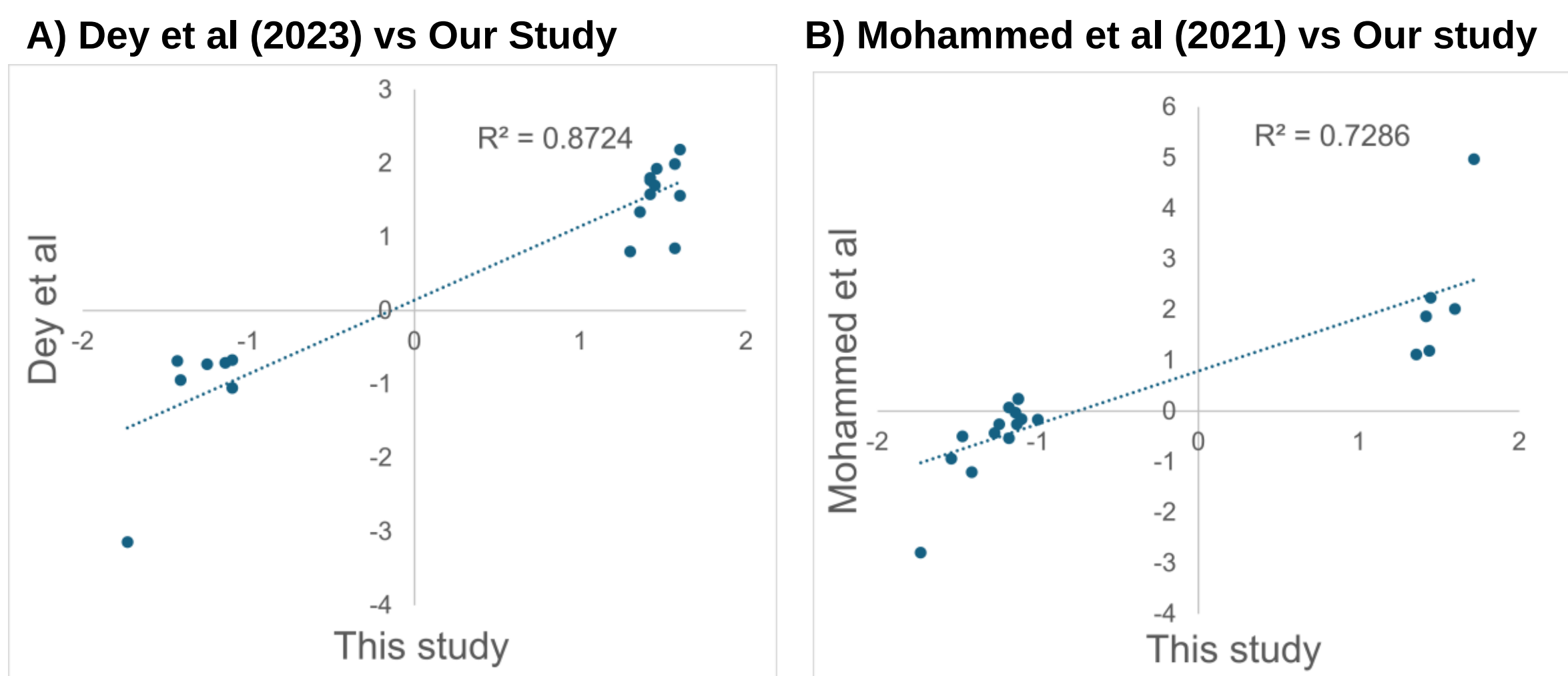

"**Supplementary Fig. 3- Correlation of sex-associated circulating proteins between this study and published datasets.** Correlation plots depicting sex-associated changes in our study versus those reported in A) Dey et. al. (PMID: 37910662); and B) Mohammed et. al. (PMID- 34050187). Proteins in both studies show strong positive correlations with this study (R^2=0.8724 and R^2=0.7286, respectively), and consistent directionality of sex associated effects."

Supplemental Table 4: Validation of top sex associated proteins with other study

Sl. No.	Gene	Uniprot ID	Description	SLAM B-sex	Dey et. al. JPR,2023 log2(M/F)	Mohammed et. al. NPJ Syst. Biol. Appl. 2021 log2(M/F)
1	Serpina1e*	Q00898	Alpha-1-antitrypsin	**1.72**		**4.97**
2	C8g*	Q8VCG4	Complement component C8 gamma chain	**1.6**	**1.56**	**2.02**
3	C6	E9Q6D8	Complement component 6	**1.6**	**2.19**	
4	C7	D3YXF5	Complement component 7	**1.57**	**1.99**	
5	C8b*	Q8BH35	Complement component C8 beta chain	**1.45**	**1.7**	**2.24**
6	C8a*	Q8K182	Complement component C8 alpha chain	**1.42**	**1.77**	**1.87**
7	Ces3a	Q63880	Carboxylesterase 3A	**1.57**	**0.85**	
8	Mug1*	P28665	Murinoglobulin-1	**1.44**		**1.19**
9	Mup20	Q5FW60	Major urinary protein 20	**1.46**	**1.93**	
10	Crisp1	Q03401	Cysteine-rich secretory protein 1	**1.42**	**1.8**	
11	Mup3	P04939	Major urinary protein 3	**1.42**	**1.58**	
12	Egfr*	Q01279	Epidermal growth factor receptor	**1.36**	**1.34**	**1.12**

13	Serpina3k	P07759	Serine protease inhibitor A3K	1.3	0.8	
14	Tcn2	O88968	Transcobalamin-2	-1.12		0.25
15	Glycam1*	Q02596	Glycosylation-dependent cell adhesion molecule 1	-1.73	-3.14	-2.79
16	Adipoq*	Q60994	Adiponectin	-1.54		-0.94
17	Bche	Q03311	Cholinesterase	-1.47		-0.5
18	Serpina6*	Q06770	Corticosteroid-binding globulin	-1.41	-0.94	-1.2
19	Scg3	P47867	Secretogranin-3	-1.43	-0.68	
20	Ctsc	P97821	Dipeptidyl peptidase 1	-1.1	-1.05	
21	Afm	O89020	Afamin	-1.18		-0.53
22	Gsn	P13020	Gelsolin	-1.1		-0.16
23	Hgfac	Q9R098	Hepatocyte growth factor activator	-1.18		0.07
24	Igfals	P70389	Insulin-like growth factor-binding protein complex acid labile subunit	-1.27		-0.43
25	Itih1	Q61702	Inter-alpha-trypsin inhibitor heavy chain H1	-1.14		-0.03
26	Itih2	Q61703	Inter-alpha-trypsin inhibitor heavy chain H2	-1.13		-0.26
27	Itih4	A6X935	Inter alpha-trypsin inhibitor, heavy chain 4	-1		-0.17
28	Lum	P51885	Lumican	-1.24		-0.26
29	Psmb4	P99026	Proteasome subunit beta type-4	-1.12		0.24
30	Pi16	Q9ET66	Peptidase inhibitor 16	-1.1	-0.67	
31	Retn	Q99P87	Resistin	-1.25	-0.73	
32	Igfbp5	Q07079	Insulin-like growth factor-binding protein 5	-1.14	-0.71	

Red- Higher in males; **Blue**- Higher in Females; *-97% (C-statistics) discrimination between males and females (Mohammed et. al. 2021)

3) Sample size and study design - As the reviewer recognized, this study was not powered for identifying robust phenotypic changes and biomarker associations in SLAM. Instead, the study was designed to demonstrate a proteomic workflow that enables unprecedented proteomic blood biomarker studies in mice in strictly volume limited conditions and showcase its application in the context of aging. The key strength/contribution of the study is that it introduces a method for comprehensive serum proteomic analysis in mouse cohorts, which are notably missing from existing literature due to the technical challenges associated with such studies. This workflow opens the door to comprehensive serum proteomic studies in mice even in volumes in low as 10 µl (11 µl for the addition of cytokines), a volume requirement that is compatible with longitudinal collection of blood in mice. This volume can also realistically be used for proteomics without consuming an entire serum volume and is thus compatible with studies that utilize blood for multiple different measurements (e.g. 'clinical' labs, proteomics, metabolomics, etc.). Although the current study is not powered to detect phenotypic changes, it is sufficiently powered to identify a larger number of age- and sex-associated proteins in serum than previously described (adjusted p-value < 0.05), reveal interesting aging biology, and potential clinical relevance, as evidenced by our addition of an additional analysis of a subset of protein changes that are conserved with humans (Fig. 6). In order to emphasize the strengths of the study in this framing, we have made the following adjustments:

page-14, line-424:

"Nonetheless, the NP-MS workflow demonstrated strong reproducibility and sensitivity, enabling robust detection of age- and sex--associated signatures. Importantly, this workflow was successful within strict sample volume requirements down to 10uL. Therefore, this method is compatible with the volume limitations of longitudinal mouse studies and can be easily adapted and scaled into large cohort studies with a longitudinal study design. Future phases of the SLAM, incorporating larger sample sizes and true longitudinal sampling will be essential to validate these associations, improve statistical power and generalizability, and more accurately capture temporal relationships and potential causal effects across the lifespan."

We also completely agree that a larger study powered by the identification of longitudinal changes and phenotype-associated biomarker signatures will be of ultimate interest. In fact, a separate, larger study powered for identification of phenotype-associated biomarker signatures is ongoing within our laboratories, and will ultimately demonstrate more detailed biomarker signatures, proteomic clocks, and multi-omic associations in a larger subset (n=900) of SLAM, with up to 6 longitudinal timepoints per animal. Because this is an ongoing, multi-team study that integrates multiple teams and datasets, it is outside the scope of the current manuscript. However, we are happy to share that the proteomic data acquisition for the larger SLAM study was completed using the method described in this manuscript within 3 months, and with similar proteomic depth (>4000 cumulative proteins and ~2400 proteins per each 20ul serum sample), so we are confident in the scalability and applicability of the method described in this paper in a larger longitudinal mouse study.

To boost the significance of the current study, we would be happy to provide an additional figure summarizing the surface-level results of the larger SLAM study on request, including protein counts, list of protein/peptide identifications, and instrument time. These results would further demonstrate the scalability of this method and applicability in a longitudinal study for the current manuscript. However, given that the larger proteomic dataset is committed to an ongoing study, the underlying quantitative data from each sample would not be shareable at this time.

To focus our manuscript on the key contributions of the study and temper the conclusions from marginally significant findings, we present our biological findings as hypothesis-generating rather than definitive and emphasize the need for confirmation in larger and longitudinally sampled cohorts, page- 11, line- 323:

“However, because the proteomic data derives from a cross-sectional subset of the longitudinal SLAM cohort, age-associated signatures reflect population-level differences rather than within- individual aging trajectories. All biological interpretations are therefore framed conservatively; and the results should be viewed as foundational and hypothesis-generating rather than mechanistic or causal.”.

Page-14, line-445:

"Overall, while these findings are cross-sectional, they establish a robust methodological framework to enable longitudinal and mechanistic investigations of aging and biomarker discovery in sample-limited mouse studies, and demonstrate translation relevance to human health."

Overall, we hope these results provide a strong methodological foundation for future studies in larger mouse cohorts and for eventual translation to human biomarker research, where quantitative accuracy is essential.

2) Finally, the critical roles of metabolite and lipid variations and their effects on protein corona have not been considered (several reports revealed that variations in metabolites and lipid profiles of plasma proteomics can substantially change the protein corona profile at the surface of nanoparticles); therefore, it is not clear that the observed variations originate from plasma proteome variations or reflection of metabolomics and lipidomic profile variation in plasma that caused changes in the composition of protein corona. This could be very important, as the authors were interested in proteomics signatures associated with glucose and body fat.

Response: This is an important point, we appreciate the importance of accounting for potential matrix effects, particularly metabolite and lipid profiles, on the protein corona. We hope the combination of responses presented below sufficiently address the underlying concerns of the reviewer.

First, we contend that the 'gold standard' method to demonstrate whether changes in the protein corona are representative of 'real' changes in the original serum samples at the proteome level (across a wide range of proteins) is to perform quantitative benchmarking (PMID: 27701404) on the same nanoparticles utilized in our study. This concern motivated us to conduct our own independent benchmarking study, shown in **Fig. 2A-B** (see below). Given that human and bovine blood are distinct in their compositions (including metabolites and lipids), if lipids/metabolites had a significant confounding effect on the composition of the protein corona, our expectation would be that our observed protein ratios would be skewed and inaccurate. However, our data suggests that on average the quantitative ratios of proteins in the protein corona are representative of true ratios of the original blood samples across a range of dilutions (5% - 100%).

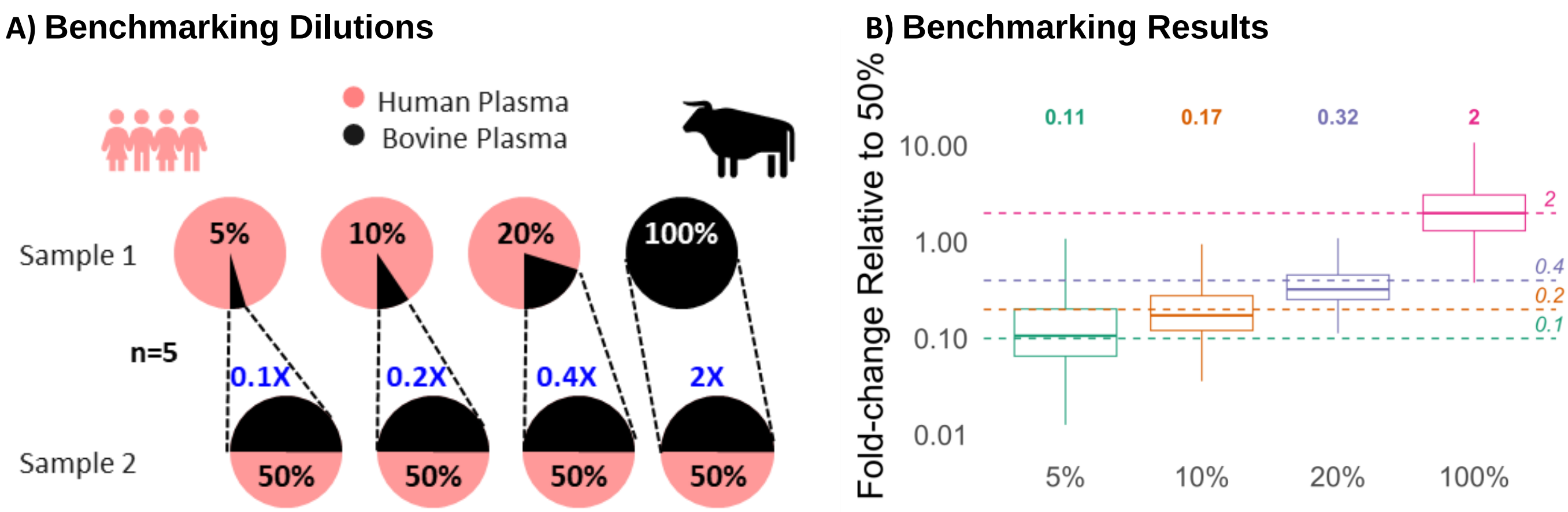

Figure 2: Qualitative and quantitative evaluation of proteomic data generated NP-coupled MS workflow. A) A quantitative benchmarking study was performed using five fixed ratios of human and bovine plasma (n=5 each). B) Boxplot showing the measured peptide ratios in each dilution versus the 50% bovine plasma sample. Dotted lines depict the expected ratios.

Second, we emphasize that published literature, which has more rigorously and comprehensively examined characteristics of the protein corona and binding properties of the nanoparticles utilized in this study, suggests that the effect of lipids and matrix do not significantly impact quantitation. We note that the nanoparticles used in this study are distinct from the plain polystyrene nanoparticles in the study cited by Reviewer #1 (PMID: 39511193) and may not exhibit the same binding behavior. The surface-functionalized superparamagnetic iron nanoparticles in this study are characterized in more detail elsewhere (PMID: 35275789). Lipid spike-in experiments have been done on the same nanoparticles we used, showing that spiking varying amounts of lipid do not significantly affect the number/intensity of proteins (PMID: 32699280, Nature Comms - last paragraph of p5 & first on p6, Fig. S4 and S5). Another paper in JPR (PMID: 41105214) reported on the minimal impact of background matrix (presumably with different lipid/metabolite compositions) on quantitative accuracy. We have noted these additional studies in the discussion (quoted below).

We explained now in the revised “Discussion” section for more clarity, page-10 , line-295:

“Concerns have been raised that NP- coronas may be sensitive to non-protein serum components, such as lips or metabolites, potentially introducing variability unrelated to biological differences. While recent work has shown that small molecules can influence protein corona

composition derived from plain polystyrene nanoparticles (40), multiple independent studies of the surface-functionalized superparamagnetic iron nanoparticles employed in this study suggest that these effects are not significant. Lipid-spike-in experiments using the same nanoparticles showed no appreciable changes in corona protein identities or intensities (15). Similarly, a prior report suggests minimal impact of background matrix on quantitative accuracy (41). Our own quantitative benchmarking data further supports this conclusion, showing high reproducibility and accurate quantification of known fold-changes in samples generated from mixing human and bovine blood, each containing a distinct composition of lipids, metabolites, and other components, in a range of fixed dilutions spanning 5% to 100% (Fig. 2B). Together, these findings indicate that matrix-derived components did not significantly alter corona composition in a manner that distorts relative protein quantification in this study and mitigate concerns matrix effects introduced variability unrelated to aging."

Third, we refer to our earlier response to your first comment on independent validation. Since our findings reproduce known biology, as demonstrated by the strong alignment with previously published proteomic studies, our confidence in the measured proteomic abundances in the protein corona is further strengthened (detailed in part 2 of the response to your first comment above).

Together, these data support that the nanoparticle system used in this study is relatively robust to lipid/metabolite variability, and that the proteomic differences we report most likely reflect underlying biological variation rather than matrix-driven artifacts.

3) I think in page 6, Supplementary Figure 2A is wrongly cited, as it does not show the CV.

Response: We appreciate the reviewer's careful reading of the manuscript. Supplementary Figure 2A is indeed the correct citation, although it shows CV percentage rather than CV. **Supplementary Figure 2A** presents the coefficient of variation (CV) distribution across technical triplicates for NP2, which is the dataset referenced in the corresponding sentence. We have reviewed the figure and text to ensure clarity and confirm that the citation accurately reflects where the CV data is displayed (8.649 or about 9.0%).

4) Overall, the figure quality is not satisfactory, for example, too many labels, font size consistency and axis lines should be corrected/added.

Response: We appreciate this comment and have adjusted figures accordingly to reduce the excessive text labels and make font sizes more consistent throughout.

REPONSE TO REVIEWER COMMENTS

NATAGING-A10220: Deep and Quantitative Proteomic Profiling of Low Volume Mouse Serum: A Cross Sectional of Aging

We are grateful to the editor and all the reviewers for the thorough review of the manuscript and opportunity to appeal the publication decision. We have thoroughly responded to and incorporated the suggested changes to the best of our ability in the revised manuscript. The revision includes the addition of the requested new analysis exploring the translational potential of our results in humans by comparing with a large-scale proteomic dataset, and associated clinical parameters, from the Baltimore Longitudinal Study of Aging (BLSA). We believe the manuscript has substantially improved in quality, focus, and rigor as a result of the feedback from reviewers.

Below are point-by-point responses to reviewer comments:

Reviewers' Comments:

REVIEWER #1:

1. My main suggestion is related to comparing the data generated in this study with some large-scale human plasma proteomics data published recently using the UK Biobank. The comparison will be necessary to build the confidence that the mouse study will shed valuable light on human aging, which is critical for this study.

Response: We appreciate the suggestion and agree that comparison with large-scale human plasma proteomics datasets is important to establish confidence that mouse serum proteomics can inform human aging biology. To address this point, the revised manuscript incorporates a cross- species comparison between the mouse proteomic signatures identified in this study and human plasma proteomics from Baltimore Longitudinal Study of Aging (BLSA) and published UK Biobank proteomics that support the translational relevance of the mouse data. While our access to the UK Biobank data is forthcoming, given our initial findings, we are quite enthusiastic about further expanding the analysis into the expansive proteomic dataset.

The newly generated analysis has been included in the results as a main figure (**Fig. 6**). Our analysis reveals that 50% of the age-associated proteins identified in this mouse study are also significantly age-associated in the BLSA. Interestingly, these results suggest a common

circulating aging signature of extracellular matrix, endoplasmic reticulum, and lysosomal protein components in humans and mice. We assessed the association of these proteins with frailty, a key clinical aging endpoint, in BLSA and found that five significantly associated with frailty: COL6A3, LBP, PIGR, IGFBP1 and HPX. By far, the ECM protein COL6A3 is the most significant among these (adj. p-value = 5.07e-16). These findings are consistent with recent evidence of age-associated increase in ECM components in Nature Aging (PMID: 41023316), and a broad range of literature associating COL6A3 (aka Endotrophin, the uncleaved precursor) with mortality and chronic diseases (PMID 38871093). Notably, COL6A3 is among the top 5 proteins associated with pre-frailty, and frailty, in the UK Biobank (PMID 39535692), independently validating the association with human frailty observed in BLSA. Overall, these results support clinical relevance of the findings of our study and demonstrate the mouse study can shed light on human aging. We have included the new analysis into the results, figures, and discussion, accordingly, as shown below.

New Figure, Fig. 6:

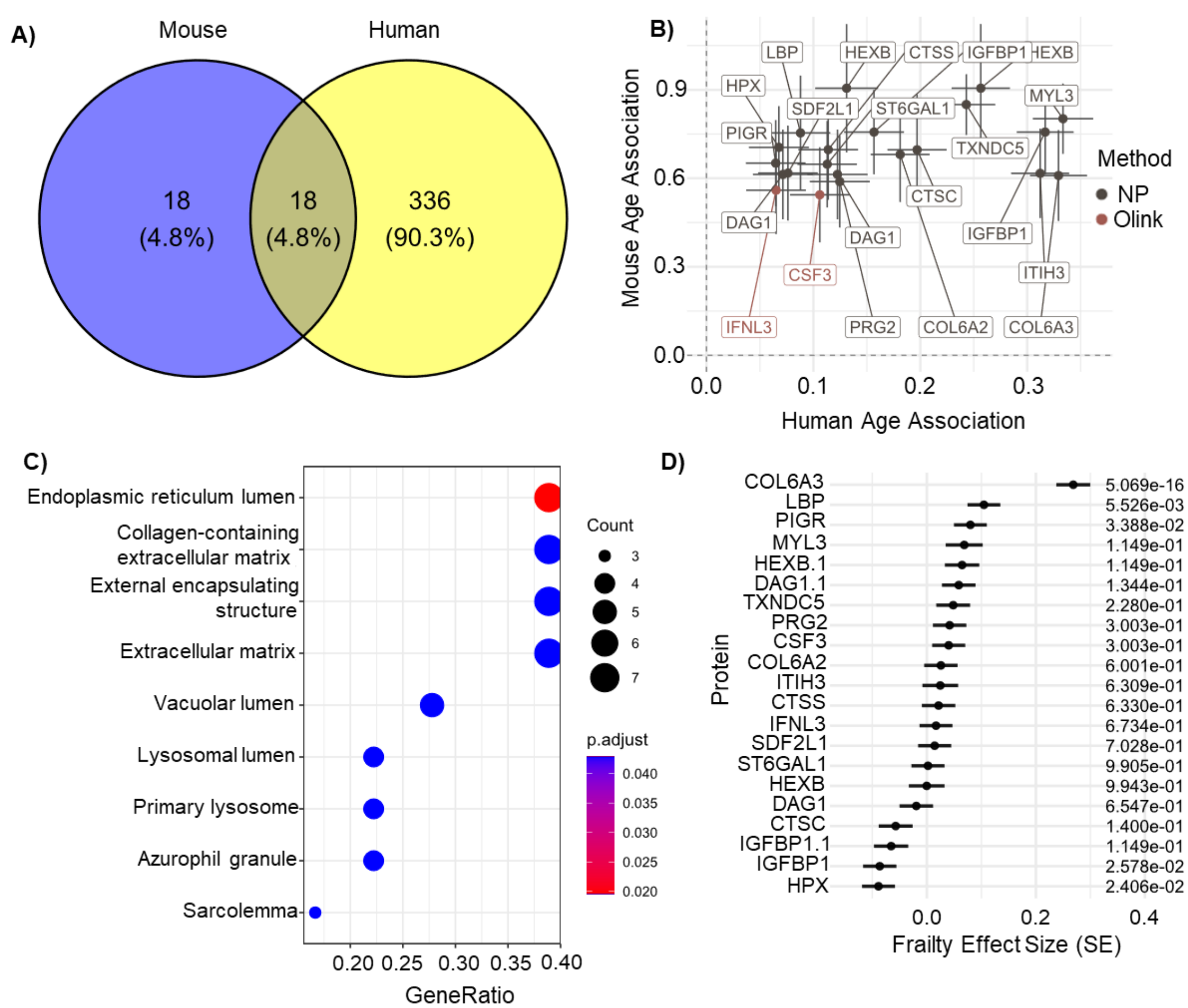

“Figure 6: Circulating proteins associated with age and frailty in mice and humans A) Venn diagram illustrating proteins identified as positively age-associated using Pearson linear modeling across NP-MS workflow and the O-link platforms in the cohort analyzed in this study (left blue), and proteomic measurements (SomaScan 7k assay) in the BLSA (right yellow), modeled as: age ~ protein + sex (Total proteins quantified across both datasets, N = 1742). B) Proteins significantly associated with age in mice and humans, determined using Pearson linear modeling (age ~ protein + sex). Color coding indicates detection method in this study. C) Overrepresentation analysis of proteins significantly upregulated with age in both species, annotated by cellular component. D) Associations between circulating proteins and frailty assessed using Pearson linear modeling across the cohort analyzed in this study and the BLSA, based on a 44-component frailty index and the model: frailty index ~ protein + age + sex. “

New analysis described in the Results section, page-9, line-253:

“To assess cross-species concordance, we compare age-associated circulating proteins in mice with human plasma proteomics from the BLSA (35, 36). Among 1742 proteins quantified in both datasets, Pearson Linear modeling (age ~ protein + sex) identified 18 proteins, representing 5% of the total proteins or 50% of mouse age-associated proteins, to be significantly (**Fig. 6A**) and positively (**Fig. 6B**) associate with age in both mice and humans. Overrepresentation analysis of shared upregulated proteins with age in both mice and humans revealed enrichment for cellular component categories (**Fig. 6C**) including endoplasmic reticulum, extracellular matrix, and lysosomal proteins, among others, and suggesting shared categories of age- associated circulating factors at a pathway-level. To assess the relevance of these findings with age-associated clinical phenotypes, we evaluated the associations of these proteins with frailty index, a 44-component composite score, in the BLSA. Notably, five proteins including COL6A3, LBP, PIGR, and IGFBP1, and HPX were significantly associated with frailty index in the BLSA (Frailty Index ~ Protein + age + sex; **Fig. 6D**). Collectively, these results link conserved proteomic aging signatures to functional health decline in humans and suggest that aging-associated biomarkers derived from mice may be relevant in humans.”

We have emphasized these cross-species findings in the Abstract, page-2:

“Cross-species comparison with human plasma proteomics from the Baltimore Longitudinal Study of Aging (BLSA) identified conserved age-associated circulating proteins, several of which were linked to frailty in humans.”

Finally, we elaborated on the significance of these results in the Discussion, page-13, line- 394:

“To place the mouse findings in a translational context, we compared age-associated circulating proteins identified here with human plasma proteomics from BLSA. Despite differences in species, cohort design, and analytical platforms, a subset of proteins, representing half of the mouse age-associated proteins, were concordantly age- associated in humans (**Fig. 6A-B**), indicating conserved features of the circulating aging proteome. Interestingly, collagen-containing extracellular matrix proteins were overrepresented among these, consistent with recent reports of the association of extracellular matrix components, including collagen, with aging in mouse circulation (66). Moreover, several of these shared proteins, including COL6A3, LBP, PIGR, IGFBP1, and HPX, were also significantly associated with frailty in humans, linking shared circulating proteomic signatures to functional health decline. Notable among these, Collagen Type VI Alpha 3 Chain (COL6A3, a.k.a. Endotrophin), a well-known indicator of fibrotic aging, was by far the most significant frailty-associated protein in the BLSA (adjusted p-value = 5.069e-16). COL6A3 has been associated with mortality and a range of non-communicable chronic diseases linked to aging, including idiopathic pulmonary fibrosis, chronic kidney disease, type 2 diabetes, cardiac dysfunction, among others (67). Remarkably, COL6A3 is also among the top five proteins associated with pre-frailty and frailty in the UK Biobank (68), independently validating the robustness of this finding in humans. Although exploratory, these findings collectively support the translational relevance of mouse serum proteomics and suggest that at least a subset of aging-associated circulating proteins is conserved in humans. While we expect the correlation of mouse and human age-associated proteins as a whole to be limited due to the substantial influence of genetics and other factors on the circulating proteome, we speculate that a key subset of circulating proteins with mechanistic links to biology will be conserved and translationally relevant. Notable among these in our study are robust aging- and disease-associated proteins such as COL6A3 and lipopolysaccharide-binding protein (LBP), and sex-associated proteins such as Complement 8 (conserved association with the male sex) and adiponectin (ADIPOQ, conserved association with females).”

2. The NP protein corona composition can be strongly influenced by other molecules in the plasma sample, like metabolites. [PMID: 39511193] Therefore the heterogeneity across plasma/serum samples may influence the protein corona substantially, nothing related to the aging. Therefore, the differentially expressed proteins across the three ages studied here need some additional verification using some high-throughput antibody-based approach. Choosing

several proteins from the differentially expressed pool for additional confirmation will be sufficient.

Response: This is an important point and we appreciate the importance of considering potential confounding effects on the composition of the protein corona, and the critical need to demonstrate the quantitative aging changes derived from the protein corona are representative of differences in the original serum samples. We have thoroughly addressed and acknowledged these concerns with the inclusion of additional analysis and discussion. We hope the combination of responses presented below sufficiently address the underlying concerns of the reviewer.

First, we contend that the 'gold standard' method to demonstrate whether changes in the protein corona are representative of 'real' changes in the original serum samples at the proteome level (across a wide range of proteins) is to perform quantitative benchmarking (PMID: 27701404) on the same nanoparticles utilized in our study. This concern motivated us to conduct our own independent benchmarking study, shown in **Fig. 2A-B** (see below). Given that human and bovine blood are distinct in their compositions (including metabolites and lipids), if lipids/metabolites had a significant confounding effect on the composition of the protein corona, our expectation would be that our observed protein ratios would be skewed and inaccurate. However, our data suggests that on average the quantitative ratios of proteins in the protein corona are representative of true ratios of the original blood samples across a range of dilutions (5% - 100%).

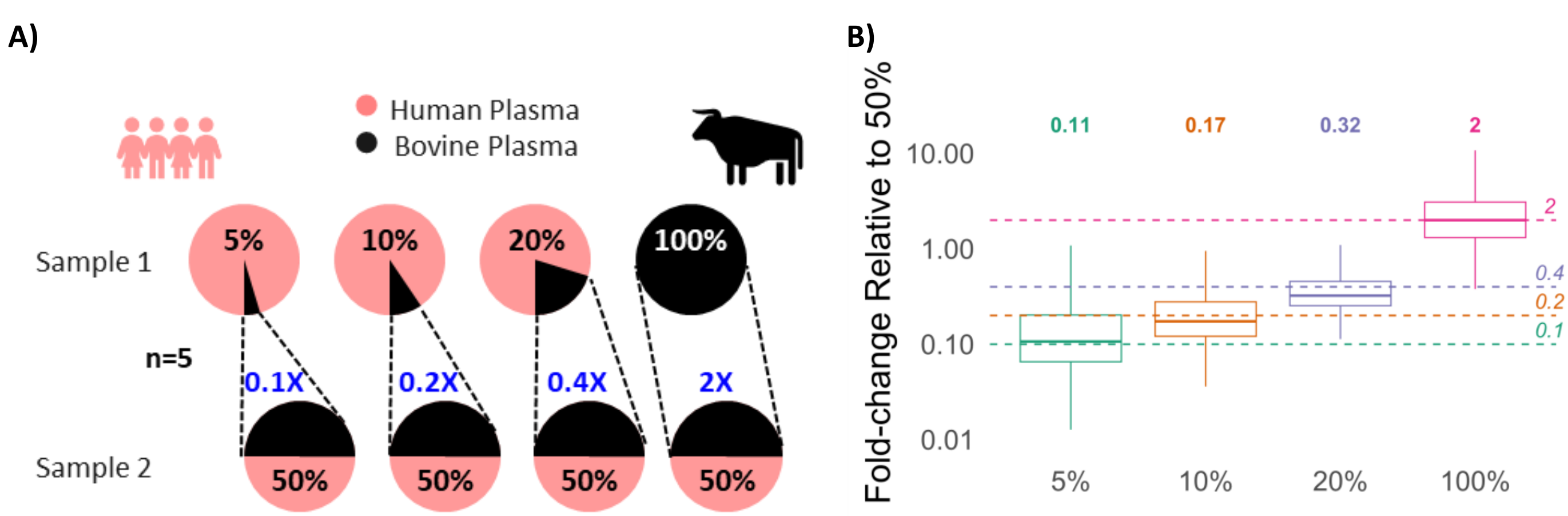

"Figure 2: Qualitative and quantitative evaluation of proteomic data generated NP-coupled MS workflow. A) A quantitative benchmarking study was performed using five fixed ratios of

human and bovine plasma (n=5 each). B) Boxplot showing the measured peptide ratios in each dilution versus the 50% bovine plasma sample. Dotted lines depict the expected ratios.**"**

Second, we emphasize that published literature, which has more rigorously and comprehensively examined characteristics of the protein corona and binding properties of the nanoparticles utilized in this study, suggests that the effect of lipids and matrix do not significantly impact quantitation. We note that the nanoparticles used in this study are distinct from the plain polystyrene nanoparticles in the study cited by the reviewer (PMID: 39511193) and may not exhibit the same binding behavior. The surface-functionalized superparamagnetic iron nanoparticles in this study are characterized in more detail elsewhere (PMID: 35275789). Lipid spike-in experiments have been done on the same nanoparticles we used, showing that spiking varying amounts of lipid do not significantly affect the number/intensity of proteins (PMID: 32699280, Nature Comms - last paragraph of p5 & first on p6, Fig. S4 and S5). Another paper in JPR (PMID: 41105214) reported on the minimal impact of background matrix (presumably with different lipid/metabolite compositions) on quantitative accuracy. We have noted these additional studies in the discussion (quoted below).

We explained now in the revised "Discussion" section for more clarity, page-10, line- 295:

"Concerns have been raised that NP- coronas may be sensitive to non-protein serum components, such as lips or metabolites, potentially introducing variability unrelated to biological differences. While recent work has shown that small molecules can influence protein corona composition derived from plain polystyrene nanoparticles (40), multiple independent studies of the surface-functionalized superparamagnetic iron nanoparticles employed in this study suggest that these effects are not significant. Lipid-spike-in experiments using the same nanoparticles showed no appreciable changes in corona protein identities or intensities (15). Similarly, a prior report suggests minimal impact of background matrix on quantitative accuracy (41). Our own quantitative benchmarking data further supports this conclusion, showing high reproducibility and accurate quantification of known fold-changes in samples generated from mixing human and bovine blood, each containing a distinct composition of lipids, metabolites, and other components, in a range of fixed dilutions spanning 5% to 100% (**Fig. 2B**). Together, these findings indicate that matrix-derived components did not significantly alter corona composition in a manner that distorts relative protein quantification in this study and mitigate concerns matrix effects introduced variability unrelated to aging."

Third, we attempted to address the reviewer's request for independent validation of the aging changes measured in protein coronas with additional analysis and comparison with literature. Targeted cytokine measurements with the Proximity Extension Assay (PEA), an antibody-affinity-based platform, were a major component of this study. To perform a cross-platform comparison of a protein measured by both methods, nanoparticle-based MS versus antibody-based, we searched for overlapping protein observations between datasets and found a single protein – IL16 – with enough observations across mice to perform a comparison (**Fig. 3C-D**, 11 mice with matched measurements in both platforms). Using linear modeling, we found significant correlation between platforms in the measurement of IL16 ($p=0.0026$, $R = 0.81$). This example further supports our claim of accurate representation of protein levels in the protein corona when compared with the original serum samples. To further validate whether our observed changes align with expected biology and proteomic measurements from prior studies, we performed an analysis comparing our measured changes to two published MS-based proteomic datasets in mouse blood that did not use nanoparticles. Although there are no prior mouse aging studies in blood to draw from, the two published studies examined sex-associated changes in mouse serum (PMIDs 37910662 and 34050187). Notably, apart from two proteins (Transcobalamin-2 and Hepatocyte growth factor activator), all sex-associated proteins from our study that were also reported in either published study were directionally aligned and correlated. This has been added as a supporting figure, **Fig. S3**, and is numerically detailed in **Table S4**. Overall, these findings further support that protein corona measurements in our study align with direct measurements in blood samples and accurately reproduce expected biological changes, such as sex-dimorphism.

New figure, **Fig. S3**:

A)

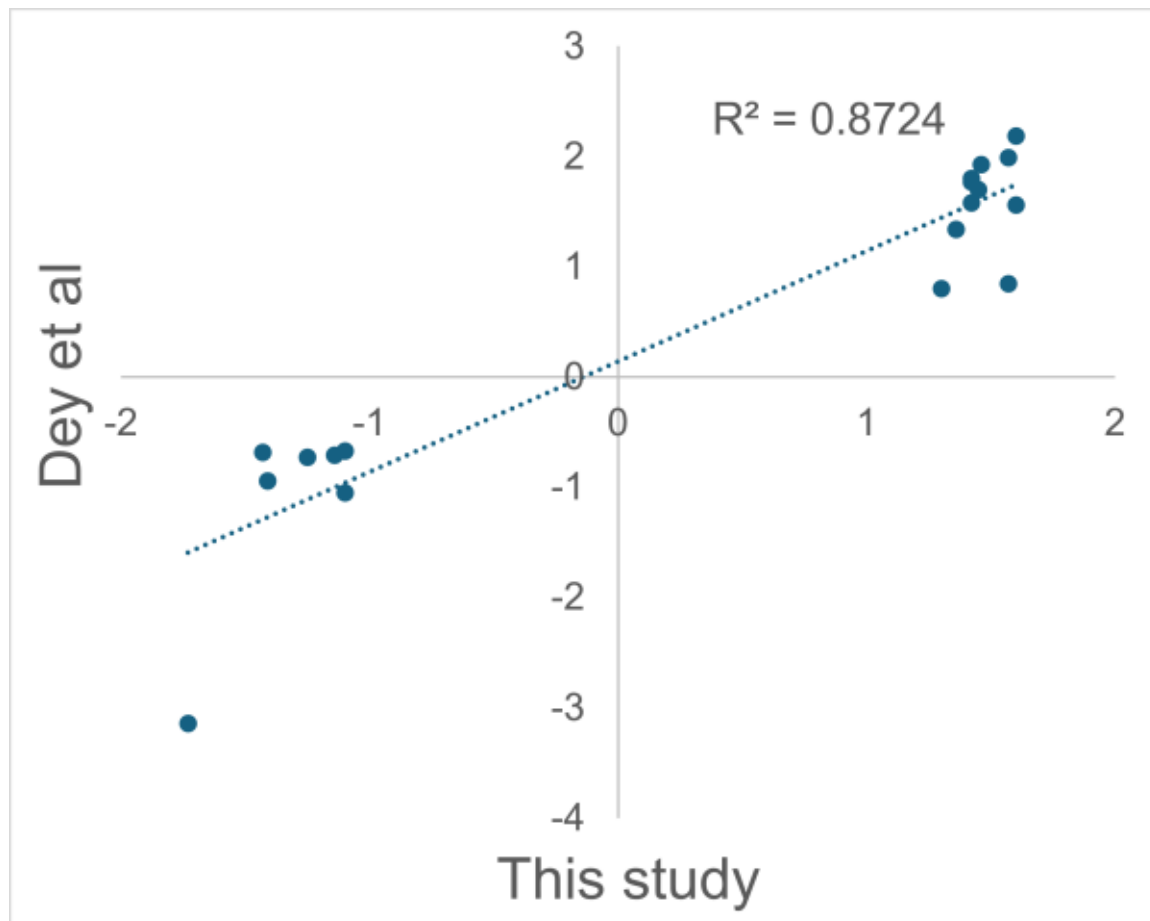

B)

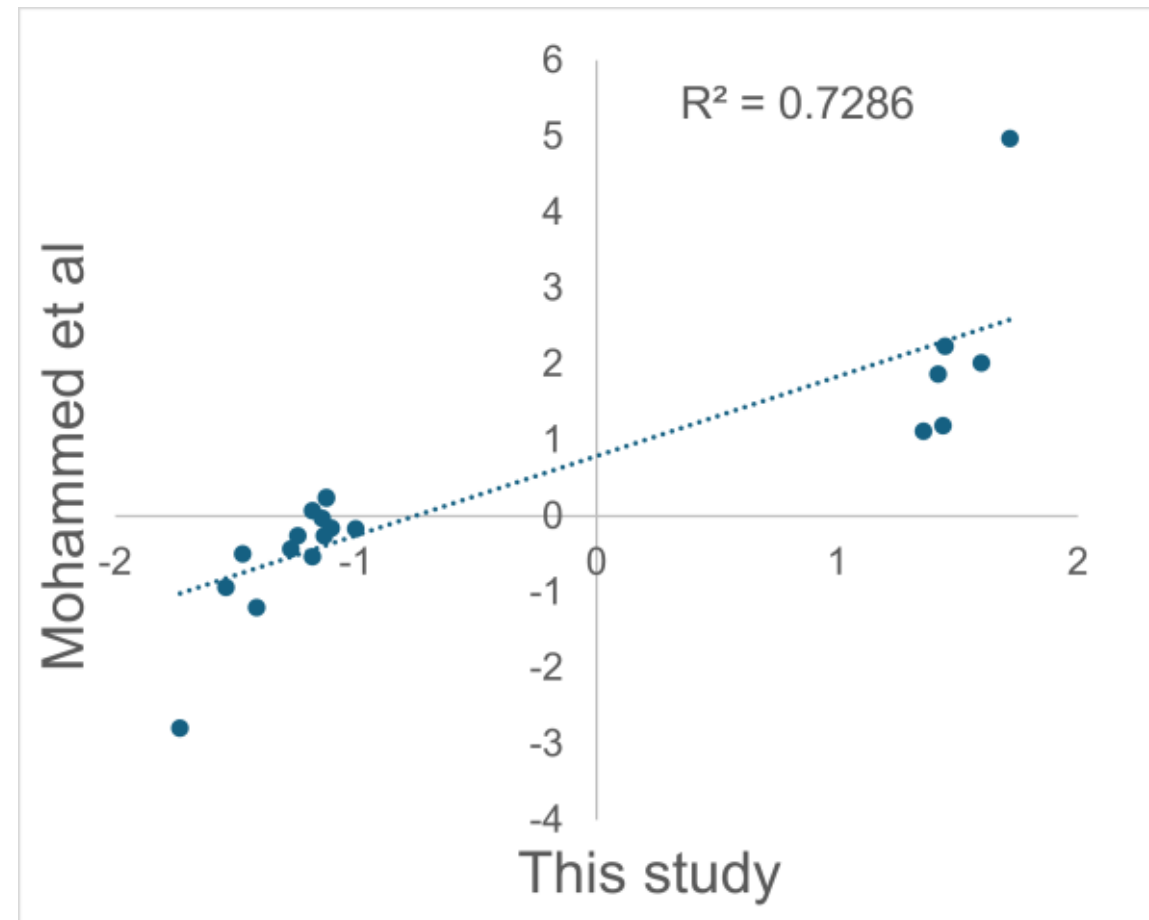

“Supplementary Fig. 3- Correlation of sex-associated circulating proteins between this study and published datasets. Correlation plots depicting sex-associated changes in our study versus those reported in A) Dey et. al. (PMID: 37910662); and B) Mohammed et. al. (PMID-34050187). Proteins in both studies show strong positive correlations with this study (R^2=0.8724 and R^2=0.7286, respectively), and consistent directionality of sex associated effects.”

Supplemental Table 4: Validation of top sex associated proteins with other study

				SLAM	Dey et. al. JPR,2023	Mohammed et. al. NPJ Syst. Biol. Appl. 2021
**Sl. No.**	**Gene**	**Uniprot ID**	**Description**	**B-sex**	**log2(M/F)**	**log2(M/F)**
1	Serpina1e*	Q00898	Alpha-1-antitrypsin	1.72		4.97
2	C8g*	Q8VCG4	Complement component C8 gamma chain	1.6	1.56	2.02
3	C6	E9Q6D8	Complement component 6	1.6	2.19	
4	C7	D3YXF5	Complement component 7	1.57	1.99	

5	C8b*	Q8BH35	Complement component C8 beta chain	1.45	1.7	2.24
6	C8a*	Q8K182	Complement component C8 alpha chain	1.42	1.77	1.87
7	Ces3a	Q63880	Carboxylesterase 3A	1.57	0.85	
8	Mug1*	P28665	Murinoglobulin-1	1.44		1.19
9	Mup20	Q5FW60	Major urinary protein 20	1.46	1.93	
10	Crisp1	Q03401	Cysteine-rich secretory protein 1	1.42	1.8	
11	Mup3	P04939	Major urinary protein 3	1.42	1.58	
12	Egfr*	Q01279	Epidermal growth factor receptor	1.36	1.34	1.12
13	Serpina3k	P07759	Serine protease inhibitor A3K	1.3	0.8	
14	Tcn2	O88968	Transcobalamin-2	-1.12		0.25
15	Glycam1*	Q02596	Glycosylation-dependent cell adhesion molecule 1	-1.73	-3.14	-2.79
16	Adipoq*	Q60994	Adiponectin	-1.54		-0.94
17	Bche	Q03311	Cholinesterase	-1.47		-0.5
18	Serpina6*	Q06770	Corticosteroid-binding globulin	-1.41	-0.94	-1.2
19	Scg3	P47867	Secretogranin-3	-1.43	-0.68	
20	Ctsc	P97821	Dipeptidyl peptidase 1	-1.1	-1.05	
21	Afm	O89020	Afamin	-1.18		-0.53
22	Gsn	P13020	Gelsolin	-1.1		-0.16
23	Hgfac	Q9R098	Hepatocyte growth factor activator	-1.18		0.07
24	Igfals	P70389	Insulin-like growth factor-binding protein	-1.27		-0.43

			complex acid labile subunit			
25	Itih1	Q61702	Inter-alpha-trypsin inhibitor heavy chain H1	**-1.14**		**-0.03**
26	Itih2	Q61703	Inter-alpha-trypsin inhibitor heavy chain H2	**-1.13**		**-0.26**
27	Itih4	A6X935	Inter alpha-trypsin inhibitor, heavy chain 4	**-1**		**-0.17**
28	Lum	P51885	Lumican	**-1.24**		**-0.26**
29	Psmb4	P99026	Proteasome subunit beta type-4	**-1.12**		**0.24**
30	Pi16	Q9ET66	Peptidase inhibitor 16	**-1.1**	**-0.67**	
31	Retn	Q99P87	Resistin	**-1.25**	**-0.73**	
32	Igfbp5	Q07079	Insulin-like growth factor-binding protein 5	**-1.14**	**-0.71**	

Red- Higher in males; **Blue**- Higher in Females; *-97% (C-statistics) discrimination between males and females (Mohammed et. al. 2021)

3. Some new frontiers of NP protein corona study should be cited. for example, top-down proteomics (PMID: 40764671 ; PMID: 39276099; PMID: 39312774) and AI (PMID: 40190726).

Response: Thank you for bringing these studies to our attention. We have now referenced these important recent advances in NP-protein corona research in the “Discussion” with appropriate context, page-14, line- 442:

“Looking ahead, advances such as top-down (69-71) and AI-driven computational modeling (72) hold promise for extending NP-based approaches by resolving intact proteoforms, characterizing post-translational modifications (PTMs), and improving mechanistic interpretation and predictive modeling of circulating biomarkers.”

4. Based on my understanding, this study is not a longitudinal study because they studied different mice at three ages. The authors should clarify this point in the manuscript.

Response: This is correct and we agree that this should be made more explicitly clear that this study is not longitudinal despite utilizing samples from SLAM. Although the SLAM study itself is a longitudinal study, and the work presented here uses a cross-sectional subset of that cohort, different mice were sampled at 12, 24, and 30 months of age, rather than repeatedly sampling the same mice over time. We hope the multiple changes below will eliminate any ambiguity about the study design. That being made clear, we also intend to emphasize that a major benefit of the workflow developed here is that it makes a longitudinal proteomic biomarker study in mouse blood a possibility. By enabling comprehensive, quantitative proteomics in the very limited volumes available in a longitudinal mouse study, such as the 21µL available to us from SLAM, it is compatible with limitations of these study designs. We hope the changes quoted below clearly convey this information.

First, to make the study design clear, we refer to the cross-sectional design in the title of the manuscript, which now reads, "Deep and Quantitative Proteomic Profiling of Low Volume Mouse Serum: A Cross-Sectional Analysis of Aging"

We have made minor adjustments to the introduction to de-emphasize longitudinal studies and emphasize the low-volume compatibility as the underlying benefit for mouse studies of either cross sectional or longitudinal design.

We adjusted the results to introduce the samples as cross sectional: "We applied this method in a cross-sectional subset of 30 C57BL/6J mice from SLAM."

We now explicitly describe in the "Study Design" section that the current analysis as cross-sectional and distinguish it from the broader longitudinal SLAM framework, page-15, line- 463:

"The serum proteomics reported here sample a cross-sectional subset of SLAM, in which different mice were sampled at each age rather than repeatedly sampling the same individuals over time. Accordingly, the age-associated patterns reflect between-group differences, not within-individual longitudinal trajectories."

In addition, in limitations paragraph of “Discussion” we acknowledge that our design is cross-sectional, and that age-related signatures are inferred between distinct groups of mice rather than within individuals, which prevents assessment of true longitudinal trajectories over time. Although, in this study we developed nanoparticle-based enrichment and subsequent LC-MS methodology, which might be suitable for our longitudinal SLAM study in near future, page-14, line-424:

“Nonetheless, the NP-MS workflow demonstrated strong reproducibility and sensitivity, enabling robust detection of age- and sex--associated signatures. Importantly, this workflow was successful within strict sample volume requirements down to 10uL. Therefore, this method is compatible with the volume limitations of longitudinal mouse studies and can be easily adapted and scaled into large cohort studies with a longitudinal study design. Future phases of the SLAM, incorporating larger sample sizes and true longitudinal sampling will be essential to validate these associations, improve statistical power and generalizability, and more accurately capture temporal relationships and potential causal effects across the lifespan.”

5. A proteomics study of low-volume mouse serum samples using nanoparticle protein corona approach was published recently. The authors should cite and mention this paper. [PMID: 40726225]

Response: We thank the reviewer for bringing this recent study [PMID: 40726225] to our attention. We have referenced and mentioned it accordingly.

We incorporated this citation with appropriate context, page- 10, line- 309:
“The recent development of a high-throughput NP-protein corona enrichment in serum and plasma in the context of a mouse cancer model (42) underscores the growing utility of the strategy.”

Reviewer #2 (Remarks to the Author):

1. The reporting and application of false discovery rate (FDR) thresholds are inconsistent across the manuscript. For peptide and protein identification, a 1% FDR threshold is applied, and for age-associated proteins (linear modeling), the authors explicitly report FDR ≤ 0.05. However, for other analyses — including cytokine modeling, pairwise t-tests between age groups, and associations

with glucose and fat percentage — only nominal p-values are reported, with no mention of multiple testing correction. Given the high dimensional nature of proteomic and cytokine datasets, failure to consistently apply FDR inflates the risk of false positives and undermines the robustness of the reported associations. The authors must apply and report uniform FDR correction across all analyses. Without this, the reliability of many of the claimed findings is questionable.

Response: We thank the reviewer for careful evaluation and agree that consistent and transparent application and reporting of statistical cutoffs, including multiple- testing adjustment, is critical for rigor and reliability of the findings. As noted by the reviewer, we applied the standard combination of 1% FDR cutoff for proteomic IDs and FDR < 0.05 for relative quantification of aging changes by MS but were less stringent in the subsequent analysis. We resolved inconsistencies by standardizing the statistics in the main text and figures with a significance threshold of adjusted p-value < 0.05. Both p-values and adjusted p-values are now reported in the supplementary tables. We hope this provides adequate consistency and transparency in the application and presentation of statistics.

Regarding sample size, statistical power, and the robustness and reliability of claimed findings – we agree these are of critical importance and refer the reviewer to reviewer to the next response, which addresses this in more detail.

2. The biological conclusions are drawn from a relatively small cohort (n=30 mice, 10 per age group), which limits statistical power and generalizability. The cross-sectional design (different mice at each age) means that age-related trends are inferred between groups rather than within individuals. As the authors acknowledge, this precludes assessing individual longitudinal trajectories or causal relationships over time. While the data are intriguing, some findings (especially the finer details of non-linear changes or extensive lists of associated proteins) should be interpreted with caution due to the modest sample size

Response: We appreciate the reviewer's thoughtful comment and agree that it is important that the biological interpretations are not over-stated when the study is not powered to support them. To address these concerns, we have 1) performed additional PEA (O-link) proteomic assays for increased statistical power, 2) re-framed our claims to focus on the key (methodological) findings of the study, 3) adjusted language to mitigate longitudinal inference, and 4) substantially tempered or removed claims and figures related to modest trends and nominal statistics.

1) To increase statistical power of the PEA (O-link) proteomic assays, we performed additional experiments in age-matched mice from SLAM. The updated analysis of cytokine changes, **Fig. 3** and **Fig. 5b**, now reflect an increased sample size from n=30 (n=10 in each age group) to n=60 (n=20 in each age group). In the resulting analysis we identified 12 significant age-associated cytokines and 1 sex-associated cytokines (adjusted p-value < 0.05).

2) As the reviewer recognized, this study was not powered for identifying robust phenotypic changes and biomarker associations in SLAM. Instead, the study was designed to demonstrate a proteomic workflow that enables deep proteomic blood biomarker studies in mice in strictly volume limited conditions and showcase its application in the context of aging. The key strength/contribution of the study is that it introduces a method for *comprehensive* serum proteomic analysis in mouse cohorts, which are notably missing from existing literature due to the technical challenges associated with such studies. This workflow opens the door to comprehensive serum proteomic studies in mice even in volumes in low as 10 µL (11 µL for the addition of cytokines), a volume requirement that is compatible with longitudinal collection of blood in mice. This volume can also realistically be used for proteomics without consuming an entire serum volume and is thus compatible with studies that utilize blood for multiple different measurements (e.g. 'clinical' labs, proteomics, metabolomics, etc.). Although the current study is not powered to detect phenotypic changes, it is sufficiently powered to identify a larger number of age- and sex-associated proteins in serum than previously described (adjusted p-value < 0.05), reveal interesting aging biology, and potential clinical relevance, as evidenced by our addition of an additional analysis of a subset of protein changes that are conserved with humans (**Fig. 6**). In order to emphasize the strengths of the study in this framing, we have made the following adjustments:

page-14, line-424:

"Nonetheless, the NP-MS workflow demonstrated strong reproducibility and sensitivity, enabling robust detection of age- and sex--associated signatures. Importantly, this workflow was successful within strict sample volume requirements down to 10µL. Therefore, this method is compatible with the volume limitations of longitudinal mouse

studies and can be easily adapted and scaled into large cohort studies with a longitudinal study design. In future phases of the SLAM, incorporating larger sample sizes and true longitudinal sampling will be essential to validate these associations, improve statistical power and generalizability, and more accurately capture temporal relationships and potential causal effects across the lifespan."

We also completely agree that a larger study powered by the identification of aging-phenotype-associated biomarker signatures will be of ultimate interest. In fact, a separate, larger study powered for identification of phenotype-associated biomarker signatures is ongoing, and will ultimately demonstrate more detailed biomarker signatures, proteomic clocks, and multi-omic associations in a larger subset (n=900) of SLAM, with up to 6 longitudinal timepoints per animal. Because this is an ongoing, multi-team study that integrates multiple teams and datasets, it is outside the scope of the current manuscript. However, we have successfully completed the proteomic data acquisition for the larger SLAM study using the method described in this manuscript within 3 months, and with similar proteomic depth (>4000 cumulative proteins and ~2400 proteins per each 20ul serum sample), so we are confident in the scalability and applicability of the method described in this paper in a larger longitudinal mouse study.

To boost the significance of the current study, we would be happy to provide an additional figure summarizing the surface-level results of the larger SLAM study on request, including protein counts, list of protein/peptide identifications, and instrument time. These results would further demonstrate the scalability of this method and applicability in a longitudinal study for the current manuscript. However, given that the larger proteomic dataset is committed to an ongoing study, the underlying quantitative data from each sample would not be shareable at this time.

3) To reduce potential misunderstanding of current study might be longitudinal, we have changed wording in several places and have added paragraph into the discussion. Most notably, we have altered the title to highlight this as a cross-sectional analysis:

New Title: Deep and Quantitative Proteomic Profiling of Low Volume Mouse Serum: A Cross-Sectional Analysis of Aging

Discussion paragraph, page-14, line-424:

“Nonetheless, the NP-MS workflow demonstrated strong reproducibility and sensitivity, enabling robust detection of age- and sex--associated signatures. Importantly, this workflow was successful within strict sample volume requirements down to 10uL. Therefore, this method is compatible with the volume limitations of longitudinal mouse studies and can be easily adapted and scaled into large cohort studies with a longitudinal study design. Future phases of the SLAM, incorporating larger sample sizes and true longitudinal sampling will be essential to validate these associations, improve statistical power and generalizability, and more accurately capture temporal relationships and potential causal effects across the lifespan.”

Introduction-page-4, line-89:

“the proteomic data analyzed here derive from a cross-sectional subset of SLAM, with different mice sampled at 12 (young), 24 (old), and 30 (geriatric) months. Our intention is therefore not to infer within-individual aging trajectories but rather evaluate whether deep, quantitative serum proteomics can be achieved in a sample-limited context relevant to longitudinal aging studies”

Discussion-page-11, line-323:

“However, because the proteomic data derives from a cross-sectional subset of the longitudinal SLAM cohort, age-associated signatures reflect population-level differences rather than within- individual aging trajectories. All biological interpretations are therefore framed conservatively; and the results should be viewed as foundational and hypothesis-generating rather than mechanistic or causal.”

“Study Design” of the method section, page-15, line-463:

“The serum proteomics reported here sample a cross-sectional subset of SLAM, in which different mice were sampled at each age rather than repeatedly sampling the same individuals over time. Accordingly, the age-associated patterns reflect between-group differences, not within-individual longitudinal trajectories.”

4) As requested, we have removed and tempered claims based on modest trends and nominal statistics. We agree that these data risk overstating our findings and detract from

the primary contributions of the study as detailed in #2 above. The figure panels (formerly Fig. 5) and text pertaining to body fat & glucose have been removed accordingly. Additionally, the analysis of pairwise changes between young, old, and geriatric age groups has been removed with the exception of the Gene Set Enrichment Analysis (**Fig. 4**) showing significant pathways based on adjusted p-values. We thank the reviewer for this suggestion and strongly believe it has improved the focus of the manuscript.

3. The linear modeling linking proteomic data to glucose and fat percentage has very weak explanatory power. For glucose, the model yielded $R^2 = 0.018$ with $P = 0.57$ — nonsignificant and essentially no predictive capacity. For fat percentage, the model yielded $R^2 = 0.248$ with $P = 0.03$, which is statistically significant but still explains only a modest fraction of the variability. These low R^2 values indicate that the proteomic profiles account for little of the observed differences in these phenotypes. The manuscript currently overstates the strength of these associations; claims that proteomic changes robustly predict metabolic traits should be substantially tempered or removed.

Response: We thank the reviewer for this suggestion. As indicated in the previous response in more detail, we agree and have removed this data accordingly.

4. The manuscript repeatedly frames the study as "longitudinal" and references the NIA SLAM cohort. For example, the Introduction emphasizes the need for longitudinal biomarker assessment, and the Discussion states that the workflow is compatible with "longitudinal investigations." However, the actual dataset presented here is strictly cross sectional — 30 different mice sampled at three ages (12, 24, 30 months), with no repeated measurements within the same animals. This is a critical distinction. Cross-sectional comparisons cannot capture within-individual trajectories, causal relationships, or true lifespan dynamics. Therefore, the current analysis does not justify claims of "longitudinal" insights into aging biology. The title and framing of the manuscript are misleading and should be corrected. Unless longitudinal data are added, the manuscript's contribution to lifespan biology is fundamentally limited.

Response: We appreciate the reviewer for this important clarification regarding the distinction between longitudinal and cross-sectional designs. We fully agree that the dataset analyzed and reported in this manuscript is strictly cross-sectional, comprising 30 different mice sampled at three ages (12, 24, and 30 months), with no repeated measurements from the same individuals.

As such, the study cannot provide within-animal aging trajectories, causal relationships, or true longitudinal insights.

To address this, we have implemented several changes detailed in our second response to you above:

To reduce potential misunderstanding of current study might be longitudinal, we have changed wording in several places and have added paragraph into the discussion. Most notably, we have altered the title to highlight this as a cross-sectional analysis:

New Title: Deep and Quantitative Proteomic Profiling of Low Volume Mouse Serum: A Cross-Sectional Analysis of Aging

Discussion paragraph, page-14, line-424:

“Nonetheless, the NP-MS workflow demonstrated strong reproducibility and sensitivity, enabling robust detection of age- and sex--associated signatures. Importantly, this workflow was successful within strict sample volume requirements down to 10uL. Therefore, this method is compatible with the volume limitations of longitudinal mouse studies and can be easily adapted and scaled into large cohort studies with a longitudinal study design. Future phases of the SLAM, incorporating larger sample sizes and true longitudinal sampling will be essential to validate these associations, improve statistical power and generalizability, and more accurately capture temporal relationships and potential causal effects across the lifespan.”

Introduction-page-4, line-89:
“the proteomic data analyzed here derive from a cross-sectional subset of SLAM, with different mice sampled at 12 (young), 24 (old), and 30 (geriatric) months. Our intention is therefore not to infer within-individual aging trajectories but rather evaluate whether deep, quantitative serum proteomics can be achieved in a sample-limited context relevant to longitudinal aging studies”

Discussion-page-11, line-323:
“However, because the proteomic data derives from a cross-sectional subset of the longitudinal SLAM cohort, age-associated signatures reflect population-level differences rather than within-individual aging trajectories. All biological interpretations are therefore framed conservatively; and

the results should be viewed as foundational and hypothesis-generating rather than mechanistic or causal."

"Study Design" of the method section, page-15, line-463:
"The serum proteomics reported here sample a cross-sectional subset of SLAM, in which different mice were sampled at each age rather than repeatedly sampling the same individuals over time. Accordingly, the age-associated patterns reflect between-group differences, not within-individual longitudinal trajectories."

We now explicitly state that age-associated findings represent between group comparisons and should be interpreted accordingly. The manuscript also clarifies that the primary purpose of this study is to establish and benchmark a scalable NP-based proteomics workflow that will enable future longitudinal analyses when serial sample become available in subsequent phases of SLAM. Overall, we hope these revisions ensure accurate framing of the study and eliminate any ambiguity regarding the cross-sectional nature of the dataset.

5. One critical challenge associated with the use of NP-based strategies for biomarker discovery is the often observed lack of direct correlation between the proteins identified in the NP protein corona and those present in the plasma proteome. This discrepancy means that, even if proteomic variations are detected within the protein corona, additional validation using complementary analytical techniques [such as enzyme-linked immunosorbent assay (ELISA)] is essential to confirm the biological relevance and accuracy of these findings. Relying solely on proteomics data from the corona without validation can lead to misleading conclusions about potential biomarkers. Furthermore, it is imperative to consider the impact of aging on plasma composition, particularly regarding lipids, metabolites, and other small molecules. These factors can significantly influence the formation and composition of the protein corona on NPs, often more so than the plasma proteome itself. Unlike traditional proteomics methods, even minor alterations in lipid profiles or metabolic constituents can substantially modify the corona's molecular decoration, subsequently affecting proteomic outcomes without any apparent change in the plasma's overall protein composition. This dynamic interplay has been extensively documented in current literature, emphasizing that lipids and metabolites can modulate the affinity, binding patterns, and the overall structure of the corona, thereby influencing the detectability and interpretability of biomarkers.

Response: This is an important point, and we appreciate the importance of considering potential confounding effects on the composition of the protein corona, and the critical need to demonstrate the quantitative aging changes derived from the protein corona are representative of differences in the original serum samples. We have addressed and acknowledged these concerns with the inclusion of additional analysis and discussion. We hope the combination of responses presented below sufficiently address the underlying concerns of the reviewer.

First, we contend that the 'gold standard' method to demonstrate whether changes in the protein corona are representative of 'real' changes in the original serum samples at the proteome level (across a wide range of proteins) is to perform quantitative benchmarking (PMID: 27701404) on the same nanoparticles utilized in our study. This concern motivated us to conduct our own independent benchmarking study, shown in **Fig. 2A-B** (see below). Given that human and bovine blood are distinct in their compositions (including metabolites and lipids), if lipids/metabolites had a significant confounding effect on the composition of the protein corona, our expectation would be that our observed protein ratios would be skewed and inaccurate. However, our data suggests that on average the quantitative ratios of proteins in the protein corona are representative of true ratios of the original blood samples across a range of dilutions (5% - 100%).

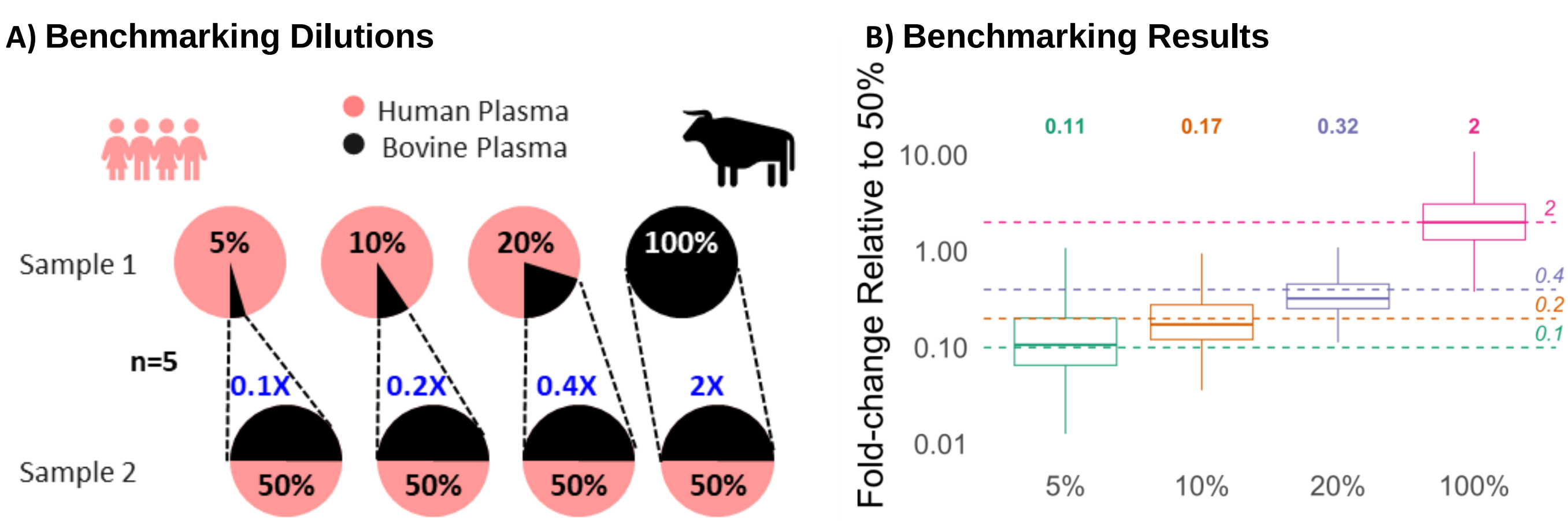

"Figure 2: Qualitative and quantitative evaluation of proteomic data generated NP-coupled MS workflow. A) A quantitative benchmarking study was performed using five fixed ratios of human and bovine plasma (n=5 each). B) Boxplot showing the measured peptide ratios in each dilution versus the 50% bovine plasma sample. Dotted lines depict the expected ratios."

Second, we emphasize that published literature, which has more rigorously and comprehensively examined characteristics of the protein corona and binding properties of the nanoparticles utilized in this study, suggests that the effect of lipids and matrix do not significantly impact quantitation. We note that the nanoparticles used in this study are distinct from the plain polystyrene nanoparticles in the study cited by Reviewer #1 (PMID: 39511193) and may not exhibit the same binding behavior. The surface-functionalized superparamagnetic iron nanoparticles in this study are characterized in more detail elsewhere (PMID: 35275789). Lipid spike-in experiments have been done on the same nanoparticles we used, showing that spiking varying amounts of lipid do not significantly affect the number/intensity of proteins (PMID: 32699280, Nature Comms - last paragraph of p5 & first on p6, Fig. S4 and S5). Another paper in JPR (PMID: 41105214) reported on the minimal impact of background matrix (presumably with different lipid/metabolite compositions) on quantitative accuracy. We have noted these additional studies in the discussion (quoted below).

We explained now in the revised "Discussion" section for more clarity, page-10, line-295:

"Concerns have been raised that NP- coronas may be sensitive to non-protein serum components, such as lips or metabolites, potentially introducing variability unrelated to biological differences. While recent work has shown that small molecules can influence protein corona composition derived from plain polystyrene nanoparticles (40), multiple independent studies of the surface-functionalized superparamagnetic iron nanoparticles employed in this study suggest that these effects are not significant. Lipid-spike-in experiments using the same nanoparticles showed no appreciable changes in corona protein identities or intensities (15). Similarly, a prior report suggests minimal impact of background matrix on quantitative accuracy (41). Our own quantitative benchmarking data further supports this conclusion, showing high reproducibility and accurate quantification of known fold-changes in samples generated from mixing human and bovine blood, each containing a distinct composition of lipids, metabolites, and other components, in a range of fixed dilutions spanning 5% to 100% (**Fig. 2B**). Together, these findings indicate that matrix-derived components did not significantly alter corona composition in a manner that distorts relative protein quantification in this study and mitigate concerns matrix effects introduced variability unrelated to aging."

Third, we attempted to address the reviewer's request for independent validation of the aging changes measured in protein coronas with additional analysis and comparison with literature.

Targeted cytokine measurements with the Proximity Extension Assay, and antibody-affinity-based platform, were a major component of this study. To perform a cross-platform comparison of a protein measured by both methods, nanoparticle-based MS versus antibody-based, we searched for overlapping protein observations between datasets and found a single protein – IL16 – with enough observations across mice to perform a comparison (**Fig. 3C-D**, 11 mice with matched measurements in both platforms). Using linear modeling, we found significant correlation between platforms in the measurement of IL16 (p=0.0026, R = 0.81). This example further supports our claim of accurate representation of protein levels in the protein corona when compared with the original serum samples. To further validate whether our observed changes align with expected biology and proteomic measurements from prior studies, we performed an analysis comparing our measured changes to two published MS-based proteomic datasets in mouse blood that did not use nanoparticles. Although there are no prior mouse aging studies in blood to draw from, the two published studies examined sex-associated changes in mouse serum (PMIDs 37910662 and 34050187). Notably, with the exception of two proteins out of thirty-two (Transcobalamin-2 and Hepatocyte growth factor activator), all sex-associated proteins from our study that were also reported in either published study were directionally aligned and correlated. This has been added as a supporting figure, **Fig. S3**, and is numerically detailed in **Table S4**. Overall, these findings further support that protein corona measurements in our study align with direct measurements in blood samples and accurately reproduce expected biological changes, such as sex-dimorphism.

New figure, **Fig. S3**:

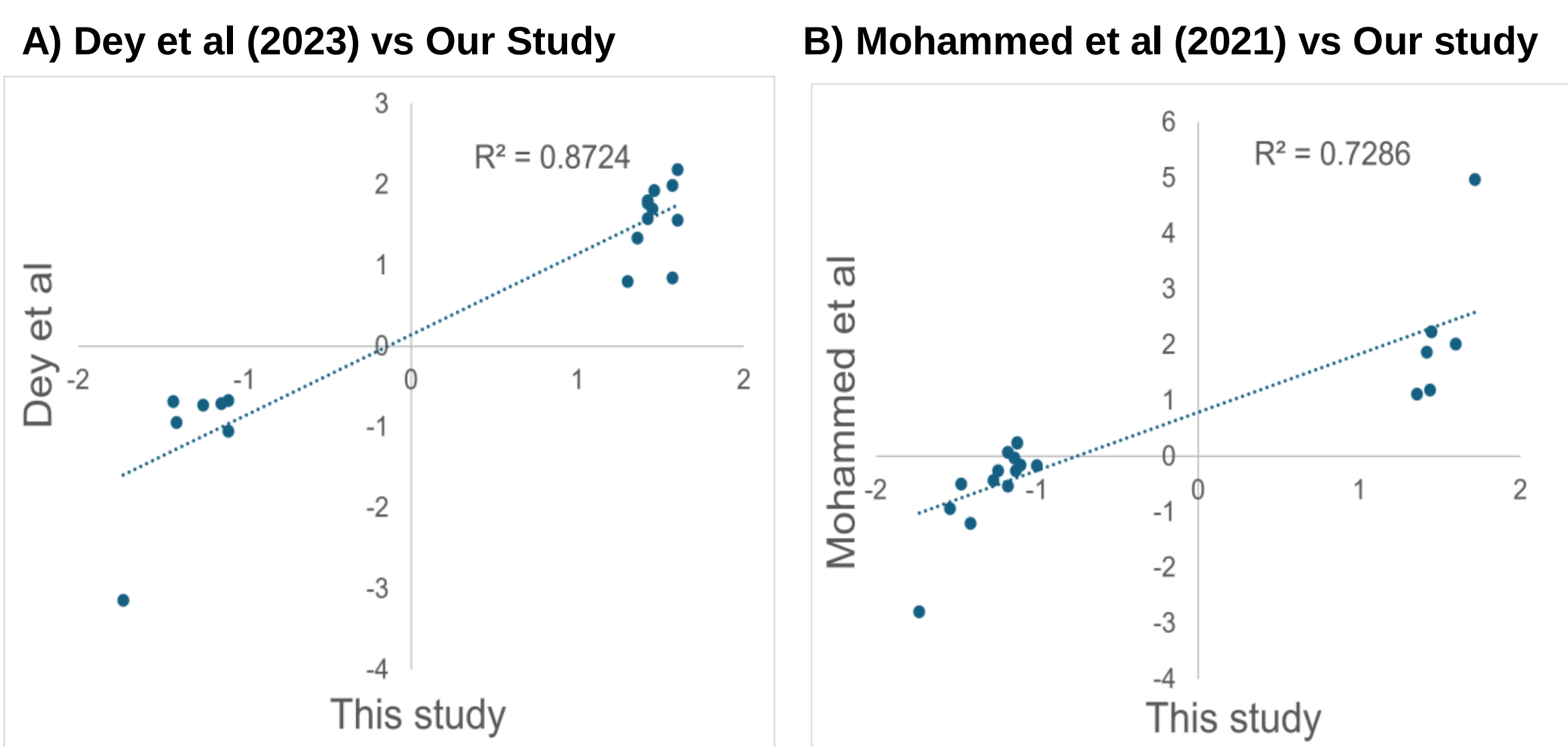

“**Supplementary Fig 3. Correlation of sex-associated circulating proteins between this study and published datasets.** Correlation plots depicting sex-associated changes in our study versus those reported in A) Dey et. al. (PMID: 37910662); and B) Mohammed et. al. (PMID- 34050187). Proteins in both studies show strong positive correlations with this study (R^2=0.8724 and R^2=0.7286, respectively), and consistent directionality of sex associated effects.”

Supplemental Table 4: Validation of top sex associated proteins with other study

				**SLAM**	**Dey et. al. JPR,2023**	**Mohammed et. al. NPJ Syst. Biol. Appl. 2021**
**Sl. No.**	**Gene**	**Uniprot ID**	**Description**	**B-sex**	**log2(M/F)**	**log2(M/F)**
1	Serpina1e*	Q00898	Alpha-1-antitrypsin	**1.72**		**4.97**
2	C8g*	Q8VCG4	Complement component C8 gamma chain	**1.6**	**1.56**	**2.02**
3	C6	E9Q6D8	Complement component 6	**1.6**	**2.19**	
4	C7	D3YXF5	Complement component 7	**1.57**	**1.99**	
5	C8b*	Q8BH35	Complement component C8 beta chain	**1.45**	**1.7**	**2.24**
6	C8a*	Q8K182	Complement component C8 alpha chain	**1.42**	**1.77**	**1.87**
7	Ces3a	Q63880	Carboxylesterase 3A	**1.57**	**0.85**	
8	Mug1*	P28665	Murinoglobulin-1	**1.44**		**1.19**
9	Mup20	Q5FW60	Major urinary protein 20	**1.46**	**1.93**	
10	Crisp1	Q03401	Cysteine-rich secretory protein 1	**1.42**	**1.8**	

11	Mup3	P04939	Major urinary protein 3	1.42	1.58	
12	Egfr*	Q01279	Epidermal growth factor receptor	1.36	1.34	1.12
13	Serpina3k	P07759	Serine protease inhibitor A3K	1.3	0.8	
14	Tcn2	O88968	Transcobalamin-2	-1.12		0.25
15	Glycam1*	Q02596	Glycosylation-dependent cell adhesion molecule 1	-1.73	-3.14	-2.79
16	Adipoq*	Q60994	Adiponectin	-1.54		-0.94
17	Bche	Q03311	Cholinesterase	-1.47		-0.5
18	Serpina6*	Q06770	Corticosteroid-binding globulin	-1.41	-0.94	-1.2
19	Scg3	P47867	Secretogranin-3	-1.43	-0.68	
20	Ctsc	P97821	Dipeptidyl peptidase 1	-1.1	-1.05	
21	Afm	O89020	Afamin	-1.18		-0.53
22	Gsn	P13020	Gelsolin	-1.1		-0.16
23	Hgfac	Q9R098	Hepatocyte growth factor activator	-1.18		0.07
24	Igfals	P70389	Insulin-like growth factor-binding protein complex acid labile subunit	-1.27		-0.43
25	Itih1	Q61702	Inter-alpha-trypsin inhibitor heavy chain H1	-1.14		-0.03
26	Itih2	Q61703	Inter-alpha-trypsin inhibitor heavy chain H2	-1.13		-0.26
27	Itih4	A6X935	Inter alpha-trypsin inhibitor, heavy chain 4	-1		-0.17

28	Lum	P51885	Lumican	**-1.24**		**-0.26**
29	Psmb4	P99026	Proteasome subunit beta type-4	**-1.12**		**0.24**
30	Pi16	Q9ET66	Peptidase inhibitor 16	**-1.1**	**-0.67**	
31	Retn	Q99P87	Resistin	**-1.25**	**-0.73**	
32	Igfbp5	Q07079	Insulin-like growth factor-binding protein 5	**-1.14**	**-0.71**	

Red- Higher in males; **Blue**- Higher in Females; *-97% (C-statistics) discrimination between males and females (Mohammed et. al. 2021)

6. The addition of the 48-plex cytokine panel is a great complement to the MS data, given MS's difficulty in detecting many cytokines. However, the cytokine panel is limited to the targets included in that assay, meaning some relevant inflammatory markers could still be missing if not on the panel. The overlap between MS and PEA was small (only 7 proteins detected by both, according to the text), indicating that each method captures distinct subsets of the proteome. This underscores the importance of using both; however, the data from the two platforms are largely presented separately. Recommendation: The authors could consider a more integrated analysis or discussion of how the two datasets together inform the biological story. For instance, do the cytokines uniquely detected by PEA show any coherent pattern with age or sex (some of this is touched on in Fig.3 and Fig.5B)? Highlighting any such insights would further demonstrate the value of the combined approach. Additionally, to enhance clarity, the manuscript should clearly state the identity of the cytokine panel (e.g. PEA technology by a specific provider) and confirm if results from the two methods were normalized or analyzed independently. Minor point: including a brief mention of any quality control for the PEA assay (e.g. duplicates, standard curves, or limit-of-detection considerations) would be reassuring.

Response: We thank the reviewer for thoughtful and constructive feedback regarding the complementary cytokine panel and the opportunity to more fully integrate insights from the MS and proximity extension assay (PEA) datasets. We agree that MS and PEA capture distinct but biologically meaningful subsets of the circulating proteome and that emphasizing their combined

interpretive value strengthens the manuscript. The small overlap between MS and PEA (4 proteins) resulting in complementary views of the circulating proteome that includes many important, but not all, cytokines that are traditionally missed by MS.

We appreciate the reviewer's recommendation to further integrate the two proteomic datasets and agree it would further highlight their complementarity. In a newly introduced figure that assesses the overlap of these findings with aging changes in human blood (**Fig. 6**), we have integrated our analysis to include both datasets. Of the 18 age-associated proteins conserved in humans, 2 were from PEA and 16 were from MS (**Fig. 6A,B,D**). Given the small number of significant changes by PEA for the 30 mouse samples that have matched analysis in both PEA and MS (four proteins adjusted p-value < 0.05), we are somewhat limited in the additional insight that can be gained from integration. At the level of biology, we view PEA cytokine panel as an independent targeted analysis of the inflammatory state of the organism that the MS dataset does not necessarily reflect due, but due to the targeted nature of the assay, there are some limitations to the integration (e.g. integrated pathway analysis, since the background protein list is entirely a single pathway - an inflammatory cytokine panel). However, we modified the discussion to include integrated speculation/insights and discuss integration of data types. page-11, line343:

"Although overlap between MS and PEA was limited (4 proteins, **Fig. 3C**), reflecting the fundamentally different detection principles and sensitivity ranges of the two platforms. However, cytokines uniquely detected by PEA exhibited coherent age-associated patterns including increases in 10 cytokines, including known SASP factors Il6, Tnf, Csf3, Csf1, and Ccl2. Together with the observed increases in SenSig proteins and immune cell activation pathways by MS-based proteomics, these results support an age-related increase in senescence-driven immune cell activation in circulation. The PEA panel provides an independent assessment of the inflammatory state of the organism based on inflammatory cytokines that are missed by traditional unbiased MS assays."

To enhance clarity, we now explicitly state in the "Methods" that cytokines were quantified using Olink Target 48 Mouse Cytokine Panel, which is based on Proximity Extension Assay (PEA) technology. We also clarify that PEA and MS datasets were processed and normalized independently using their respective platform-specific pipelines, reflecting differences in dynamic range, detection chemistry, and quantification scales. Finally, we have added a brief description of PEA quality control procedures, page-18, line-558:

“Cytokines in the mouse serum samples were quantified using the Olink Target 48 Mouse Cytokine panel based on Proximity Extension Assay (PEA) technology (Olink Proteomics. Assays were performed by Assay Gate Inc. (Ijamsville, MD) according to manufacturer’s instructions. PEA and MS datasets were processed and normalized independently using platform-specific pipelines due to differences in assay chemistry and dynamic range. Quality control assessments of the PEA assay include inclusion of process controls (for key protocol steps – incubation, extension, and detection), a triplicate sample control to monitor intra-run precision, duplicate negative controls, and triplicate calibrator samples for normalization between batches (77).The limits of detection and quantification, and precision of each protein measurement have been previously reported (77).” All samples used for this study passed internal and external quality control metrics.

Some major questions that need to be addressed:

1) Why emphasize MaxRep when MaxLFQ is more established and less biased? Can the authors provide statistical evidence for superior reproducibility?

Response: We appreciate the reviewer’s question regarding our emphasis on the MaxRep roll-up approach. We believe both approaches are valid, but in our hands the MaxRep approach worked marginally better. MaxRep is based on empirical performance in this specific NP-fractionated workflow, where each peptide is quantified using its highest intensity peptide–nanoparticle observations, and was widely adopted as the default quantification approach for these nanoparticle kits until recently. However, as the reviewer points out, MaxLFQ is indeed a widely used and well-established method for peptide-to-protein quantification in traditional MS studies, and for this reason we compare both MaxRep and MaxLFQ directly within our dataset in the context of an NP-based workflow.

In our comparison of each approach, we quantified similar number of proteins (3992 proteins) in each. However, in our analysis of reproducibility based on the CVs of protein-level quantification (**Fig. 2J**), MaxRep demonstrated slightly superior reproducibility, with a lower median protein-level coefficient of variation (CV = 0.0567) compared to MaxLFQ (CV = 0.0613). MaxRep also yielded a modestly higher proportion of proteins with CV < 10%, reflecting tighter quantitative precision under our experimental conditions. These results imply that MaxRep can marginally reduce cross-nanoparticle variability by using the most consistently observed and highest-

intensity peptide measurement for each protein. Importantly, the quantitative accuracy of the MaxRep approach was already corroborated in our quantitative benchmarking study (**Fig. 2B**) before the MaxRep/MaxLFQ comparisons were performed.

To reflect the reviewer's concern about potential bias, we note in the revised text, page-7, line-193:

"Accordingly, MaxRep was used for primary analyses due to its marginally improved reproducibility, while downstream biological conclusions were robust to roll-up choice."

2) The study identifies >70 age-associated proteins. Which of these are validated against prior murine or human aging proteomics datasets?

Response: We appreciate the suggestion and agree that comparison with large-scale human and mouse proteomics datasets is important to establish confidence in the data and that mouse serum proteomics can inform human aging biology. With regard to mouse data – there are no comprehensive proteomic studies of aging to draw from to our knowledge, but several published MS studies did evaluate sex-specific protein changes in mouse serum that we could use to assess whether our study reproduces 'known' biology. We discuss these comparisons in detail in the next response to your question about comparing sex-dimorphic signatures with existing literature.

To address the comparison with existing human aging data, the revised manuscript incorporates a cross- species comparison between the mouse proteomic signatures identified in this study and human plasma proteomics from Baltimore Longitudinal Study of Aging (BLSA) and published UK Biobank proteomics that support the translational relevance of the mouse data.

The newly generated analysis has been included in the results as a main figure (**Fig. 6**). Our analysis reveals that 50% of the age-associated proteins identified in this mouse study are also significantly age-associated in the BLSA. Interestingly, these results suggest a common circulating aging signature of extracellular matrix, endoplasmic reticulum, and lysosomal protein components in humans and mice. We assessed the association of these proteins with frailty, a key clinical aging endpoint, in BLSA and found that five significantly associated with frailty: COL6A3, LBP, PIGR, IGFBP1 and HPX. By far, the ECM protein COL6A3 is the most significant among these (adj. p-value = 5.07e-16). These findings are consistent with recent evidence of age-associated increase in ECM components in Nature Aging (PMID: 41023316), and a broad range

of literature associating COL6A3 (aka Endotrophin, the uncleaved precursor) with mortality and chronic diseases (PMID 38871093). Notably, COL6A3 is among the top 5 proteins associated with pre-frailty, and frailty, in the UK Biobank (PMID 39535692), independently validating the association with human frailty observed in BLSA. Overall, these results support clinical relevance of the findings of our study and demonstrate the mouse study can shed light on human aging. We have included the new analysis into the results, figures, and discussion, accordingly, as shown below.

New Figure, **Fig. 6**:

New analysis described in the Results section, page-9, Line-253:

"To assess cross-species concordance, we compare age-associated circulating proteins in mice with human plasma proteomics from the BLSA (35, 36). Among 1742 proteins quantified in both datasets, Pearson Linear modeling (age ~ protein + sex) identified 18 proteins, representing 5% of the total proteins or 50% of mouse age-associated proteins, to be significantly (**Fig. 6A**) and positively (Fig. 6B) associate with age in both mice and humans. Overrepresentation analysis of shared upregulated proteins with age in both mice and humans revealed enrichment for cellular component categories (Fig. 6C) including endoplasmic reticulum, extracellular matrix, and lysosomal proteins, among others, and suggesting shared categories of age- associated circulating factors at a pathway-level. To assess the relevance of these findings with age-associated clinical phenotypes, we evaluated the associations of these proteins with frailty index, a 44-component composite score, in the BLSA. Notably, five proteins including COL6A3, LBP, PIGR, and IGFBP1, and HPX were significantly associated with frailty index in the BLSA (Frailty Index ~ Protein + age + sex; **Fig. 6D**). Collectively, these results link conserved proteomic aging signatures to functional health decline in humans and suggest that aging-associated biomarkers derived from mice may be relevant in humans."

We have emphasized these cross-species findings in the Abstract, page-2:

"Cross-species comparison with human plasma proteomics from the Baltimore Longitudinal Study of Aging (BLSA) identified conserved age-associated circulating proteins, several of which were linked to frailty in humans."

Finally, we elaborated on the significance of these results in the Discussion, page-13, line 394:

“To place the mouse findings in a translational context, we compared age-associated circulating proteins identified here with human plasma proteomics from BLSA. Despite differences in species, cohort design, and analytical platforms, a subset of proteins, representing half of the mouse age-associated proteins, were concordantly age- associated in humans (Fig. 6A, 6B), indicating conserved features of the circulating aging proteome. Interestingly, collagen-containing extracellular matrix proteins were overrepresented among these, consistent with recent reports of the association of extracellular matrix components, including collagen, with aging in mouse circulation (66). Moreover, several of these shared proteins, including COL6A3, LBP, PIGR, IGFBP1, and HPX, were also significantly associated with frailty in humans, linking shared circulating proteomic signatures to functional health decline. Notable among these, Collagen Type VI Alpha 3 Chain (COL6A3, a.k.a. Endotrophin), a well-known indicator of fibrotic aging, was by far the most significant frailty-associated protein in the BLSA (adjusted p-value = 5.069e-16). COL6A3 has been associated with mortality and a range of non-communicable chronic diseases linked to aging, including idiopathic pulmonary fibrosis, chronic kidney disease, type 2 diabetes, cardiac dysfunction, among others (67). Remarkably, COL6A3 is also among the top five proteins associated with pre-frailty and frailty in the UK Biobank (68), independently validating the robustness of this finding in humans. Although exploratory, these findings collectively support the translational relevance of mouse serum proteomics and suggest that at least a subset of aging-associated circulating proteins is conserved in humans. While we expect the correlation of mouse and human age-associated proteins as a whole to be limited due to the substantial influence of genetics and other factors on the circulating proteome, we speculate that a key subset of circulating proteins with mechanistic links to biology will be conserved and translationally relevant. Notable among these in our study are robust aging- and disease-associated proteins such as COL6A3 and lipopolysaccharide-binding protein (LBP), and sex-associated proteins such as Complement 8 (conserved association with the male sex) and adiponectin (ADIPOQ, conserved association with females).”

3) How do the sex-dimorphic signatures align with existing literature? Are these findings reproducible in independent datasets?

Response: We appreciate the reviewer’s question regarding the biological validity and reproducibility of the sex-dimorphic protein signatures. To further validate whether our observed changes align with expected biology and proteomic measurements from prior studies, we

performed an analysis comparing our measured changes to two published MS-based proteomic datasets in mouse blood that did not use nanoparticles. Although there are no prior mouse aging studies in blood to draw from, the two published studies examined sex-associated changes in mouse serum (PMIDs 37910662 and 34050187). Notably, apart from two proteins (Transcobalamin-2 and Hepatocyte growth factor activator) out of thirty-two, all sex-associated proteins from our study that were also reported in either published study were directionally aligned and correlated. This has been added as a supporting figure, **Fig. S3**, and is numerically detailed in Table S4. Overall, these findings further support that protein corona measurements in our study align with direct measurements in blood samples and accurately reproduce expected biological changes, such as sex-dimorphism.

New figure, **Fig. S3**:

A) Dey et al (2023) vs Our Study **B) Mohammed et al (2021) vs Our study**

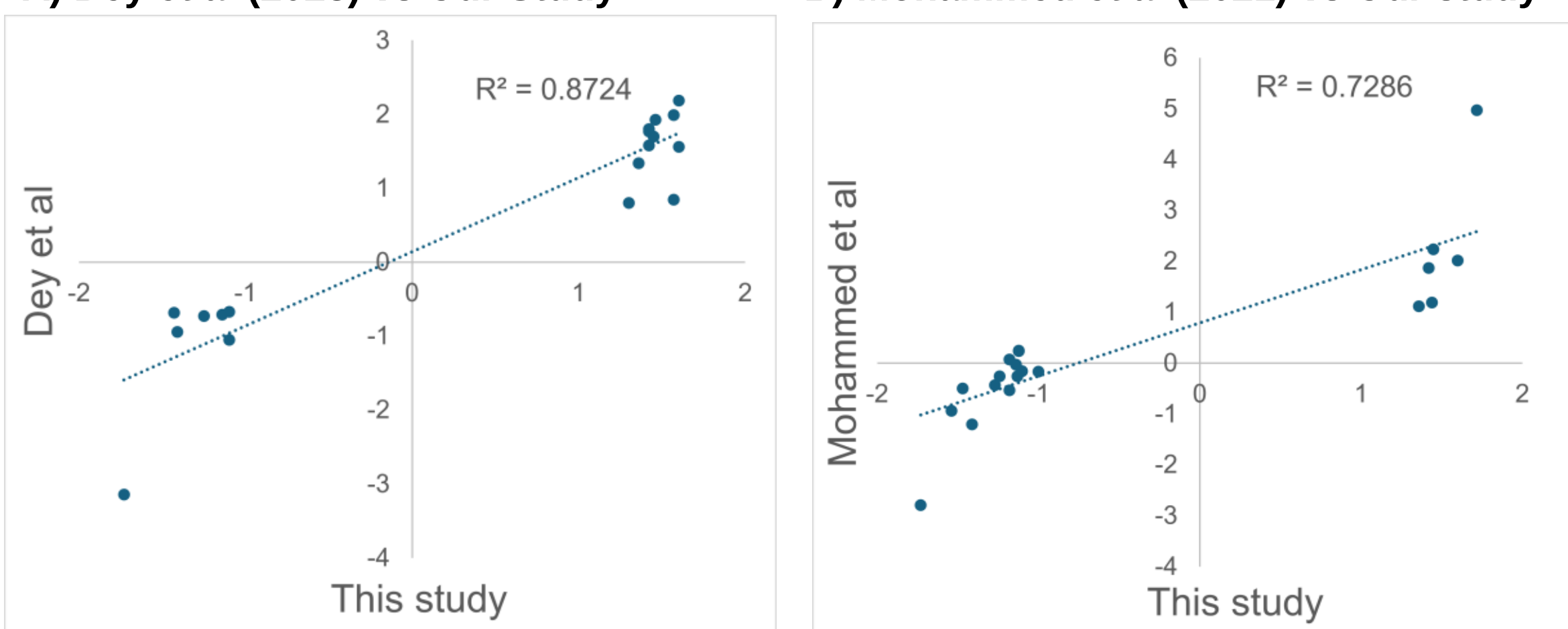

"Supplementary Fig. 3- Correlation of sex-associated circulating proteins between this study and published datasets. Correlation plots depicting sex-associated changes in our study versus those reported in A) Dey et. al. (PMID: 37910662); and B) Mohammed et. al. (PMID- 34050187). Proteins in both studies show strong positive correlations with this study (R^2=0.8724 and R^2=0.7286, respectively), and consistent directionality of sex associated effects."

Supplemental Table 4: Validation of top sex associated proteins with other study

	SLAM	Dey et. al. JPR,2023	Mohammed et. al. NPJ Syst.

Sl. No.	Gene	Uniprot ID	Description	B-sex	log2(M/F)	Biol. Appl. 2021 log2(M/F)
1	Serpina1e*	Q00898	Alpha-1-antitrypsin	1.72		4.97
2	C8g*	Q8VCG4	Complement component C8 gamma chain	1.6	1.56	2.02
3	C6	E9Q6D8	Complement component 6	1.6	2.19	
4	C7	D3YXF5	Complement component 7	1.57	1.99	
5	C8b*	Q8BH35	Complement component C8 beta chain	1.45	1.7	2.24
6	C8a*	Q8K182	Complement component C8 alpha chain	1.42	1.77	1.87
7	Ces3a	Q63880	Carboxylesterase 3A	1.57	0.85	
8	Mug1*	P28665	Murinoglobulin-1	1.44		1.19
9	Mup20	Q5FW60	Major urinary protein 20	1.46	1.93	
10	Crisp1	Q03401	Cysteine-rich secretory protein 1	1.42	1.8	
11	Mup3	P04939	Major urinary protein 3	1.42	1.58	
12	Egfr*	Q01279	Epidermal growth factor receptor	1.36	1.34	1.12
13	Serpina3k	P07759	Serine protease inhibitor A3K	1.3	0.8	
14	Tcn2	O88968	Transcobalamin-2	-1.12		0.25
15	Glycam1*	Q02596	Glycosylation-dependent cell adhesion molecule 1	-1.73	-3.14	-2.79
16	Adipoq*	Q60994	Adiponectin	-1.54		-0.94

17	Bche	Q03311	Cholinesterase	**-1.47**		**-0.5**
18	Serpina6*	Q06770	Corticosteroid-binding globulin	**-1.41**	**-0.94**	**-1.2**
19	Scg3	P47867	Secretogranin-3	**-1.43**	**-0.68**	
20	Ctsc	P97821	Dipeptidyl peptidase 1	**-1.1**	**-1.05**	
21	Afm	O89020	Afamin	**-1.18**		**-0.53**
22	Gsn	P13020	Gelsolin	**-1.1**		**-0.16**
23	Hgfac	Q9R098	Hepatocyte growth factor activator	**-1.18**		**0.07**
24	Igfals	P70389	Insulin-like growth factor-binding protein complex acid labile subunit	**-1.27**		**-0.43**
25	Itih1	Q61702	Inter-alpha-trypsin inhibitor heavy chain H1	**-1.14**		**-0.03**
26	Itih2	Q61703	Inter-alpha-trypsin inhibitor heavy chain H2	**-1.13**		**-0.26**
27	Itih4	A6X935	Inter alpha-trypsin inhibitor, heavy chain 4	**-1**		**-0.17**
28	Lum	P51885	Lumican	**-1.24**		**-0.26**
29	Psmb4	P99026	Proteasome subunit beta type-4	**-1.12**		**0.24**
30	Pi16	Q9ET66	Peptidase inhibitor 16	**-1.1**	**-0.67**	
31	Retn	Q99P87	Resistin	**-1.25**	**-0.73**	
32	Igfbp5	Q07079	Insulin-like growth factor-binding protein 5	**-1.14**	**-0.71**	

Red- Higher in males; **Blue**- Higher in Females; *-97% (C-statistics) discrimination between males and females (Mohammed et. al. 2021)

The convergence with multiple external datasets provides confidence that the identified sex differences reflect true, biologically conserved features of the circulating proteome rather than cohort- or method-specific artifacts.

4) Cytokine panel results show limited overlap with MS. Could this reflect assay limitations, matrix effects, or biological absence? Provide a critical evaluation.

Response: We appreciate the reviewer's insightful question regarding the limited overlap between cytokines detected by MS and those quantified by the PEA cytokine panel. This discrepancy is expected and reflects a combination of biological, technical, and matrix-dependent factors inherent to each platform. In MS proteomics, it is known that inflammatory cytokines are commonly missed due to a lack of unique tryptic peptides with adequate ionization efficiency for detection, so we expected these measurements to be lacking in our MS analysis. This was our primary motivation for selecting a targeted mouse cytokine panel, so our results were in line with this expectation. In matrices such as blood, as the reviewer points out, this challenge is further compounded because the abundance of cytokines tends to be at the bottom end of the vast dynamic range of protein concentrations spanning 10+ orders of magnitude, thus we reasoned that a highly sensitive affinity-based based approach with a demonstrated capability to detect these proteins in blood (PEA/Olink) would be an ideal complimentary assay.

In the "Introduction" section of the manuscript (page-3, line- 74) we discuss that MS-based detection of cytokines is constrained by small protein size, limited tryptic peptides, and poor ionization efficiency, whereas PEA directly targets proteins with high analytical sensitivity and minimal matrix interference. Conversely, PEA is inherently limited to the 48 predefined targets included on the panel; cytokines or inflammatory mediators not represented cannot be detected.

In the revised manuscript, we highlight that the two platforms together provide a more complete view of the circulating inflammatory landscape than either could achieve alone- page-11, line-343:

"Although overlap between MS and PEA was limited (4 proteins, **Fig. 3C**), reflecting the fundamentally different detection principles and sensitivity ranges of the two platforms. However, cytokines uniquely detected by PEA exhibited coherent age-associated patterns including increases in 10 cytokines, including known SASP factors Il6, Tnf, Csf3, Csf1, and Ccl2. Together with the observed increases in SenSig proteins and immune cell activation pathways by MS-

based proteomics, these results support an age-related increase in senescence-driven immune cell activation in circulation. The PEA panel provides an independent assessment of the inflammatory state of the organism based on inflammatory cytokines that are missed by traditional unbiased MS assays."

5) Several biological conclusions (e.g., links to metabolic health) are speculative. Can authors provide mechanistic validation or stronger evidence?

Response: We agree that data related to metabolic outcomes (e.g. glucose/fat associated proteins) were limited. In line with your earlier suggestions to temper or remove the statistically marginal results, we removed much of the analysis and re-focused the manuscript on the strengths of the study. This is described in more detail in our response to your second 'main' comment. Our discussion now focuses more on the advantages of this proteomic workflow and more statistically definitive results, such as the age-and sex-associated proteins.

6) What measures were taken to ensure randomization and avoid bias in sample preparation and acquisition?

Response: Several measures were implemented to ensure that sample processing and MS acquisition were not confounded by batch effects or systematic bias.

a) **Sample processing:** To minimize batch effects and procedural bias, serum samples were block-randomized or randomized under the constraint that samples of each age- and sex-are equally distributed across 96-well plates on the automated SP100 platform. Each plate/batch contains process controls to account for technical variability and system suitability, as well as sample controls to assess inter-plate performance. The automated sample processing is performed on one plate at a time, where samples are treated in parallel with the same five distinct NPs under identical incubation and workflow conditions, ensuring uniform handling and minimizing systematic technical variation during protein corona formation, digestion, and peptide cleanup. This randomized and balanced processing strategy has now been described in the Methods section of revised manuscript- page-16, line-489:

“All serum samples were processed using the Seer Proteograph workflow on an automated SP100 instrument [15, 29], employing a randomized and age- and sex-balanced 96- well plate design to minimize batch effects and procedural bias.”

Page-16, line-507:

“All samples were processed under identical workflow conditions using an automated workflow, ensuring uniform handling and minimizing technical variability during protein corona formation, digestion, and peptide clean-up.

b) **Block-randomized LC–MS acquisition:** As noted in the Methods, DIA-MS runs were acquired in a block-randomized order (already included in the method section- page-17, line-536), which distributes samples from each age and sex group across the acquisition sequence and minimizes potential bias related to instrument drift, carryover, or temporal variation in MS performance.

c) **Use of technical replicates in feasibility and quantitative benchmarking:** For the NP reproducibility and fold accuracy benchmarking experiment, technical triplicates were processed and acquired independently, confirming low CVs and limiting operator- or batch-related bias.

Together, these steps ensured that sample preparation and acquisition were unbiased with respect to age and sex groups, and that downstream quantitative comparisons reflected biological rather than procedural differences.

7) Given the small sample size, what is the risk of false discovery? How do the authors plan to validate these biomarkers in larger cohorts?

Response: We agree that modest sample size increases the risk of false discovery, particularly for subtle or non-linear age-associated changes. To mitigate this, we applied stringent statistical controls, including peptide/protein FDR at a 1%, and we interpreted all findings as hypothesis-generating rather than definitive. As outlined in the revised manuscript, candidate biomarkers identified will be validated in larger cohorts through upcoming phases of the SLAM study, which will include substantially greater mouse numbers and true longitudinal sampling, as well as

through targeted assays such as ELISA, multiplex immunoassays, or targeted MS to confirm reproducibility and biological relevance.

As noted above, a separate, larger study powered for identification of phenotype-associated biomarker signatures is ongoing, and will ultimately demonstrate more detailed biomarker signatures, proteomic clocks, and multi-omic associations in a larger subset (n=900) of SLAM, with up to 6 longitudinal timepoints per animal. Because this is an ongoing, multi-team study that integrates multiple teams and datasets, it is outside the scope of the current manuscript. However, we are happy to share that the proteomic data acquisition for the larger SLAM study was completed using the method described in this manuscript within 3 months, and with similar proteomic depth (>4000 cumulative proteins and ~2400 proteins per each 20µl serum sample), so we are confident in the scalability and applicability of the method described in this paper in a larger longitudinal mouse study.

To boost the significance of the current study, we would be happy to provide an additional figure summarizing the surface-level results of the larger SLAM study on request, including protein counts, list of protein/peptide identifications, and instrument time. These results would further demonstrate the scalability of this method and applicability in a longitudinal study for the current manuscript. However, given that the larger proteomic dataset is committed to an ongoing study, the underlying quantitative data from each sample would not be shareable at this time.

Some minor comments:

1) Abstract should clarify that cytokines were primarily detected by PEA, not MS.

Response: Thank you and clarified – page-2, line-40:
"With 1 µL additional serum, a 48-plex Olink Proximity Extension Assay (PEA) quantified 39 cytokines not detected by MS."

2) Authors should provide at least general information about nanoparticle properties (like charge, size) instead of only calling them 'proprietary'.

Response: We appreciate the reviewer's suggestion for additional information regarding NPs properties. While the exact formulations, sizes, and surface chemistries of the NPs used in the

Proteograph workflow are proprietary. Although we agree that providing general physicochemical context might increase transparency and interpretation.

Proteograph workflow employs a panel of engineered nanoparticles with diverse physicochemical properties, including variation in surface charge, hydrophobicity, and functional chemistry, as described previously (PMID: 32699280). These NPs are based on superparamagnetic iron oxide nanoparticles (SPION) cores enabling automated magnetic capture and washing steps. Although specific dimensions are not disclosed, the particles fall within the typical size range (SEM data showed three SPIONs core are 200–300 nm with spherical and semi-spherical morphologies) used for synthetic protein capturing nanomaterials. Notable, the heterogeneity in surface characteristics is designed to promote formation of distinct, and reproducible protein coronas, allowing selective enrichment of proteins spanning a wide dynamic range. This corona formation effectively reduces sample complexity, enhances detection of low-abundance proteins, and supports deep proteome coverage in downstream MS analysis.

We have added a brief description of the NP to the Methods section, to provide greater transparency while acknowledging proprietary constraints, page-16, line-491:

“The workflow uses a subset of engineered NPs with diverse surface chemistries, including variations in charge and functional groups, built on superparamagnetic iron oxide nanoparticle (SPION) cores to enable magnetic capture, as previously described (15). Although the exact characteristics of the commercially available NP kits are proprietary, they are selected from among the library of NPs characterized previously characterized in more detail (15). Each NP forms distinct and reproducible protein coronas that, when used alone or in combination, enhance proteome depth.”

Recommendation: Major revision While this manuscript presents an interesting application of nanoparticle-assisted proteomics to low-volume mouse serum, it suffers from critical design, statistical, and methodological weaknesses that, in my point of view, preclude publication in journals that requires robust data presentation and novelty such as Nature Aging.

Reviewer #3 :

This study introduces and validates a nanoparticle-based, data-independent acquisition (DIA) mass-spectrometry workflow that enables deep and quantitative proteomic profiling of low-volume mouse serum across the lifespan, complemented by a proximity extension assay for cytokines

(Olink). The authors identified around 4,000 proteins including >200 senescence-associated markers, and, using 1 µL of additional serum, quantified 39 further cytokines typically missed by MS. The quantitative accuracy was high (median CV < 10%) and strong agreement between NP fractions was reported. The authors applied the pipeline to 30 mice aged 12, 24 and 30 months and revealed age- and sex-dependent proteomic signatures and correlations with glucose and body fat. The paper demonstrates a good level of benchmarking, and the complementary use of Olink to capture low-abundance cytokines was appropriate. The manuscript is well-written, and the methodology is sound.

Although the paper reports interesting outcomes, it has quite important weaknesses.

1) One of the major issues is the small, cross-sectional cohort (limiting statistical power and causal inference), the absence of independent validation or direct human translation. One interesting aspect of the paper though, is the spike in experiment, where the author shows that nanoparticle corona preserves the quantitative differences and fold changes and is truly representing the serum quantities of proteins.

Response: We agree with the reviewer's comments about the study design and appreciate acknowledgement of the quantitative benchmarking component of the study – this was indeed a critical experiment for building our own confidence in this workflow. We agree that the small, cross-sectional cohort limits statistical power, precludes causal inference, and does not allow for direct validation or immediate human translation. Below we address each limitation in more detail, including 1) human translation, 2) independent validation, and 3) sample size and study design. We have included additional discussion, experiments and results to address some of these limitations, including new results/figures exploring human relevance and independent validation of biological changes with existing literature. With regard to #3, we also discuss ongoing work on a much larger study for more definitive biological and longitudinal insights.

1) **Human translation -** We appreciate the suggestion and agree that it would be of value to evaluate the relevance of these findings in humans. To address the comparison with existing human aging data, the revised manuscript incorporates a cross- species comparison between the mouse proteomic signatures identified in this study and human plasma proteomics from Baltimore Longitudinal Study of Aging (BLSA) and published UK Biobank proteomics that support the translational relevance of the mouse data.

The newly generated analysis has been included in the results as a main figure (**Fig. 6**). Our analysis reveals that 50% of the age-associated proteins identified in this mouse study are also significantly age-associated in the BLSA. Interestingly, these results suggest a common circulating aging signature of extracellular matrix, endoplasmic reticulum, and lysosomal protein components in humans and mice. We assessed the association of these proteins with frailty, a key clinical aging endpoint, in BLSA and found that five significantly associated with frailty: COL6A3, LBP, PIGR, IGFBP1 and HPX. By far, the ECM protein COL6A3 is the most significant among these (adj. p-value = 5.07e-16). These findings are consistent with recent evidence of age-associated increase in ECM components in Nature Aging (PMID: 41023316), and a broad range of literature associating COL6A3 (aka Endotrophin, the uncleaved precursor) with mortality and chronic diseases (PMID 38871093). Notably, COL6A3 is among the top 5 proteins associated with pre-frailty, and frailty, in the UK Biobank (PMID 39535692), independently validating the association with human frailty observed in BLSA. Overall, these results support clinical relevance of the findings of our study and demonstrate the mouse study can shed light on human aging. We have included the new analysis into the results, figures, and discussion, accordingly, as shown below.

New Figure, **Fig. 6**:

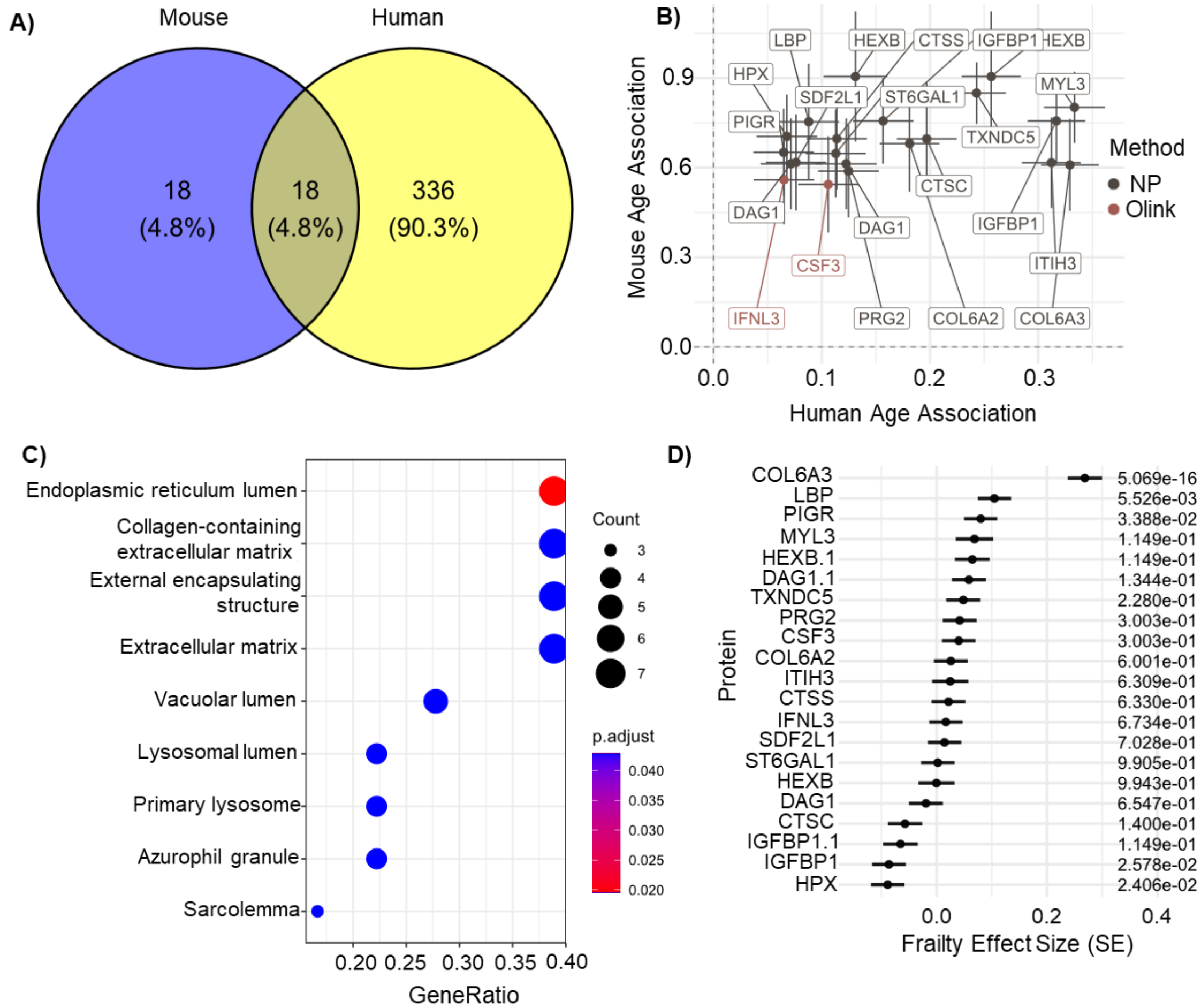

"Figure 6: Circulating proteins associated with age and frailty in mice and humans A) Venn diagram illustrating proteins identified as positively age-associated using Pearson linear modeling across NP-MS workflow and the O-link platforms in the cohort analyzed in this study (left blue), and proteomic measurements (SomaScan 7k assay) in the BLSA (right yellow), modeled as: age ~ protein + sex (Total proteins quantified across both datasets, N = 1742). B) Proteins significantly associated with age in mice and humans, determined using Pearson linear modeling (age ~ protein + sex). Color coding indicates detection method in this study. C) Overrepresentation analysis of proteins significantly upregulated with age in both species, annotated by cellular component. D) Associations between circulating proteins and frailty assessed using Pearson linear modeling across the cohort analyzed in this study and the BLSA, based on a 44-component frailty index and the model: frailty index ~ protein + age + sex. "

2) **Independent validation –** To address the need for independent validation of the aging changes measured in protein coronas we performed additional analysis and comparisons with existing literature. Targeted cytokine measurements with the Proximity Extension

Assay, and antibody-affinity-based platform, were a major component of this study. To perform a cross-platform comparison of a protein measured by both methods, nanoparticle-based MS versus antibody-based, we searched for overlapping protein observations between datasets and found a single protein – IL16 – with enough observations across mice to perform a comparison (Fig. 3C-D, 11 mice with matched measurements in both platforms). Using linear modeling, we found significant correlation between platforms in the measurement of IL16 ($p=0.0026$, $R = 0.81$). This example is a minor supportive factor in our claim of accurate representation of protein levels in the protein corona when compared with the original serum samples. To further validate whether our observed changes align with expected biology and proteomic measurements from prior studies, we performed an analysis comparing our measured changes to two published MS-based proteomic datasets in mouse blood that did not use nanoparticles. Although there are no prior mouse aging studies in blood to draw from, the two published studies examined sex-associated changes in mouse serum (PMIDs 37910662 and 34050187). Notably, with the exception of two proteins (Transcobalamin-2 and Hepatocyte growth factor activator) out of thirty-two, all sex-associated proteins from our study that were also reported in either published study were directionally aligned and correlated. This has been added as a supporting figure, **Fig. S3**, and is numerically detailed in Table S4. Overall, these findings further support that protein corona measurements in our study align with direct measurements in blood samples and accurately reproduce expected biological changes, such as sex-dimorphism.

New figure, **Fig. S3**:

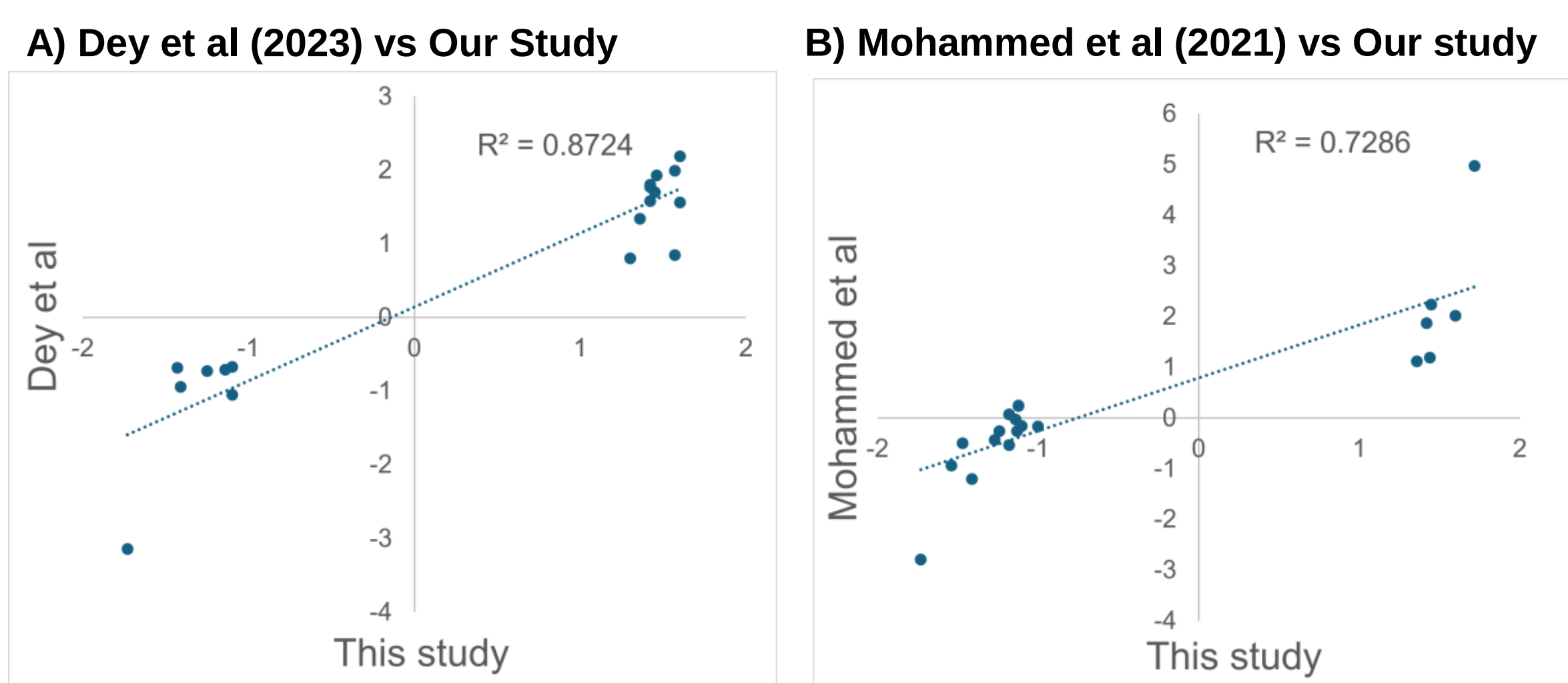

"**Supplementary Fig. 3- Correlation of sex-associated circulating proteins between this study and published datasets.** Correlation plots depicting sex-associated changes in our study versus those reported in A) Dey et. al. (PMID: 37910662); and B) Mohammed et. al. (PMID- 34050187). Proteins in both studies show strong positive correlations with this study (R^2=0.8724 and R^2=0.7286, respectively), and consistent directionality of sex associated effects."

Supplemental Table 4: Validation of top sex associated proteins with other study

Sl. No.	Gene	Uniprot ID	Description	SLAM<br>B-sex	Dey et. al. JPR,2023<br>log2(M/F)	Mohammed et. al. NPJ Syst. Biol. Appl. 2021<br>log2(M/F)
1	Serpina1e*	Q00898	Alpha-1-antitrypsin	**1.72**		**4.97**
2	C8g*	Q8VCG4	Complement component C8 gamma chain	**1.6**	**1.56**	**2.02**
3	C6	E9Q6D8	Complement component 6	**1.6**	**2.19**	
4	C7	D3YXF5	Complement component 7	**1.57**	**1.99**	
5	C8b*	Q8BH35	Complement component C8 beta chain	**1.45**	**1.7**	**2.24**
6	C8a*	Q8K182	Complement component C8 alpha chain	**1.42**	**1.77**	**1.87**
7	Ces3a	Q63880	Carboxylesterase 3A	**1.57**	**0.85**	
8	Mug1*	P28665	Murinoglobulin-1	**1.44**		**1.19**
9	Mup20	Q5FW60	Major urinary protein 20	**1.46**	**1.93**	
10	Crisp1	Q03401	Cysteine-rich secretory protein 1	**1.42**	**1.8**	
11	Mup3	P04939	Major urinary protein 3	**1.42**	**1.58**	
12	Egfr*	Q01279	Epidermal growth factor receptor	**1.36**	**1.34**	**1.12**

13	Serpina3k	P07759	Serine protease inhibitor A3K	1.3	0.8	
14	Tcn2	O88968	Transcobalamin-2	-1.12		0.25
15	Glycam1*	Q02596	Glycosylation-dependent cell adhesion molecule 1	-1.73	-3.14	-2.79
16	Adipoq*	Q60994	Adiponectin	-1.54		-0.94
17	Bche	Q03311	Cholinesterase	-1.47		-0.5
18	Serpina6*	Q06770	Corticosteroid-binding globulin	-1.41	-0.94	-1.2
19	Scg3	P47867	Secretogranin-3	-1.43	-0.68	
20	Ctsc	P97821	Dipeptidyl peptidase 1	-1.1	-1.05	
21	Afm	O89020	Afamin	-1.18		-0.53
22	Gsn	P13020	Gelsolin	-1.1		-0.16
23	Hgfac	Q9R098	Hepatocyte growth factor activator	-1.18		0.07
24	Igfals	P70389	Insulin-like growth factor-binding protein complex acid labile subunit	-1.27		-0.43
25	Itih1	Q61702	Inter-alpha-trypsin inhibitor heavy chain H1	-1.14		-0.03
26	Itih2	Q61703	Inter-alpha-trypsin inhibitor heavy chain H2	-1.13		-0.26
27	Itih4	A6X935	Inter alpha-trypsin inhibitor, heavy chain 4	-1		-0.17
28	Lum	P51885	Lumican	-1.24		-0.26
29	Psmb4	P99026	Proteasome subunit beta type-4	-1.12		0.24
30	Pi16	Q9ET66	Peptidase inhibitor 16	-1.1	-0.67	
31	Retn	Q99P87	Resistin	-1.25	-0.73	
32	Igfbp5	Q07079	Insulin-like growth factor-binding protein 5	-1.14	-0.71	

Red- Higher in males; **Blue**- Higher in Females; *-97% (C-statistics) discrimination between males and females (Mohammed et. al. 2021)

3) Sample size and study design - As the reviewer recognized, this study was not powered for identifying robust phenotypic changes and biomarker associations in SLAM. Instead, the study was designed to demonstrate a proteomic workflow that enables unprecedented proteomic blood biomarker studies in mice in strictly volume limited conditions and showcase its application in the context of aging. The key strength/contribution of the study is that it introduces a method for comprehensive serum proteomic analysis in mouse cohorts, which are notably missing from existing literature due to the technical challenges associated with such studies. This workflow opens the door to comprehensive serum proteomic studies in mice even in volumes in low as 10 µl (11 µl for the addition of cytokines), a volume requirement that is compatible with longitudinal collection of blood in mice. This volume can also realistically be used for proteomics without consuming an entire serum volume and is thus compatible with studies that utilize blood for multiple different measurements (e.g. 'clinical' labs, proteomics, metabolomics, etc.). Although the current study is not powered to detect phenotypic changes, it is sufficiently powered to identify a larger number of age- and sex-associated proteins in serum than previously described (adjusted p-value < 0.05), reveal interesting aging biology, and potential clinical relevance, as evidenced by our addition of an additional analysis of a subset of protein changes that are conserved with humans (Fig. 6). In order to emphasize the strengths of the study in this framing, we have made the following adjustments:

page-14, line-424:

"Nonetheless, the NP-MS workflow demonstrated strong reproducibility and sensitivity, enabling robust detection of age- and sex--associated signatures. Importantly, this workflow was successful within strict sample volume requirements down to 10uL. Therefore, this method is compatible with the volume limitations of longitudinal mouse studies and can be easily adapted and scaled into large cohort studies with a longitudinal study design. Future phases of the SLAM, incorporating larger sample sizes and true longitudinal sampling will be essential to validate these associations, improve statistical power and generalizability, and more accurately capture temporal relationships and potential causal effects across the lifespan."

We also completely agree that a larger study powered by the identification of longitudinal changes and phenotype-associated biomarker signatures will be of ultimate interest. In fact, a separate, larger study powered for identification of phenotype-associated biomarker signatures is ongoing within our laboratories, and will ultimately demonstrate more detailed biomarker signatures, proteomic clocks, and multi-omic associations in a larger subset (n=900) of SLAM, with up to 6 longitudinal timepoints per animal. Because this is an ongoing, multi-team study that integrates multiple teams and datasets, it is outside the scope of the current manuscript. However, we are happy to share that the proteomic data acquisition for the larger SLAM study was completed using the method described in this manuscript within 3 months, and with similar proteomic depth (>4000 cumulative proteins and ~2400 proteins per each 20ul serum sample), so we are confident in the scalability and applicability of the method described in this paper in a larger longitudinal mouse study.

To boost the significance of the current study, we would be happy to provide an additional figure summarizing the surface-level results of the larger SLAM study on request, including protein counts, list of protein/peptide identifications, and instrument time. These results would further demonstrate the scalability of this method and applicability in a longitudinal study for the current manuscript. However, given that the larger proteomic dataset is committed to an ongoing study, the underlying quantitative data from each sample would not be shareable at this time.

To focus our manuscript on the key contributions of the study and temper the conclusions from marginally significant findings, we present our biological findings as hypothesis-generating rather than definitive and emphasize the need for confirmation in larger and longitudinally sampled cohorts, page- 11, line- 323:

"However, because the proteomic data derives from a cross-sectional subset of the longitudinal SLAM cohort, age-associated signatures reflect population-level differences rather than within- individual aging trajectories. All biological interpretations are therefore framed conservatively; and the results should be viewed as foundational and hypothesis-generating rather than mechanistic or causal.".

Page-14, line-445:

"Overall, while these findings are cross-sectional, they establish a robust methodological framework to enable longitudinal and mechanistic investigations of aging and biomarker discovery in sample-limited mouse studies, and demonstrate translation relevance to human health."

Overall, we hope these results provide a strong methodological foundation for future studies in larger mouse cohorts and for eventual translation to human biomarker research, where quantitative accuracy is essential.

2) Finally, the critical roles of metabolite and lipid variations and their effects on protein corona have not been considered (several reports revealed that variations in metabolites and lipid profiles of plasma proteomics can substantially change the protein corona profile at the surface of nanoparticles); therefore, it is not clear that the observed variations originate from plasma proteome variations or reflection of metabolomics and lipidomic profile variation in plasma that caused changes in the composition of protein corona. This could be very important, as the authors were interested in proteomics signatures associated with glucose and body fat.

Response: This is an important point, we appreciate the importance of accounting for potential matrix effects, particularly metabolite and lipid profiles, on the protein corona. We hope the combination of responses presented below sufficiently address the underlying concerns of the reviewer.

First, we contend that the 'gold standard' method to demonstrate whether changes in the protein corona are representative of 'real' changes in the original serum samples at the proteome level (across a wide range of proteins) is to perform quantitative benchmarking (PMID: 27701404) on the same nanoparticles utilized in our study. This concern motivated us to conduct our own independent benchmarking study, shown in **Fig. 2A-B** (see below). Given that human and bovine blood are distinct in their compositions (including metabolites and lipids), if lipids/metabolites had a significant confounding effect on the composition of the protein corona, our expectation would be that our observed protein ratios would be skewed and inaccurate. However, our data suggests that on average the quantitative ratios of proteins in the protein corona are representative of true ratios of the original blood samples across a range of dilutions (5% - 100%).

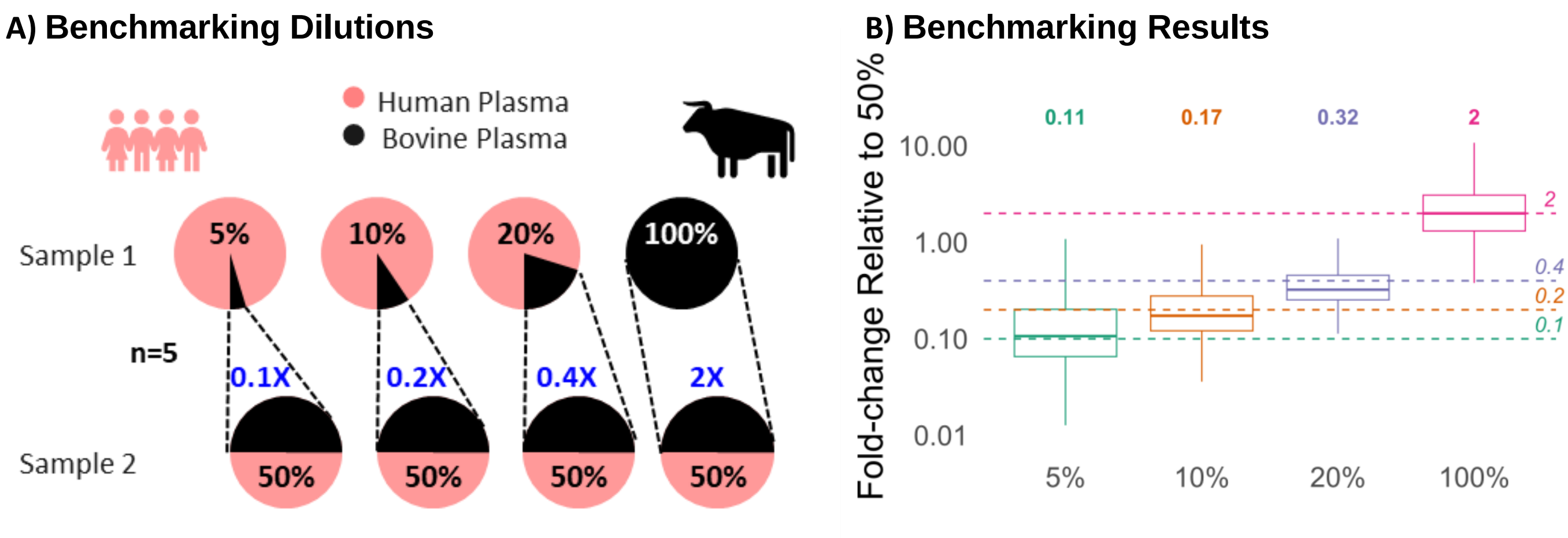

Figure 2: Qualitative and quantitative evaluation of proteomic data generated NP-coupled MS workflow. A) A quantitative benchmarking study was performed using five fixed ratios of human and bovine plasma (n=5 each). B) Boxplot showing the measured peptide ratios in each dilution versus the 50% bovine plasma sample. Dotted lines depict the expected ratios.

Second, we emphasize that published literature, which has more rigorously and comprehensively examined characteristics of the protein corona and binding properties of the nanoparticles utilized in this study, suggests that the effect of lipids and matrix do not significantly impact quantitation. We note that the nanoparticles used in this study are distinct from the plain polystyrene nanoparticles in the study cited by Reviewer #1 (PMID: 39511193) and may not exhibit the same binding behavior. The surface-functionalized superparamagnetic iron nanoparticles in this study are characterized in more detail elsewhere (PMID: 35275789). Lipid spike-in experiments have been done on the same nanoparticles we used, showing that spiking varying amounts of lipid do not significantly affect the number/intensity of proteins (PMID: 32699280, Nature Comms - last paragraph of p5 & first on p6, Fig. S4 and S5). Another paper in JPR (PMID: 41105214) reported on the minimal impact of background matrix (presumably with different lipid/metabolite compositions) on quantitative accuracy. We have noted these additional studies in the discussion (quoted below).

We explained now in the revised “Discussion” section for more clarity, page-10 , line-295:

“Concerns have been raised that NP- coronas may be sensitive to non-protein serum components, such as lips or metabolites, potentially introducing variability unrelated to biological differences. While recent work has shown that small molecules can influence protein corona

composition derived from plain polystyrene nanoparticles (40), multiple independent studies of the surface-functionalized superparamagnetic iron nanoparticles employed in this study suggest that these effects are not significant. Lipid-spike-in experiments using the same nanoparticles showed no appreciable changes in corona protein identities or intensities (15). Similarly, a prior report suggests minimal impact of background matrix on quantitative accuracy (41). Our own quantitative benchmarking data further supports this conclusion, showing high reproducibility and accurate quantification of known fold-changes in samples generated from mixing human and bovine blood, each containing a distinct composition of lipids, metabolites, and other components, in a range of fixed dilutions spanning 5% to 100% (Fig. 2B). Together, these findings indicate that matrix-derived components did not significantly alter corona composition in a manner that distorts relative protein quantification in this study and mitigate concerns matrix effects introduced variability unrelated to aging."

Third, we refer to our earlier response to your first comment on independent validation. Since our findings reproduce known biology, as demonstrated by the strong alignment with previously published proteomic studies, our confidence in the measured proteomic abundances in the protein corona is further strengthened (detailed in part 2 of the response to your first comment above).

Together, these data support that the nanoparticle system used in this study is relatively robust to lipid/metabolite variability, and that the proteomic differences we report most likely reflect underlying biological variation rather than matrix-driven artifacts.

3) I think in page 6, Supplementary Figure 2A is wrongly cited, as it does not show the CV.

Response: We appreciate the reviewer's careful reading of the manuscript. Supplementary Figure 2A is indeed the correct citation, although it shows CV percentage rather than CV. **Supplementary Figure 2A** presents the coefficient of variation (CV) distribution across technical triplicates for NP2, which is the dataset referenced in the corresponding sentence. We have reviewed the figure and text to ensure clarity and confirm that the citation accurately reflects where the CV data is displayed (8.649 or about 9.0%).

4) Overall, the figure quality is not satisfactory, for example, too many labels, font size consistency and axis lines should be corrected/added.

Response: We appreciate this comment and have adjusted figures accordingly to reduce the excessive text labels and make font sizes more consistent throughout.
